# PROTAC-Mediated Degradation of mHTT Aggregates Attenuates Neurotoxicity in Cellular and R6/2 Mouse Models of Huntington’s Disease

**DOI:** 10.1021/jacs.5c14078

**Published:** 2026-02-19

**Authors:** Po-Chao Lu, Yung-An Huang, Niaz Wali, Mei-Chun Tseng, Ruei-Yu He, Yijuang Chern, Tzu-Tang Wei, Jiun-Jie Shie, Joseph Jen-Tse Huang

**Affiliations:** † Institute of Chemistry, 38017 Academia Sinica, Taipei 115, Taiwan; ‡ Institute of Biomedical Sciences, Academia Sinica, Taipei 115, Taiwan; § Department and Graduate Institute of Pharmacology, College of Medicine, 33561 National Taiwan University, Taipei 100, Taiwan; ∥ Chemical Biology and Molecular Biophysics, Taiwan International Graduate Program, Academia Sinica, Taipei 115, Taiwan; ⊥ Department of Applied Chemistry, National Chiayi University, Chiayi City 600, Taiwan; # Neuroscience Program of Academia Sinica, Academia Sinica, Taipei 115, Taiwan

## Abstract

Huntington’s disease (HD) is a fatal neurodegenerative disorder caused by an expanded CAG repeat in the *HTT* gene, producing mutant huntingtin (mHTT) that misfolds into β-sheet-rich aggregates and drives neuronal loss. Current HTT-lowering strategies face challenges, including invasive delivery and nonselective suppression of wild-type HTT. Here, we report the development and synthesis of proteolysis-targeting chimeras (PROTACs) to selectively degrade aggregated mHTT. The lead compound, PROTAC **2′**, consists of a (pyridylvinyl)­aniline aggregate-binding ligand linked via polyethylene glycol spacers to pomalidomide, an E3 ligase recruiter for cereblon. PROTAC **2′** selectively degraded mHTT aggregates without affecting wild-type huntingtin and significantly reduced mHTT-induced cytotoxicity in the cell model. LC–MS/MS analysis confirmed the blood–brain barrier (BBB) penetration ability of PROTAC **2′** following subcutaneous administration. In an R6/2 HD mouse model, continuous PROTAC **2′** delivery via osmotic pumps improved body weight, motor coordination, and survival, correlating with reduced mHTT aggregation and neuroinflammation in the brain. These results highlight the therapeutic potential of aggregate-selective degradation as a disease-modifying strategy for HD, providing a promising alternative to conventional HTT-lowering approaches and supporting the broader potential of PROTAC-based therapeutics for neurodegenerative proteinopathies.

## Graphical abstract

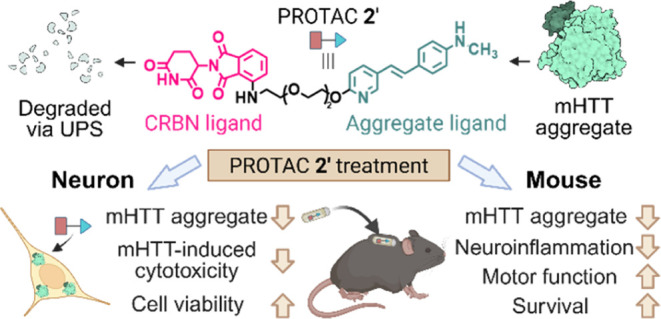

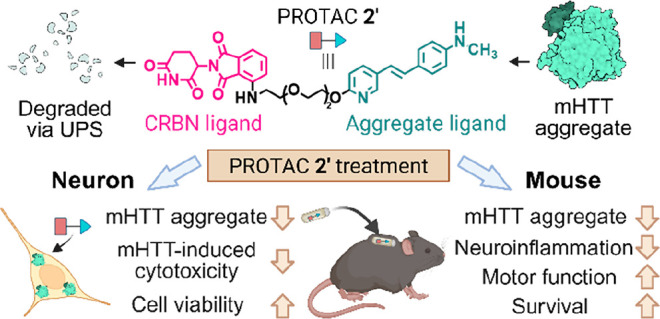

## Introduction

Huntington’s disease (HD) is a progressive and fatal neurodegenerative disorder characterized by motor dysfunction, cognitive decline, and psychiatric symptoms. It is caused by an abnormal expansion of CAG trinucleotide repeats in the *HTT* gene, leading to the production of mutant huntingtin (mHTT) with an extended polyglutamine tract. This misfolded protein tends to aggregate into β-sheet-rich oligomers and insoluble inclusions that accumulate in neurons,^1^ particularly within the striatum and cortex.^2^ These aggregates disrupt multiple cellular processes, including transcriptional regulation, mitochondrial function, and proteostasis, ultimately contributing to neuronal degeneration.^2^

Although HD is monogenic and its pathogenic protein is well-defined, no disease-modifying therapy has been approved to date. Current FDA-approved treatments, including tetrabenazine, deutetrabenazine, and valbenazine, only offer temporary symptomatic relief without altering disease progression.^3^ In recent years, considerable efforts have focused on developing disease-modifying therapies for HD that go beyond symptomatic management and aim to target either the production or accumulation of mHTT. These strategies include lowering mHTT expression at the genetic or transcript levels, enhancing its degradation, or modulating cellular proteostasis pathways. Among these strategies, RNA-targeting technologies, such as antisense oligonucleotides (ASOs)^4,5^ and RNA interference (RNAi),^6^ have shown promise in reducing mHTT levels in preclinical models and early-phase clinical trials. However, these approaches face critical challenges, including poor BBB penetration, the need for invasive delivery routes (e.g., intrathecal injection),^7^ and potential off-target effects from nonselective silencing of wild-type HTT, which is essential for neuronal development and survival.^8^ To avoid the unpredictable side effects of wild-type HTT loss, selective clearance of mHTT or its aggregates may provide a compelling alternative therapeutic approach.

To specifically reduce the level of targeted protein, proteolysis-targeting chimeras (PROTACs) have emerged as a powerful technology that enables neosubstrate degradation via the ubiquitin–proteasome system (UPS). These bifunctional molecules consist of a ligand that binds the protein of interest (POI) and another that recruits an E3 ubiquitin ligase connected by a chemical linker. By inducing the proximity between the two components, PROTACs promote ubiquitination and subsequent proteasomal degradation of the target protein. Unlike occupancy-based inhibitors, PROTACs act catalytically and substoichiometrically, offering enhanced potency, prolonged duration of action, and reduced off-target toxicity.^9^ While PROTACs have been extensively explored in oncology,^10^ their application in neurodegenerative diseases is still emerging.^11–16^ Given that mHTT aggregates display distinct β-sheet-rich conformations absent in the wild-type protein, we hypothesized that amyloid-specific degradation could allow for selective targeting of the toxic species while sparing essential wild-type HTT.

In this study, we report the design and functional validation of an amyloid-specific PROTAC that selectively binds mHTT aggregates and recruits the E3 ligase cereblon (CRBN) via pomalidomide (POM), as shown in Figure 1A. This PROTAC effectively reduces mHTT aggregates in Neuro2a (N2a) cells and in the HD mouse brain, leading to improved motor performance, reduced neuroinflammation, and extended survival. Our findings highlight the therapeutic potential of aggregate-selective PROTACs as a disease-modifying strategy for HD.

**Figure 1:**
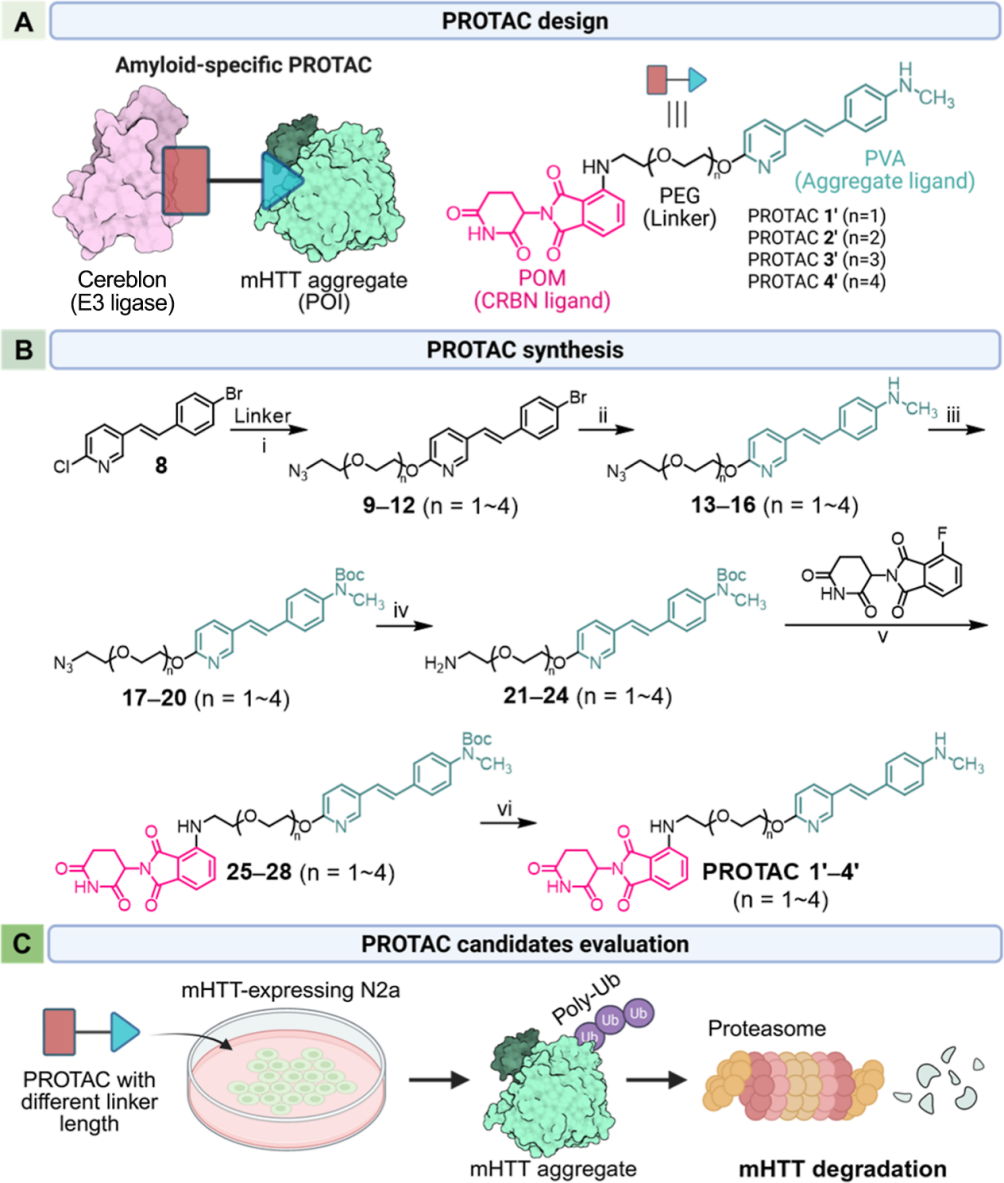
Design of PROTAC molecules against mHTT aggregates for HD therapy. (A) Schematic representation of the amyloid-specific PROTAC and its binding targets. POM = pomalidomide. PEG = polyethylene glycol. PVA = (pyridylvinyl)­aniline. CRBN = Cereblon. (B) Synthetic process of PROTACs **1′**–**4′**. Reagents and reaction conditions: (i) N_3_CH_2_CH_2_(OCH_2_CH_2_)_n_OH, NaH, 1,4-dioxane, reflux, 21 h; 69–74%. (ii) CH_3_NH_2_, 10 mol % copper, aq. C_2_H_5_OH, 105 °C, 18 h; 68–73%. (iii) Boc_2_O, Et_3_N, C_2_H_5_OH, rt, 7 h; 90–99%. (iv) PPh_3_, aq. THF, rt, 15 h; 96–97%. (v) *i*Pr_2_NEt, NMP, 90 °C, 18 h; 47–61%. (vi) CF_3_CO_2_H, CH_2_Cl_2_, rt, 2 h; 76–86%. NMP = *N*-methyl-2-pyrrolidone. Boc_2_O = di-*tert*-butyl decarbonate. (C) Conceptual scheme of functional validation experiments of PROTAC candidates in this study.

## Results

### Design and Synthesis of PROTAC Molecules Against polyQ Aggregates

Following the conceptual framework outlined above, the practical implementation of an effective PROTAC requires careful coordination of three elements: an aggregate-recognizing POI ligand, an E3 ligase-recruiting ligand, and a linker that enables productive ternary complex formation. In this context, linker length, composition, and attachment geometry are particularly important, as they influence both degradation efficiency and physicochemical properties beyond simple binary binding affinity. Given the lack of a well-defined catalytic pocket in mutant huntingtin (mHTT), our design strategy focused on selective recognition of aggregated polyglutamine species, which adopt β-sheet-rich conformations and accumulate as toxic oligomers and insoluble aggregates.^17^

To identify a suitable POI-binding element for mHTT aggregates, we evaluated small-molecule motifs known to bind amyloid structures. Because PET tracers specifically developed for aggregated mHTT are not yet available, we considered FDA-approved amyloid-β imaging agents, including [^18^F]­AV-45,^18^ [^18^F]­BAY-94-9172,^19^ and [^18^F]­GE067.^20^ The tau tracer [^18^F]­AV-1451 was excluded due to its inability to detect nontau aggregates.^21,22^ As these amyloid tracers exhibit comparable blood–brain barrier permeability and amyloid-binding affinity,^23^ scaffold minimization was used as a guiding principle, leading to the selection of the [^18^F]­AV-45-derived (pyridylvinyl)­aniline (PVA) motif as the aggregate-targeting POI ligand (Figure 1A). For E3 ligase recruitment, POM was incorporated based on its established binding to CRBN (Figure 1A).^24^ To connect the POI-binding PVA motif and the CRBN-recruiting POM ligand, polyethylene glycol (PEG) linkers were introduced to improve aqueous solubility, modulate lipophilicity, and allow systematic control over the spatial relationship between the two binding elements.^25^

Using this modular design strategy, we synthesized a series of PROTACs (**1′**–**4′**) in which PVA and POM were tethered by PEG linkers containing 2 to 5 ethylene glycol units (Figure 1B). The synthesis of PROTACs **1′**–**4′**, featuring PVA and POM moieties tethered by the linkers containing 2–5 units of ethylene glycol, was accomplished, as described in Figure 1B. The intermediates **9**–**12** were synthesized via a C–O coupling reaction between halogen-substituted styrylpyridine **8** and the corresponding PEG linker N_3_CH_2_CH_2_(OCH_2_CH_2_)_n_OH in the presence of NaH. The hydroxy group of PEG linkers was selectively attached to the 2-position of styrylpyridine, followed by a copper-mediated coupling reaction with methylamine in aqueous ethanol to form *N*-methylanilines **13**–**16**, which serve as the core structure of PVA. Compounds **13**–**16** were reacted with Boc_2_O and Et_3_N in ethanol at room temperature to afford the *N*-Boc derivatives **17**–**20** in 90–99% yield. A subsequent Staudinger reduction reaction using PPh_3_ in aqueous THF was performed at room temperature to reduce the azide group, resulting in the amine compounds **21**–**24** in 96–97% yield. The C–N coupling reaction of compounds **21–24** with 4-fluoro thalidomide was carried out by using Hünig’s base in *N*-methyl-2-pyrrolidone (NMP). The final PROTACs **1′**–**4′** were obtained by removing the Boc-protecting group under acidic conditions.

This modular approach enabled systematic variation of linker length while maintaining constant POI-binding and E3 ligase-binding elements, allowing direct evaluation of linker-dependent degradation performance in mHTT-expressing N2a cells (Figure 1C).^26^ Detailed synthetic procedures and full characterization of all intermediates and final compounds are provided in Supporting Information Schemes S1 and S2.

### PROTAC 2′ Selectively Degrades mHTT Aggregates in Cells

To evaluate the degradation ability of PROTAC candidates, each candidate was treated individually with HTT­(Q)_109_-eYFP-expressing N2a cells (Figure 2A). Confocal microscopy revealed that treatment with PROTAC **2′** significantly reduced the percentage of cells with mHTT aggregates compared to the control and other PROTAC candidates (Figure 2A,B). This reduction was observed at a noncytotoxic concentration of 1 μM, as confirmed by cell viability assays (Figure S1A–D). To further validate these findings, we performed a Western blot analysis to quantify the levels of aggregated mHTT following treatment. As shown in Figure 2C,D, PROTAC **2′** (lane 4) substantially decreased the amount of insoluble HTT­(Q)_109_-eYFP compared to PROTAC **1′** (lane 3), PROTAC **3′** (lane 5), and PROTAC **4′** (lane 6). In contrast, untreated cells transfected with HTT­(Q)_109_-eYFP (lane 2) exhibited a marked accumulation of aggregates relative to the nontransfected control (lane 1). Importantly, the levels of soluble HTT­(Q)_109_-eYFP remained consistent across all treatment groups (Figure 2C,E), indicating that our PROTACs preferentially target the aggregated species while sparing the soluble form. These results demonstrate that PROTAC **2′** effectively promotes the degradation of mHTT aggregates in cells, supporting its potential as a selective tool for targeting pathogenic protein inclusions.

**Figure 2:**
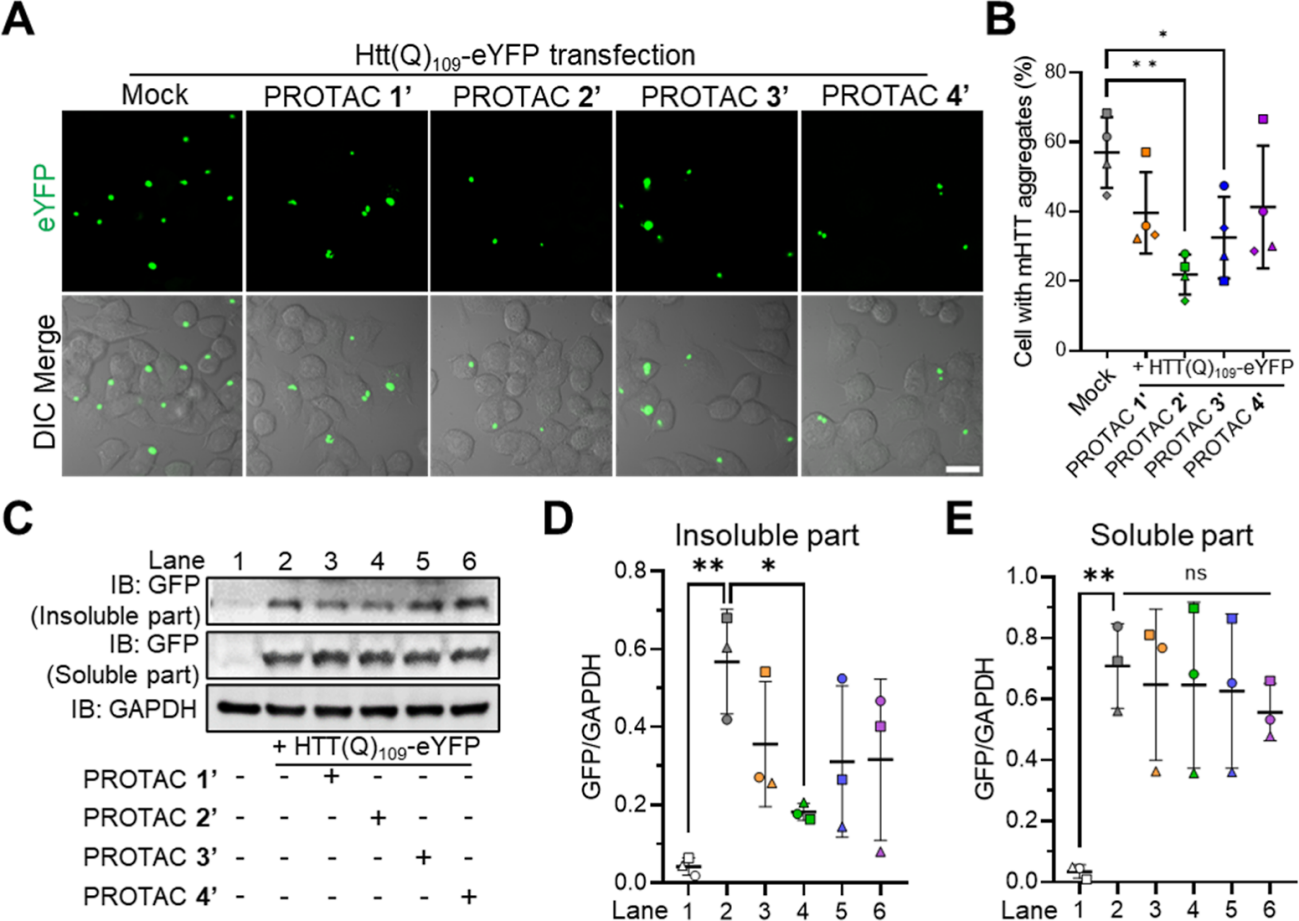
Evaluation of PROTAC candidates in reducing mHTT aggregates and alleviating mHTT-induced neurotoxicity. (A) Representative images of HTT­(Q)_109_-eYFP-expressing N2a cells treated with PROTAC **1′**–**4′** (1 μM) individually. Scale bar = 20 μm. (B) Quantification of remaining aggregate puncta in panel A (*N* = 4, each shape represents an independent experiment). (C) Western blot of HTT­(Q)_109_-eYFP-expressing N2a cells treated without or with either PROTAC **1′**–**4′** (1 μM). **(D,E)** Quantification of the blots showing the insoluble (D) and soluble (E) parts of HTT­(Q)_109_-eYFP (*N* = 3, each shape represents an independent experiment). All statistical results were quantified by ImageJ and shown as mean ± SD. Panels B, D, and E were analyzed by one-way ANOVA with Dunnett’s posthoc test (*P < 0.05, **P < 0.01, ***P < 0.001).

### PROTAC 2′ Specifically Removes mHTT Aggregates via Proteasomal-Degradation and Alleviates mHTT-Induced Cytotoxicity

Selective targeting of mHTT aggregates should be considered for therapeutic development against HD, as wild-type HTT plays vital roles in neuronal survival and cellular homeostasis.^7^ Given the superior activity of PROTAC **2′** in degrading mHTT aggregates, we next assessed its specificity and concentration-dependent efficacy. N2a cells expressing either HTT­(Q)_109_-eYFP or HTT­(Q)_25_-eYFP were treated with increasing concentrations of PROTAC **2′**, ranging from 0.01 to 10 μM (Figure 3A). Western blot analysis revealed that PROTAC **2′** effectively and selectively degraded HTT­(Q)_109_-eYFP aggregates in a dose-dependent manner (Figure 3A,B), with negligible effect on the levels of nonpathogenic HTT­(Q)_25_-eYFP (Figure 3A,C). To further verify that degradation was dependent on both the E3 ligase-binding and aggregate-binding domains of PROTAC **2′**, two control compounds (**JJS0434** and **JJS0435**) were prepared by selectively omitting the POM or PVA moiety from PROTAC **2′** (Figure S2A). Neither compound was able to reduce the number of HTT­(Q)_109_-eYFP aggregates (Figure S2B,C). Moreover, compound **26**, which contains a Boc group on the PVA moiety known to decrease its binding affinity to amyloid-β,^23^ also failed to decrease HTT­(Q)_109_-eYFP aggregates (Figure S2D–F). These results collectively indicate that both the E3 ligase-binding and aggregate-binding domains are essential for PROTAC **2′** activity. The detailed synthetic routes of compounds **26**, **JJS0434**, and **JJS0435** are provided in Supporting Information Scheme S2 and S3. Since PROTAC-mediated degradation occurs via the ubiquitin-proteasome system (UPS), we further tested whether proteasome inhibition could block PROTAC **2′**-induced clearance. Pretreatment with MG132, a proteasome inhibitor, significantly attenuated PROTAC **2′** activity and led to increased accumulation of HTT­(Q)_109_-eYFP aggregates compared to PROTAC **2′** alone (Figure 3D,E). In parallel, MG132 caused colocalization of PROTAC **2′** with HTT­(Q)_109_-mCherry puncta (bottom panel in Figure S3A,B), supporting that PROTAC **2′** binds to mHTT aggregates and facilitates their proteasomal degradation. Importantly, PROTAC **2′** exerted negligible effects on soluble HTT levels, underscoring its amyloid-specific action. Our findings supported that PROTAC **2′** promotes the degradation of mHTT aggregates via UPS.

**Figure 3:**
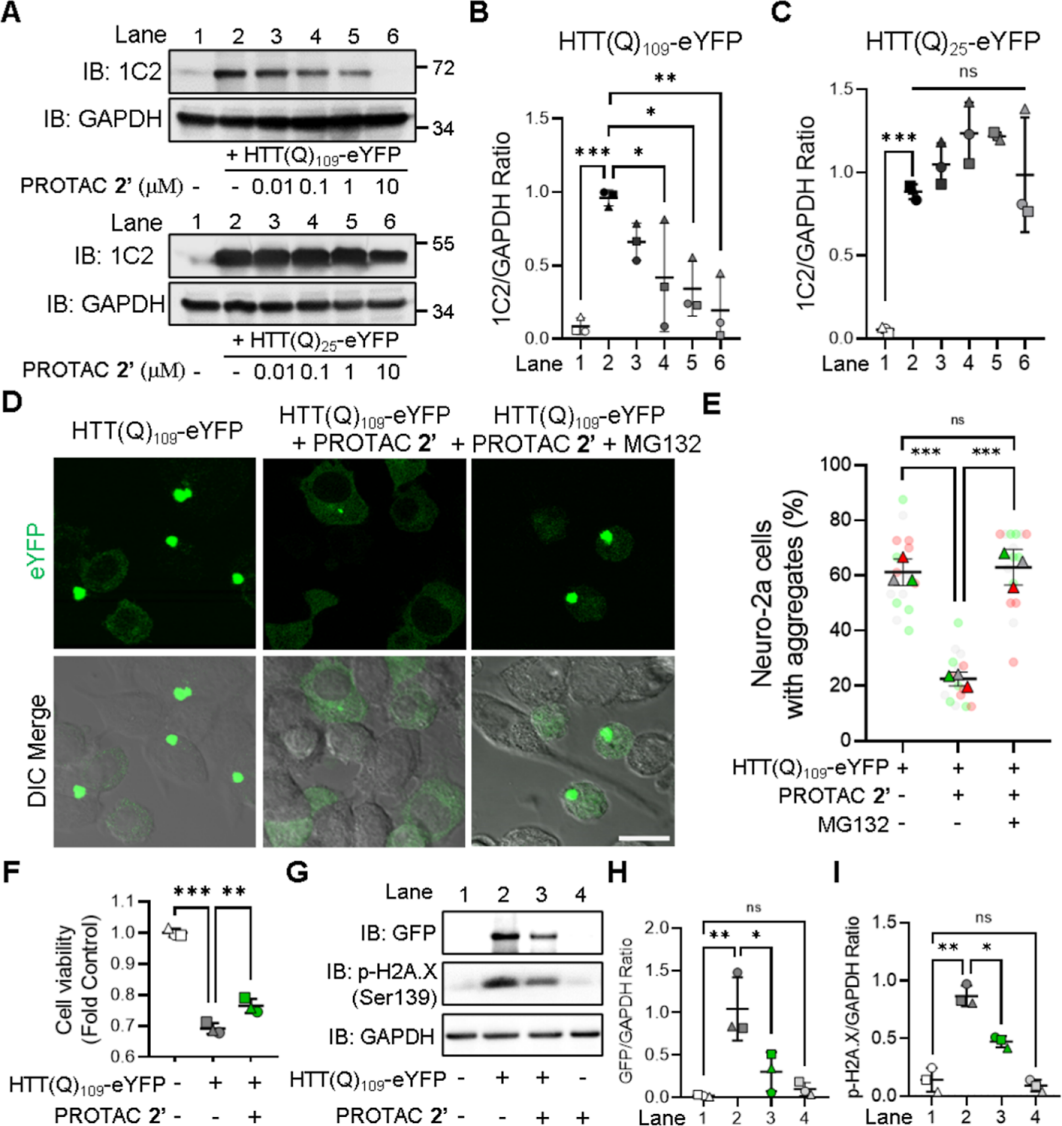
PROTAC 2′ specifically removes mHTT aggregates via proteasomal degradation and attenuates mHTT-induced cytotoxicity. (A) Western blot of HTT­(Q)_109_-eYFP (upper panel) and HTT­(Q)_25_-eYFP (lower panel) expressing N2a cells treated with various concentrations of PROTAC **2′**. (B,C) Quantification of the blots of HTT­(Q)_109_-eYFP (B) and HTT­(Q)_25_-eYFP (C) in panel A (*N* = 3, each shape represents an independent experiment). (D) Representative images of HTT­(Q)_109_-eYFP-expressing N2a cells in the presence and absence of PROTAC **2′** (1 μM) or/and MG132 (1 μM). Scale bar = 20 μm. (E) Quantification of the percentage of aggregate-positive N2a cells among HTT­(Q)_109_-eYFP-expressing cells in panel D (triangles represent an independent experiment, *N* = 3; dots represent the technical replicates within each *N*, *n* = 5). (F) AlamarBlue reduction assay of HTT­(Q)_109_-eYFP-expressing N2a cells treated with or without 1 μM PROTAC **2’** (*N* = 3, each shape represents an independent experiment) (G) western blot of HTT­(Q)_109_-eYFP expressing N2a cells treated with or without PROTAC **2′** (1 μM). **(H,I)** Quantification of GFP (H) and p-H2A.X (I) blots in panel G (*N* = 3, each shape represents an independent experiment). All the statistical results were quantified by ImageJ and shown as mean ± SD. Panel B and C were analyzed by one-way ANOVA with Dunnett’s posthoc test (*P < 0.05, **P < 0.01, ***P < 0.001). Panel E, F, H, and I were analyzed by one-way ANOVA with Tukey’s posthoc test (*P < 0.05, **P < 0.01, ***P < 0.001).

As we have proved that PROTAC **2′** was able to reduce mHTT aggregates, we further tested the beneficial effect of PROTAC **2′** in HTT­(Q)_109_-eYFP-expressing N2a cells. According to the alamarBlue reduction assay (Figure 3F), overexpression of toxic mHTT decreased the viability of N2a cells (the black bar) compared with the control (the white bar). In contrast, PROTAC **2′** treatment significantly enhanced the cell viability (the green bar) supporting its potential therapeutic effect against HD. By applying Western blot (Figure 3G), we observed that PROTAC **2′** was able to decrease the DNA damage marker^27^ (antiphosphor-H2A.X, Figure 3I) in response to the degradation of HTT­(Q)_109_-eYFP aggregates (anti-GFP, Figure 3H). Conclusively, PROTAC **2′**-mediated mHTT aggregate clearance significantly the relieved-mHTT induced cellular apoptosis pathway.

PROTAC 2′ crosses the Blood–Brain Barrier and sustains brain exposure following subcutaneous administration.

After confirming the beneficial effects of PROTAC **2′** in cellular models, we proceeded to evaluate its brain exposure and safety *in vivo*. To quantify PROTAC **2′** in brain tissue, the compound was extracted from brain homogenates and analyzed using liquid chromatography-tandem mass spectrometry (LC–MS/MS), as outlined in the workflow in Figure 4A. PROTAC **2′** showed a retention time of 1.2 min (Figure S4A), with a precursor ion at *m*/*z* 614.2626 and a mass accuracy of less than 5 ppm (Figure S4B). The corresponding MS/MS fragmentation spectra are presented in Figure 4B. To validate the extraction protocol, we evaluated the matrix effects, extraction recovery, and overall processing efficiency. Without significant matrix effect (102.8%), the extraction recovery was 85.9%, and the processing efficiency was 87.7%, based on triplicate analyses (Figure S4C). A calibration curve ranging from 1 to 50 ppb was established (Figure S4D), enabling the quantitative assessment of PROTAC **2′** levels in brain tissue following subcutaneous (SC) administration, a dosing route previously validated in an Alzheimer’s disease mouse model.^28^

**Figure 4:**
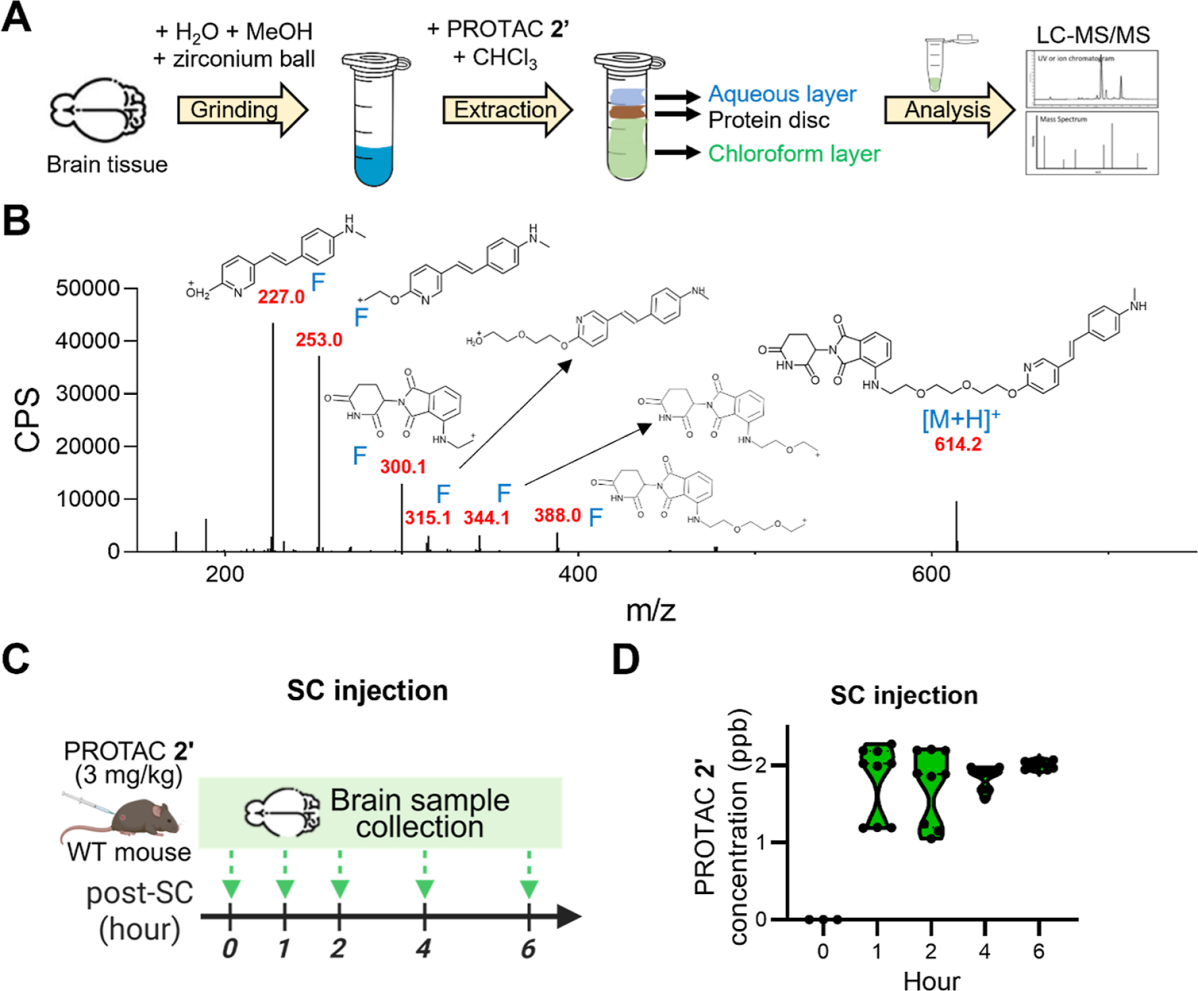
PROTAC 2′ crosses the blood–brain barrier and sustains brain exposure following subcutaneous administration. (A) Schematic diagram of PROTAC **2′** extraction and LC–MS/MS analysis from mouse brain homogenates. (B) MS/MS spectra of PROTAC **2′** obtained using multiple reaction monitoring (MRM) mode for quantification, with the selected fragment ion at *m*/*z* 227.0. (C) Schematic illustration of the experimental design to assess the BBB permeability of PROTAC **2′** via SC injection. (D) PROTAC **2′** concentration in WT mouse brains at different time points after SC injection (3 mg/kg), measured by LC–MS/MS (*n* = 9 per time point).

To evaluate brain penetration, wild-type (WT) B6CBA mice received a single SC dose of PROTAC **2’** (3 mg/kg). Brain tissues were harvested at 0, 1, 2, 4, and 6 h postinjection for LC–MS/MS analysis (Figure 4C). As shown in Figure 4D, PROTAC **2′** was consistently detected in brain tissue at all time points, maintaining an average concentration of approximately 1.96 ppb over 6 h. These results indicate that PROTAC **2′** crosses the BBB and sustains measurable brain exposure.

### PROTAC 2′ Exhibits Negligible Toxicity in Wild-Type B6CBA Mice Following Low-Dose and Long-Term Treatment

As BBB restricts more than 98% small-molecule drugs and all macromolecular therapeutics from access to the brain,^29^ safety issues of BBB-penetrable PROTAC **2′** should be addressed before clinical translation to avoid unintended side effects. Therefore, we next examined whether repeated PROTAC **2′** administration induced adverse effects in WT mice. WT mice received weekly SC injections of PROTAC **2′** (3 mg/kg) from 6 to 10 weeks of age, with vehicle-treated mice as controls (Figure 5A). As shown in Figure 5B, the body weight of PROTAC **2′**-treated mice was comparable to controls. We further evaluated whether PROTAC **2′** altered cortical thickness and striatal volume in WT mice brains following the published protocols (Figure S5A).^30^ Histological analysis of coronal brain sections (Figure S5B) revealed no structural abnormalities in the cortical thickness (Figure 5C) or striatal volume (Figure 5D). Similarly, the liver and kidney morphologies remained unchanged following treatment (Figure S5C). To assess potential systemic toxicity, we measured serum levels of liver enzymes [alanine aminotransferase (ALT) and aspartate aminotransferase (AST)] and kidney function markers [blood urea nitrogen (BUN) and creatinine (CRE)]. No significant differences were observed between the treated and control groups (Figure 5E,F), suggesting no evident hepatotoxicity or nephrotoxicity. Based on these findings, we proceeded with the 3 mg/kg/week SC dosing regimen for subsequent efficacy studies in HD mouse models.

**Figure 5:**
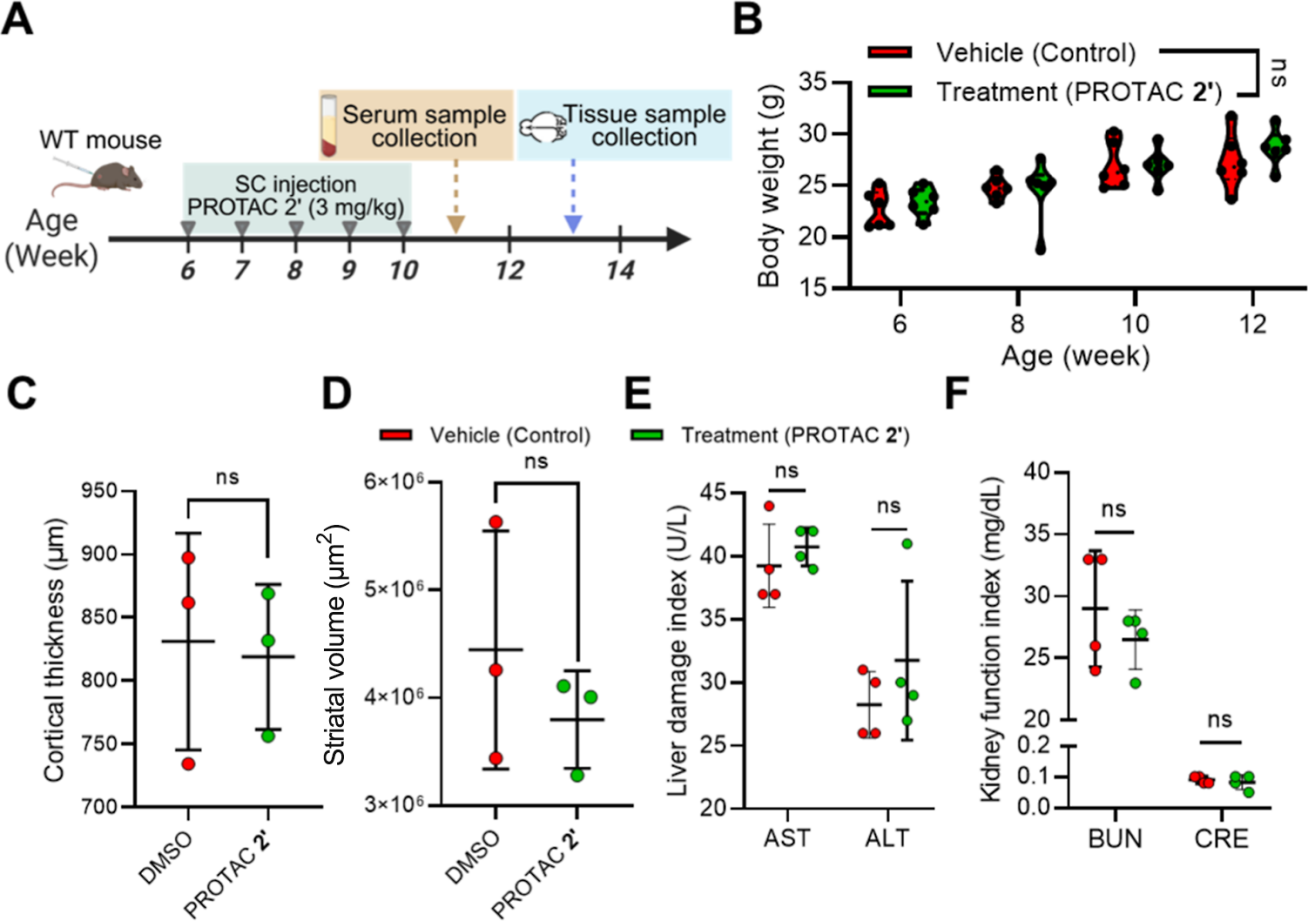
BBB-permeable PROTAC 2′ exhibits negligible toxicity in wild-type B6CBA mice during long-term treatment. (A) Schematic representation of the treatment procedure to evaluate the toxicity of PROTAC **2′** in wild-type B6CBA mice. Mice received weekly (total 5 weeks) SC injections of PROTAC **2′** (3 mg/kg, *n* = 6) or vehicle (DMSO, *n* = 6). Mouse samples, including serum, kidney, liver, and brain tissue, were collected for further analysis at the end point of the experiment. (B) Body weight was recorded every 2 weeks from the age of 6 to 12 weeks. (C,D) Quantification of (C) cortex thickness (*n* = 3) and (D) striatal volume (*n* = 3) in WT mice with or without PROTAC **2′** administration. Each dot represents the average value of an individual brain tissue. Representative images are shown in Figure S5A. (E) Liver function was assessed by measuring serum AST and ALT levels in mice administered either vehicle (DMSO, *n* = 4) or PROTAC **2′** (3 mg/kg, *n* = 4) at 11 weeks of age. Each dot represents the average value of an individual mouse serum. (F) Kidney function was evaluated by measuring serum BUN and CRE levels in the same treatment groups as described in panel E. All the statistical results were shown as mean ± SD. Panel B was analyzed by two-way ANOVA with Sidak’s multiple comparisons test. Panels C–F were analyzed by two-tailed unpaired *t*-test.

### PROTAC 2′ Improved Motility, Behavior, and Survival of R6/2 HD Mice

To evaluate the therapeutic effect of PROTAC **2′** *in vivo*, we implanted osmotic pumps containing either vehicle (DMSO) or PROTAC **2′** (3 mg/kg/week) subcutaneously on the backs of 6-week-old B6CBA-WT and B6CBA-R6/2 mice (see Materials and Methods for details). Osmotic pumps are rate-controlled drug delivery systems^31,32^ that reduce dosing frequency and potential side effects in long-term mouse studies.^32,33^ Following 4 weeks of continuous administration (from weeks 6 to 10), the therapeutic efficacy of PROTAC **2′** was evaluated using multiple behavioral and survival assessments (Figure 6A). As R6/2 mice typically begin to lose weight at 9 weeks due to mHTT toxicity,^34^ we monitored body weight starting from week 8. As shown in Figure 6B, vehicle-treated R6/2 mice displayed a progressive decline in weight, beginning at week 9 (orange line). In contrast, PROTAC **2′**-treated R6/2 mice showed a marked delay in weight loss, maintaining higher body weight until week 11 (orange dotted line), which corresponds to the period when most of the compound had been released from the pump. WT mice treated with either vehicle or PROTAC **2′** showed no significant difference in body weight (green and green dotted lines), suggesting that long-term, low-dose PROTAC **2′** treatment did not induce toxicity.

**Figure 6:**
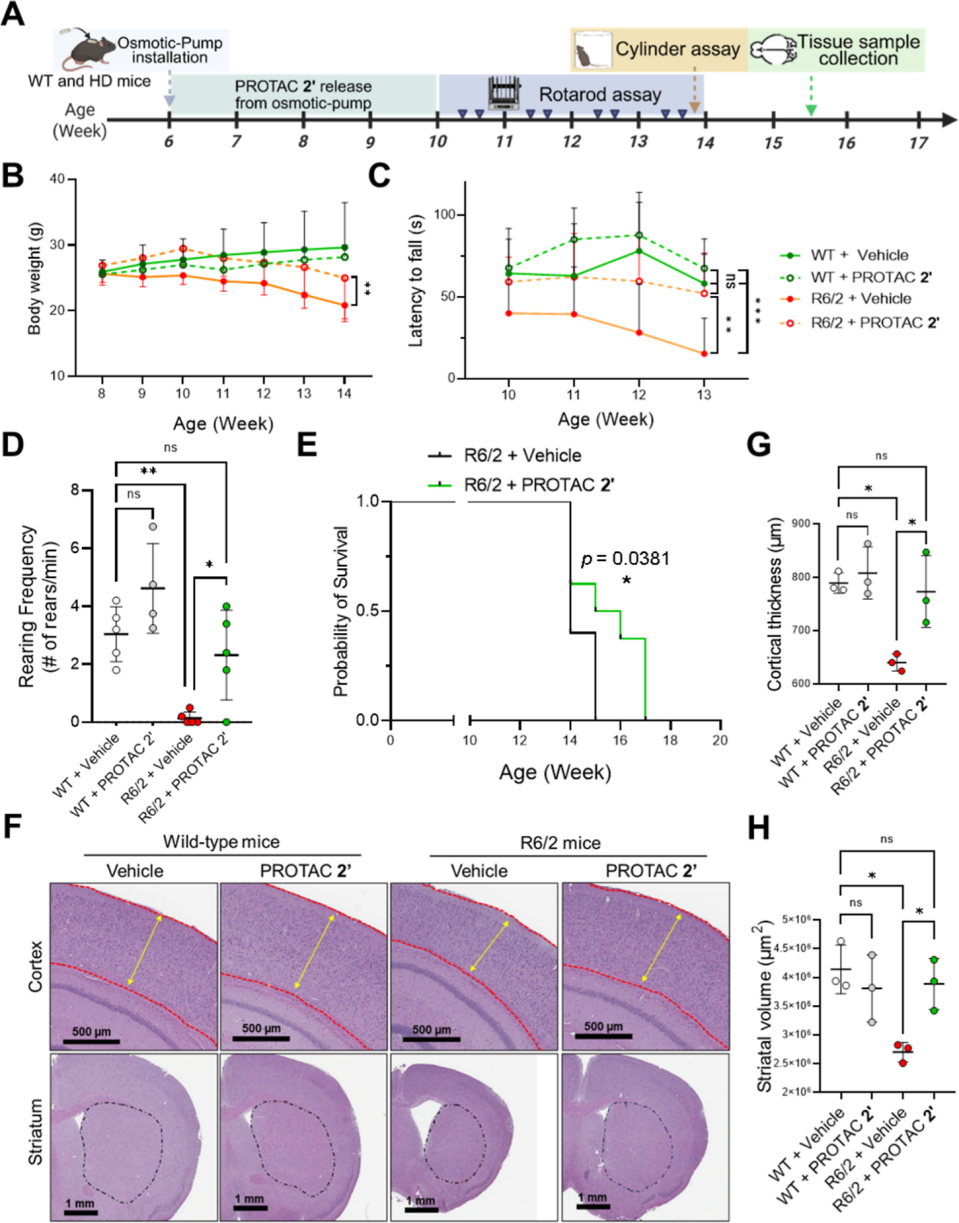
PROTAC 2′ administration by osmotic pumps improved motility, behavior, and survival of R6/2 HD mice. (A) Schematic diagram illustrating the experimental design of PROTAC **2′** administration by mini-pump in R6/2 HD mice. (B) Body weight was recorded once a week from the age of 8 to 14 weeks. (C) Rotarod performance was recorded from the age of 10 to 13 weeks (twice per week). B6CBA-WT (DMSO): *n* = 8, B6CBA-WT (PROTAC **2′**): *n* = 5, B6CBA-R6/2 (DMSO): *n* = 10, B6CBA-R6/2 (PROTAC **2′**): *n* = 8. Each dot in panels B and C represents the average value of each experimental group during a given week. (D) Rearing behavior accessed by a cylinder assay at the age of 14 weeks. WT + vehicle (*n* = 5), WT + PROTAC **2′** (*n* = 4), and R6/2 + vehicle (*n* = 5), and R6/2 + PROTAC **2′** (*n* = 5). Each dot in panel D represents the rearing frequency of an individual animal. (E) Kaplan–Meier survival rate of the R6/2 mice (vehicle, *n* = 10; PROTAC **2′**, *n* = 8). Black marks on the survival curves indicate animals that died naturally or were euthanized. (F) Representative H&E-stained images of WT and R6/2 mouse brain sections, including the cortex (reddish dotted line) and striatal (black dotted line) region. (G) Quantification of cortical thickness (yellow double-headed arrow) in panel F (*n* = 3). Each dot represents the average value of the shortest distance across the cortex of an individual brain tissue (see materials and methods for detail). (H) Quantification of the striatal volume (block dotted line) in panel F (*n* = 3). Each dot represents an individual brain tissue. All the statistical results were quantified by ImageJ and shown as mean ± SD. Panels B and C were analyzed by two-way ANOVA with Sidak’s multiple comparisons test (*P < 0.05, **P < 0.01, ***P < 0.001). Panels D, G, and H were analyzed by one-way ANOVA with Tukey’s posthoc test (*P < 0.05, **P < 0.01, ***P < 0.001). Panel E was analyzed by the Mantel–Cox test, which determined *P* = 0.0381 significance difference among experimental groups.

Motor function, which begins to decline around week 11 in R6/2 mice,^35^ was assessed using the rotarod apparatus starting from week 10. By week 13, vehicle-treated R6/2 mice showed a significant reduction in latency to fall (Figure 6C, orange line), indicating severe motor impairment. Remarkably, R6/2 mice treated with PROTAC **2′** led to a statistically significant improvement in rotarod performance of R6/2 (Figure 6C, orange dotted line), demonstrating that treatment attenuated motor decline. To corroborate these findings, we also evaluated rearing behavior by cylinder assay, a measure of spontaneous exploratory activity that is known to be impaired in R6/2 mice.^36,37^ PROTAC **2′**-treated R6/2 mice displayed a statistically significant higher frequency of rearing compared to vehicle-treated controls (Figure 6D, green vs red plot), further supporting motor improvement.

Finally, survival analysis revealed that all vehicle-treated R6/2 mice died by 15 weeks (Figure 6E, black line). In contrast, 50% of PROTAC **2′**-treated R6/2 mice survived to 16 weeks, and 35% of them survived to 17 weeks (Figure 6E, green line), indicating a statistically significant extension of lifespan. In summary, PROTAC **2′** is able to delay disease onset, improve motor function, and extend survival in the R6/2 HD mouse model.

### PROTAC 2′ Eliminates the mHTT Aggregates and Decreases Inflammatory Response in R6/2 HD Mouse Brain

Clinicopathological features of Huntington’s disease (HD) brain typically include marked atrophy of the cortex and striatum.^38^ To evaluate whether PROTAC **2′** treatment mitigates these pathological changes, we quantified cortical thickness and striatal volume in R6/2 mice and compared the results with those from WT controls. H&E staining showed that R6/2 mice treated with PROTAC **2′** exhibited significantly increased cortical thickness compared to vehicle-treated R6/2 mice, approaching the levels observed in WT mice (Figure 6F, indicated by the yellow double-headed arrow). Similarly, PROTAC **2′** treatment markedly increased striatal volume in R6/2 mice relative to vehicle controls, again showing partial restoration toward WT values (Figure 6F, indicated by black dotted lines). Statistical comparisons of the cortical thickness and striatal volume among the three groups are presented in Figure 6G,H.

Since behavioral deficits of HD mice are highly associated with mHTT aggregates in the cortex and striatum,^39^ we further investigated whether PROTAC **2′** affects the amount of mHTT puncta in R6/2 mouse brain by tissue immunofluorescence staining. With anti-mHTT antibody (EM48), we found that the mHTT puncta amounts were significantly decreased in both the cortex and striatum regions of the PROTAC **2′**-treated R6/2 mice (right panel of Figure 7A,B and C), as compared to that of the vehicle-treated controls (left panel of Figure 7A,B,C). Notably, PROTAC **2′** did not affect the remaining puncta size of PROTAC **2′**-treated R6/2 mice (Figure 7D). As WT mouse brains showed negligible background signal of mHTT antibody (Figure S6A,B), we concluded that PROTAC **2′** indeed lowered the mHTT puncta amount in both the cortex and striatum of R6/2 mouse brain.

**Figure 7:**
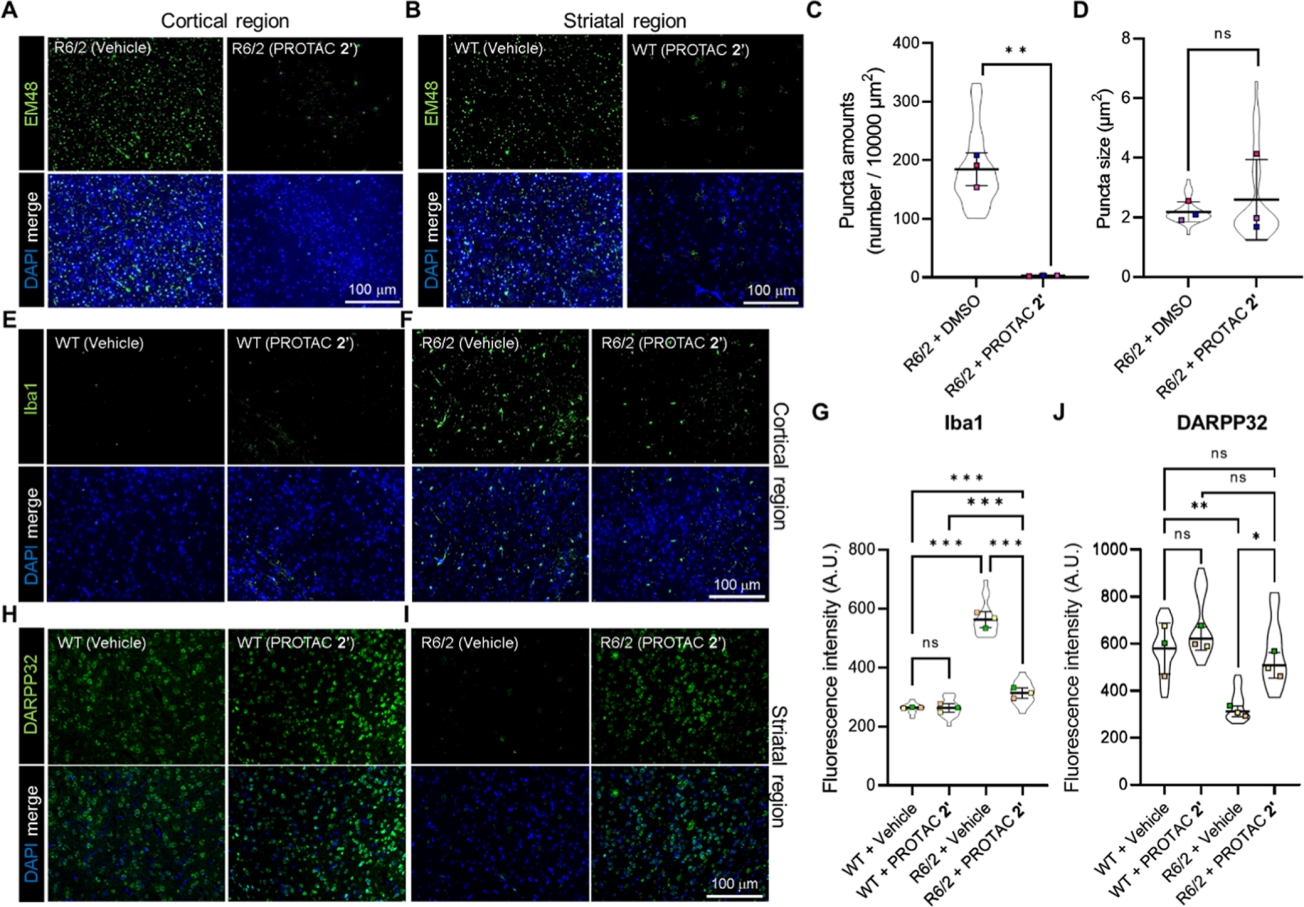
PROTAC 2′ administration by osmotic pump decreases mHTT aggregates and inflammation in the R6/2 mouse brain. Representative images of immunofluorescence staining (anti-EM48) of the cortical (A) and striatal (B) regions of the R6/2 mouse brain with or without PROTAC **2′** treatment. Quantification of mHTT puncta amounts (C) and size (D) in the brain regions in panels A and B. Representative images of immunofluorescence staining (anti-Iba1) of WT (E) and R6/2 (F) mouse brains with or without PROTAC **2′** treatment. (G) Quantification of inflammation level in panels E and F. Representative images of immunofluorescence staining (anti-DARPP32) of WT (H) and R6/2 (I) mouse brains with or without PROTAC **2′** treatment. (J) Quantification of DARPP32 level in panel H and I. All the statistical results were quantified by ImageJ and shown as mean ± SD. The region for statistical analysis was arbitrarily selected (mouse number = 3, each dot represents an independent mouse brain; the technical replicates are shown by the violin plot, the selected region for analysis = 6). Panel C was analyzed by a two-tailed Welch’s *t*-test due to the unequal variance (**P < 0.01). Panel D was analyzed by a two-tailed unpaired *t*-test. Panels G and J were analyzed by one-way ANOVA with Tukey’s posthoc test (*P < 0.05, **P < 0.01, ***P < 0.001).

Because mHTT accumulation is known to be associated with neuroinflammation in HD brain,^40^ we further investigated whether PROTAC **2′** modulates inflammatory responses. In particular, activated microglia, marked by Iba1 expression, are often elevated in the cortex of R6/2 mice.^41^ We next investigated whether PROTAC **2′** reduces neuroinflammation in the R6/2 mouse brain as a consequence of mHTT aggregate degradation and partially restores neuronal function. After confirming that PROTAC **2′** administration did not induce Iba1-positive microglial activation in wild-type mouse brain (Figure 7E,G), we observed a significant reduction in Iba1 signal in the cortical region of PROTAC **2′**-treated R6/2 mice compared to vehicle-treated controls (Figure 7F,G). In addition, PROTAC **2′** lowered the expression of CD68 (Figure S7A,B) and Galectin-3 (Figure S7C,D), which are expressed in Iba1-positive microglia, in R6/2 mouse brain. To further assess neuronal integrity in the R6/2 mouse brain after PROTAC treatment, we evaluated DARPP-32, which is highly enriched in medium spiny neurons of the striatum (Figure 7H,J).^42^ As shown in Figure 7I, PROTAC **2′** treatment increased the striatal DARPP-32 signal compared to the vehicle-treated controls (Figure 7J). Together, these findings indicate that PROTAC **2′** reduces mHTT aggregates while concurrently mitigating neuroinflammation and partially restoring neuronal function in the R6/2 HD mouse model.

## Discussion

Current disease-modifying strategies for HD primarily focus on reducing mHTT RNA levels through gene silencing and targeted protein degradation. Although recent advances in PROTACs have demonstrated promise in treating various neurodegenerative disease models,^14,43^ their application in HD remains in early stages, with limited *in vivo* data addressing long-term efficacy and safety. By contrast, RNA-based therapies such as ASOs and RNAi have progressed into clinical trials. ASOs directly bind to HTT mRNA and are capable of reducing mHTT expression throughout the central nervous system (CNS) via intrathecal injection.^44^ While preclinical models showed sustained HTT reduction and improved neuropathology,^45^ clinical studies revealed challenges, including the need for repeated invasive delivery and potential immune responses.^44^ Similarly, RNAi-based therapies, particularly adeno-associated virus (AAV)-delivered short hairpin RNAs, have demonstrated robust suppression of mHTT in animal models.^46^ However, concerns persist regarding irreversible gene silencing and immune activation from prolonged vector expression. Collectively, these limitations highlight the need for approaches that can reduce toxic mHTT species without permanently silencing wild-type HTT, ideally through noninvasive delivery routes that mitigate systemic toxicity and inflammation.

In this study, PROTAC **2′** was designed with distinct considerations in aggregate recognition and E3 ligase recruitment. Previous work has shown that benzothiazole-based PROTACs coupled with cIAP1 ligands can reduce mHTT levels in cells.^15^ However, cIAP1 is typically expressed at low levels in neurons^47^ and may be further downregulated under pathological stress in the CNS.^48^ In contrast, PROTAC **2′** recruits CRBN, an E3 ligase with more consistent and stable expression in neuronal tissues,^49,50^ to degrade mHTT aggregates at a relatively low treatment concentration in N2a. With respect to the aggregate-binding moiety, the PVA motif enabled selective degradation of mHTT aggregates while sparing normal HTT, thereby potentially reducing off-target effects and treatment-related side effects in Huntington’s disease. Importantly, the parent compound [^18^F]­AV-45, from which PVA is derived, has undergone extensive preclinical safety pharmacology and toxicology evaluation, and human PET imaging studies involving approximately 555 subjects have reported no serious adverse events.^51,52^ These findings indicate that high-affinity binding to amyloid aggregates, including nonspecific interactions with mHTT aggregates, does not necessarily result in overt cellular toxicity. Together, these observations support the feasibility of PROTAC-mediated degradation of mHTT in both *in vitro* and *in vivo* settings and suggest that PROTACs may serve as an alternative allele-selective strategy for reducing toxic mHTT species.

Beyond its degradation efficacy, reliable detection of PROTACs in brain tissue is essential for an *in vivo* evaluation. Initial extraction using standard methods such as the Bligh and Dyer protocol^53^ or acetonitrile-ethyl acetate extraction^54^ failed to yield detectable PROTAC **2′** in brain homogenates. Optimization of the extraction solvent ratio, particularly increasing the volume of the chloroform (organic) layer, allowed successful detection at concentrations as low as 1 ppb from 50 mg of brain tissue. This improved protocol may also benefit future pharmacological studies of hydrophobic compounds in complex matrices.

Although the large molecular size and high topological polar surface area of PROTACs have traditionally raised concerns about limited CNS penetration,^55^ emerging evidence suggests otherwise.^56,57^ For instance, the tau-targeting PROTAC C004019 (1035 Da) successfully crossed the BBB and reduced tau levels in mouse brain after subcutaneous injection.^28^ Likewise, PROTAC **2’** (614 Da) reached detectable concentrations in the brain (1.96 ppb) and conferred significant therapeutic benefits in the R6/2 HD model. These results challenge the predictive value of Lipinski’s Rule of Five^58^ for assessing CNS druggability and support the importance of empirical evaluation. The pronounced efficacy of PROTAC **2′** despite its low brain concentration, may be explained by the catalytic mechanism of PROTACs, which allows effective target degradation at substoichiometric levels. A previous study showed that a lower dose of tau-targeting PROTAC (3 mg/kg) outperformed a higher dose (20 mg/kg) in degrading tau *in vivo*.^28^ Additionally, the early onset BBB disruption observed in the R6/2 HD mice, beginning as early as 6 weeks of age,^59^ may impact the brain uptake of systemically administered compounds. Given that BBB impairment is common across many neurodegenerative conditions, such models may provide a more relevant context for evaluating large-molecule therapeutics like PROTACs.

Although PROTAC **2′** demonstrated clear brain penetration following subcutaneous administration, CNS exposure should be interpreted in the context of the dosing strategy and disease state. In wild-type mice, PROTAC **2′** reached low nanomolar concentrations in brain tissue, well below levels associated with cytotoxicity in vitro (Figure S1). The therapeutic studies employed a low-dose, continuous regimen designed to minimize peak exposure while maintaining sustained target engagement, which was well tolerated in wild-type animals. Blood–brain barrier integrity may be altered in neurodegenerative disease models such as R6/2 mice, potentially influencing brain exposure.^60^ Although direct quantitative comparisons between wild-type and R6/2 mice were not performed, the observed efficacy under this conservative dosing paradigm suggests that therapeutic benefits can be achieved without approaching toxic exposure levels. Future studies comparing brain pharmacokinetics across healthy and diseased models will be important for refining dosing strategies.

To further probe the mechanism of PROTAC **2′**, we evaluated its effect on mHTT oligomers, species increasingly recognized as cytotoxic contributors to HD pathology.^61^ Using fluorescence lifetime imaging microscopy-FRET (FLIM-FRET),^62^ we monitored HTT­(Q)_109_-eYFP (donor, Figure S8B) and HTT­(Q)_109_-mCherry (acceptor, Figure S8A) oligomerization in cells. By applying frequency domain fitting (details in Supporting Information and Methods), we resolved mHTT species into monomers, oligomers, and aggregates (Figure S8C). PROTAC **2′** significantly reduced the population of oligomeric intermediates (Figure S8D), while proteasome inhibition with MG132 increased oligomer levels. These results suggest that PROTAC **2′** effectively targets both soluble oligomers and insoluble aggregates for degradation. Notably, neuronal death in HD is not solely caused by mHTT aggregation but also by toxic gain-of-function effects of mHTT. These gain-of-function activities contribute to neuronal energy failure and oxidative stress through multiple mechanisms, including transcriptional dysregulation, mitochondrial dysfunction, proteasomal impairment, and defective autophagy.^63^ Therefore, addressing broader cellular dysfunctions may represent an additional important consideration for therapeutic intervention in HD.

As shown in the Figure 8, PROTAC **2′** is a brain-penetrant, amyloid-specific degrader that selectively eliminates both soluble mHTT oligomers and insoluble aggregates. In the R6/2 HD mouse model, it significantly improves motor function, reduces pathology, and extends survival with minimal toxicity. Its ability to act at low doses and avoid wild-type HTT degradation offers a noninvasive and selective therapeutic approach. These findings demonstrate the feasibility of PROTAC-mediated degradation of pathogenic protein species *in vivo* and highlight the potential of PROTAC-based strategies for treating HD and other neurodegenerative proteinopathies.

**Figure 8:**
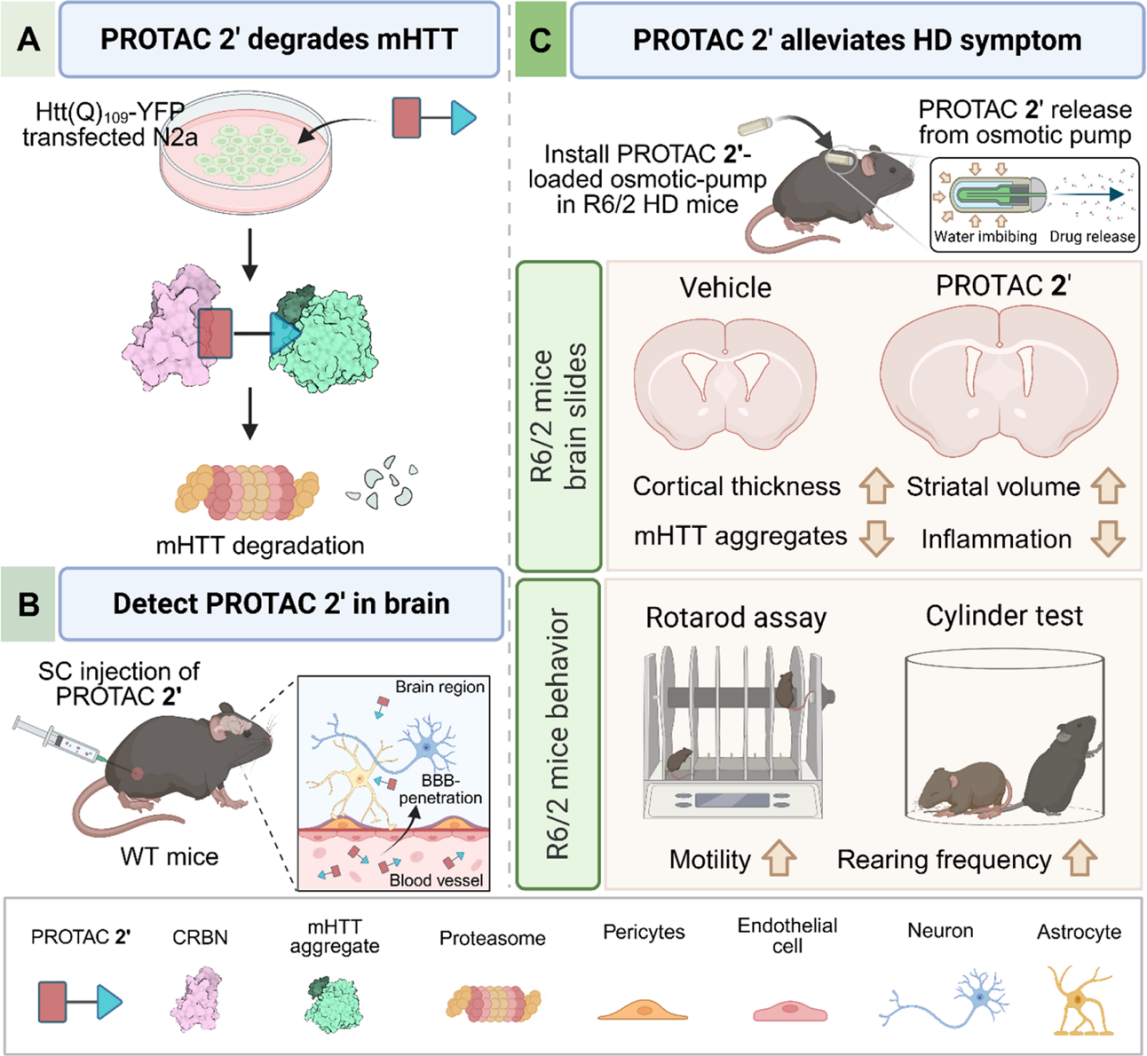
Graphical summary of PROTAC **2′**-mediated degradation of mHTT aggregates and its therapeutic effects in the mouse model. (A) Amyloid-specific PROTAC **2′** degrades mHTT aggregates via the proteasome in mHTT-expressing N2a cells. (B) Before evaluating the therapeutic effect of PROTAC **2′** in the R6/2 HD mouse model, we confirmed its ability to penetrate the BBB after SC injection in WT mice. (C) PROTAC **2′** was administered via osmotic pumps as a delivery route. After long-term, low-dose treatment, PROTAC **2′** significantly alleviated cortical and striatal atrophy, accompanied by decreased mHTT aggregates and inflammation in the R6/2 mouse brain. Furthermore, PROTAC **2′** improved motor coordination, increased body weight, and prolonged survival of R6/2 mice.

## Materials and Methods

### Plasmid Constructs

Both Htt­(Q)_25_-eYFP and Htt­(Q)_109_-eYFP were subcloned from pcDNA3.1-Htt-(Q)_25_-hrGFP and pcDNA3.1-Htt-(Q)_109_-hrGFP constructs into the pcDNA3 vector (Invitrogen Life Technologies). YFP fluorescence (eYFP) was subsequently fused at the C-terminus of the aforementioned constructs. The pEGFP-C3 and pcDNA3.1-mCherry plasmids were used as control plasmids for FLIM-FRET experiments.

### Cell Culture

N2a cells (mouse neuroblastoma) were kindly provided by Yijuang Chern (IBMS, Academia Sinica). Cells were cultured in Dulbecco’s modified Eagle’s medium (DMEM; Gibco) containing 2 × 10^–3^ M of glutamine, 10% heat-inactivated fetal bovine serum, and 100U mL^–1^ penicillin–streptomycin (Gibco) at 37 °C in a humidified incubator with 5% CO_2_.

### Insoluble HTT­(Q)_109_-eYFP Fractionation Form N2a Lysate

This method describes how to isolate RIPA-insoluble HTT­(Q)_109_-eYFP aggregates for SDS-PAGE analysis. Lysates from HTT­(Q)_109_-eYFP-expressing N2a cells were centrifuged at 70,000 *g* at 4 °C for 40 min (Optima MAX-XP Ultracentrifuge, Beckman Coulter). The supernatant (RIPA-soluble fraction) was collected, and the pellet (insoluble HTT­(Q)_109_-eYFP aggregates) was further washed twice with RIPA buffer by centrifugation at 70,000 *g* at 4 °C for 10 min. The final pellet was resuspended in 50 μL of 1% sarkosyl buffer (1 g of sarkosyl in 100 mL of 1 x PBS) via vigorous pipetting. Both fractions were then processed for Western blot analysis.

### AlamarBlue Reduction Assay

N2a cells were seeded in a 24-well plate at a density of 8 × 10^4^ cells/well and incubated overnight. Then, attached cells were transfected with the Htt­(Q)_109_-eYFP plasmid (1.1 μg) using TurboFect transfection reagent (Invitrogen) according to the manufacturer’s recommendations. After 2 h transfection, cells were further treated with various concentrations of PROTAC **2′** as indicated in the figures. After 48 h, AlamarBlue (Invitrogen) was added and incubated for 2–4 h. A 200 μL aliquot of conditioned medium was transferred to a 96-well plate, and fluorescence intensity (λ_ex_ = 560 nm, λ_em_ = 590 nm) was measured to assess cell viability.

### Confocal Microscopy

To assess the degradation ability of PROTAC candidates, N2a cells (2 × 10^5^) were seeded in 35 mm μ-Dish (ibidi, Martinsried, Germany) and transfected with Htt­(Q)_109_-eYFP plasmid (1.1 μg) using the Lipofectamine transfection reagent (Invitrogen). Two hours post-transfection, cells were treated with 1 μM PROTAC candidates or negative control and incubated for 22 h. Cells were fixed in 4% paraformaldehyde for 15 min and stored in 1× PBS. For proteasome inhibition studies, 1 μM MG132 was added 1 h prior to PROTAC treatment. Confocal images were captured using an FV3000 confocal laser scanning microscope (Olympus, Japan). The 440 nm laser was used for excitation of PROTAC **2′** with emission from the 405–495 nm bandpass filter. The 488 nm laser was used for excitation of eYFP with an emission from the 507–540 nm bandpass filter.

### Western Blot

For gel electrophoresis and blotting, all the necessary materials and procedures were described in a previous paper.^64^ Proteins were separated using 12% Tris-glycine SDS-PAGE. Proteins were transferred onto a PVDF membrane (Millipore). Blots were blocked with 5% bovine serum albumin (BSA, Sigma) in 0.1% PBST for at least 1 h. After blocking, blots were subjected to incubation with the primary antibodies PolyQ (1:1000, Merck Millipore, MAB1574), GFP (1:1000, Abcam, ab183734), GAPDH (1:10000, GeneTex, GTX627408), and γH2A.X (phosphor-Ser139) (1:1000, Merck Millipore, 05–636) in 2–5% BSA and incubated overnight at 4 °C on a shaker. After washing with 0.1% PBST, the blots were further incubated with HRP-labeled secondary antibodies [1:15000, anti-Rabbit (GeneTex, GTX213110-01), anti-Mouse (Jackson ImmunoResearch Laboratories, Inc., 115-035-003)] at room temperature for another 1 h. The blots were washed and developed with electrochemiluminescence (ECL, Millipore). The signals were visualized with the luminescence iBright FL1000 instrument (Invitrogen).

### Animals and PROTAC **2**′ Administration Methods

All animal experiments were approved by the Academia Sinica Institutional Animal Care and Use Committee (AS IACUC No. BioTReC-113-M-007). R6/2 transgenic mice ([B6CBA-Tg­(HDexon1)­62Gpb/1J] were generated using assisted reproductive technology (ART) at the National Laboratory Animal Center (NLAC). For ART, male R6/2 mice were obtained from the animal core at the Institute of Biomedical Sciences (IBMS), Academia Sinica), and female mice (B6CBAF1) were purchased from NLAC. Genotyping was conducted using PCR (primers listed in Supporting Information Table S1). Male R6/2 mice were housed in groups (no more than five mice per cage) with an enriched environment in a 12-h light/dark cycle and given water and food ad libitum in the AS core (Academia Sinica). The PROTAC **2′** was dissolved in 60% DMSO with 40% 1X PBS. For the subcutaneous injection, the dosage of PROTAC **2′** were prepared with 3 mg/kg/mouse in less than 50 μL liquid volume. For continuous delivery in therapeutic experiments, PROTAC **2′** was loaded into ALZET osmotic minipumps (model 2004, DURECT Corporation), which were preincubated overnight in sterile 1× PBS at 37 °C to ensure proper priming. Total PROTAC **2′** amount was calculated based on 25 g mouse weight, 0.25 μL/h pump rate, and 200 μL volume using the “Drug Concentration Calculator” provided by ALZET. Pumps were surgically implanted subcutaneously into the interscapular region under isoflurane anesthesia, following the manufacturer’s instructions. Mice were monitored daily for their health status and general behavior during the infusion period.

### PROTAC **2**′ Extraction from Mouse Brain

This method is developed for extracting the “hydrophobic” PROTAC **2′** from the mouse brain. PROTAC **2′** containing mouse brain was kept at −80 °C until extraction. Each 50 mg brain sample was homogenized in 42 μL of cold H_2_O and 200 μL of MeOH at 25 s^–1^ for 2 min (MM400, Retsch). Then, the homogenate was transferred to a new 15 mL tube, followed by the addition of 220 μL of cold H_2_O and 2 mL of cold CHCl_3_. After vortexing and rotating at 30 rpm for 30 min at 4 °C, samples were centrifuged at 13,000 rpm for 10 min.

After centrifugation, the chloroform layer (bottom phase) was collected and transferred to a new 15 mL tube (pierce the protein disk carefully). To freeze-dry the chloroform layer, a small volume of the layer (<500 μL, to avoid sample bumping or foaming during freeze-drying) was snap-frozen in liquid nitrogen and dried using a ScanVac freeze-dryer (LaboGene, Denmark). The temperature of the collector inside the freeze-dryer should be lower than the freezing point of the solvent. The freeze-drying process was repeated several times until all the chloroform layer was completely dry. The extracted PROTAC **2′** was kept at – 80 °C for storage. Before LC–MS/MS analysis, the PROTAC **2′** was resuspended by the proper solvent of CHCl_3_: DMSO: MeOH (1:1:1, v/v/v). For creating the calibration curve of PROTAC **2′** as a reference of the experimental group, PROTAC **2′** was directly spiked into 50 mg of mouse brain at various concentrations from 1 to 50 ppb.

### LC–MS/MS Analysis

The LC–MS/MS analysis was conducted using an LCMS-8045 triple quadrupole mass spectrometer (Shimadzu, Kyoto, Japan). Chromatographic separation was achieved on a Waters Acquity UPLC CSH C18 column (2.1 × 100 mm, 1.7 μm) equipped with a corresponding guard column (Waters Co., Milford, MA, USA), with the column temperature maintained at 40 °C.

The mobile phase consisted of solvent A (acetonitrile containing 0.1% (v/v) formic acid) and solvent B (water containing 0.1% (v/v) formic acid). The gradient elution program was as follows: 30% B for the initial 0.5 min, linearly increased to 85% B over 2 min, held at 85% B for 1 min, and then returned to 30% B over 4 min for re-equilibration. The flow rate was set at 0.30 mL/min, and the injection volume was 2 μL.

Data acquisition was performed in positive ionization mode with the following source parameters: interface temperature, 300 °C; desolvation line (DL) temperature, 250 °C; heat block temperature, 400 °C; nebulizing gas flow, 3 L/min; heating gas flow, 10 L/min; and drying gas flow, 10 L/min. Precursors for data-dependent acquisition were isolated within ±1 Th and subjected to collision-induced dissociation using optimized collision energies.

### Cortical Thickness and Striatal Volume Quantification

H&E staining was performed on 5 μm-thick brain sections from WT and R6/2 mice, with or without PROTAC **2′** treatment. The stained sections were scanned by using a high-resolution digital slide scanner (Aperio AT2, Leica Biosystems). Cortical thickness and striatal volume were assessed using Aperio ImageScope software (Leica Biosystems), which enabled precise visualization and annotation of the tissue morphology. The cortical thickness and striatal volume of the whole-brain images were viewed by using Aperio ImageScope software (Leica Biosystems), which allowed for precise examination and annotation of tissue morphology. Following established brain measurement protocols,^30^ cortical thickness were measured by drawing lines at positions 10, 11, and 12 of level 3 coronal brain sections (Figure S5A). The striatal volume was directly acquired from the above-mentioned software after circle selection of the striatal region (Figure S5A).

### Tissue Immunofluorescence Staining

The brain slides were deparaffinized with xylene (10 min, twice), followed by rehydration with a graded ethanol series (100%, 95%, 90%, and 70%; 5 min for each tank). After rehydration, heat-induced antigen retrieval was performed with a commercial kit (sigma, C9999) at 95–100 °C for 10 min. Brain sections were then permeabilized in 0.5% Triton X-100 diluted in 1 x PBS for 30 min. Nonspecific binding sites were blocked by incubating in 4% (w/v) BSA containing 0.1% Triton X-100 for 1 h at RT. For immunofluorescence staining, sections were incubated overnight at 4 °C with EM48 (1:250, Merck Millipore, MAB5374), IBA1 (1:250, Proteintech, 10904-1-ap), DARPP-32 (1:50, Cell Signaling, #2302), CD68 (1:100, Abcam, ab31630), and Galectin-3 B2C10 (1:200, Invitrogen, MA1-40229) primary antibodies. After O/N incubation, the sections were washed 3 times with PBS and subsequently incubated with Alexa-Fluor 488-conjugated secondary antibodies (Invitrogen) for 1 h at 37 °C. After washing three times in PBS, the sections were counterstained with DAPI (1:1000) for 10 min. Finally, samples were washed, dehydrated, and mounted onto slides using mounting medium (sigma, 06522). All of the slides were acquired with a CellVoyager CQ1 Benchtop High-Content Analysis System (Yokogawa, Japan).

### Rotarod Assay

Motor coordination (latency to fall) was assessed with a rotarod apparatus (LE8505 Rota Rod, Panlab) under the acceleration mode from 4 to 40 rpm over a 5 min period. All mice were tested twice per week. Each mouse was given three trials to run (with at least 30 min of rest between trials), and the highest latency to fall was recorded. Prior to testing, mice were trained to walk on the rotarod at a constant speed of 12 rpm for 2 min.

### Behavior Test

Motor neuron function was assessed with a cylinder assay, which monitors the rearing frequency of mice.^65^ Each mouse was placed in a clear glass cylinder (diameter, 15 cm; height, 27 cm) and recorded on video for 5 min at the age of 14 weeks. The rearing frequency was calculated by counting the times the mouse raised the forelimb above the shoulder height, and the two forelimbs were removed from the cylinder before next rearing.

### Liver and Kidney Function Tests

At the end of the experimental period, blood samples were collected from the mice via cardiac puncture. Samples were centrifuged at 3000 rpm for 10 min at 4 °C to isolate the serum, which was stored at −80 °C until analysis. Serum levels of aspartate aminotransferase (AST), alanine aminotransferase (ALT), blood urea nitrogen (BUN), and creatinine (CRE) were measured using a Hitachi 3100 automated biochemical analyzer (Hitachi High-Technologies Corporation, Tokyo, Japan) according to the manufacturer’s instructions.

### Statistical Analysis

Statistical comparison of multiple groups was conducted by either one-way ANOVA with Tukey posthoc test or two-way ANOVA with Bonferroni’s or Dunnett’s multiple comparison test, as appropriate. Comparison between the two groups was analyzed by two-tailed unpaired *t* tests. Significance was accepted at *p* < 0.05. All the statistical figures and analysis were generated by GraphPad Prism9 software.

## Supplementary Material







## Data Availability

The data generated by this study are available on request from the corresponding author.

## Notes

Synthetic schemes, synthetic procedures and characterization of compounds, ^1^H, ^13^C, and ^19^F NMR, absorption, fluorescence spectra, and HPLC diagrams (PDF)
Additional rotarod assay (MP4)
Cylinder assay (MP4)
