# Supplementary material for: PROTAC-Mediated Degradation of mHTT Aggregates Attenuates Neurotoxicity in Cellular and R6/2 Mouse Models of Huntington’s Disease
Source: J Am Chem Soc. 2026 Feb 19;148(8):8107–21. doi: 10.1021/jacs.5c14078 (PMC12964395; doi:10.1021/jacs.5c14078)
Supplement: Supplementary file 1 [file ja5c14078_si_001.pdf]

Supplementary Information

PROTAC-mediated degradation of mHTT aggregates attenuates neurotoxicity in cellular and R6/2 mouse models of Huntington's disease

Po-Chao Lu,[†,‡,∥,§] Yung-An Huang,[†,§] Niaz Wali,[†] Mei-Chun Tseng,[†] Ruei-Yu He,[†] Yijuang Chern,[††] Tzu-Tang Wei,[‡] Jiun-Jie Shie,[†,*] and Joseph Jen-Tse Huang,[†,∥,⊥,&,*]

**Contents**	**Pages**
Figure S1. The cytotoxicity test of different linker-length PVA-PROTAC molecules in N2a cells.	S2
Figure S2. **JJS0434** (PROTAC **2'** without POM), **JJS0435** (PROTAC **2'** without PVA), and compound **26** lose their degradation ability against mHTT aggregates.	S3
Figure S3. PROTAC **2'** colocalized with mHTT aggregates in the presence of MG132 in N2a cells.	S4
Figure S4. Quantification of PROTAC **2'** concentration in mouse brain using LC-MS/MS.	S5
Figure S5. The morphology of brain, liver, and kidney tissue section in WT mice after PROTAC **2'** administration.	S6
Figure S6. WT mice brain section showed weak and few EM48 antibody background signal.	S7
Figure S7. PRTOAC **2'** reduced inflammatory microglia cells in R6/2 mice brain.	S8
Figure S8. PROTAC **2'** could decrease the population of mHTT oligomeric intermediate via UPS.	S9
Supplementary Materials and Methods	S10-11
Supplementary Reference	S11
Supplementary Table 1	S11
Experimental Section for Organic Synthesis	S12
Synthetic Procedures and Product Characterization	S13
Scheme S1. Synthesis of various linkers and a E3-ligand for PROTAC degraders	S13-16
Scheme S2. Synthesis of PROTAC degraders (PROTAC **1'**–PROTAC **4'**)	S17-31
Scheme S3. Synthesis of control fragments (**JJS0434** and **JJS0435**) of PROTAC degrader	S31-33
^{1}H and ^{13}C NMR spectra of synthesized compounds	S34-68

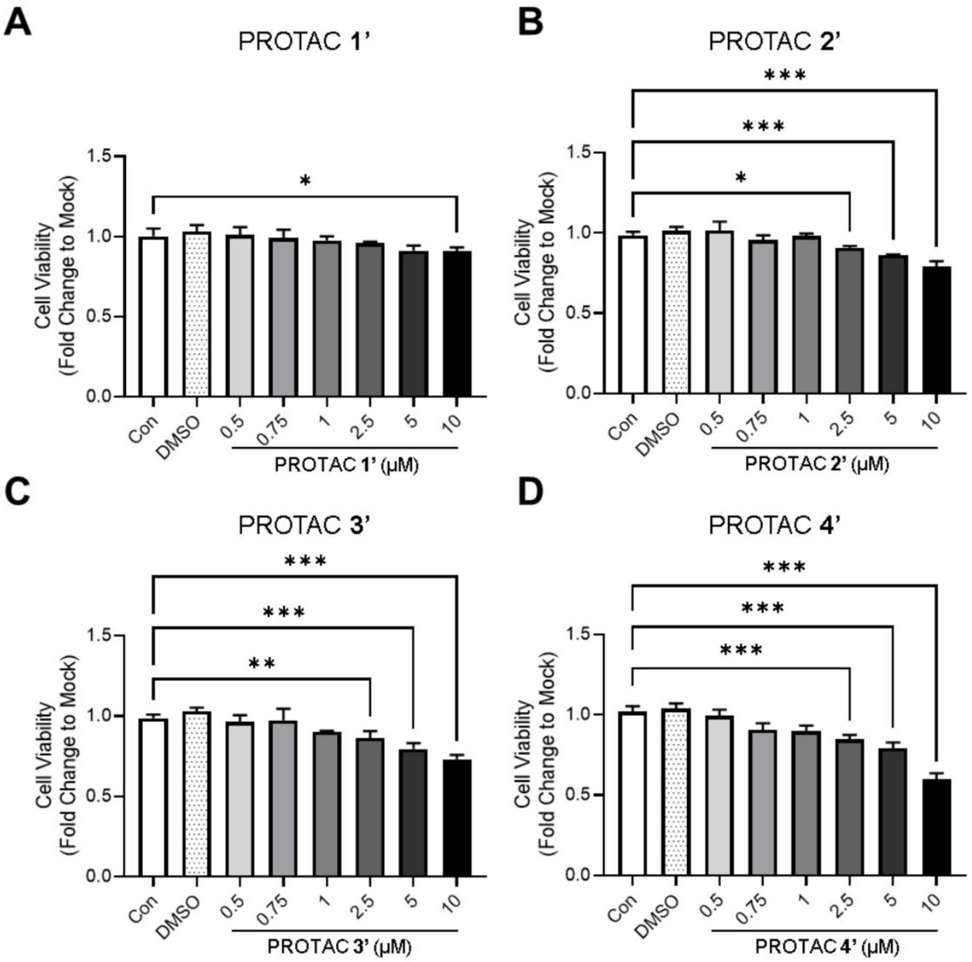

Figure S1. The cytotoxicity test of different linker-length PVA-PROTAC molecules in N2a cells. (A–D) AlamarBlue reduction assay of N2a cells treated with various concentrations of PROTAC **1’** (A), PROTAC **2’** (B), PROTAC **3’** (C), and PROTAC **4’** (D) individually (n = 3). All the statistical results are shown as mean ± SD. Panel A–D were analyzed by one-way ANOVA with Dunnett’s post-hoc test (*P < 0.05, **P < 0.01, ***P < 0.001).

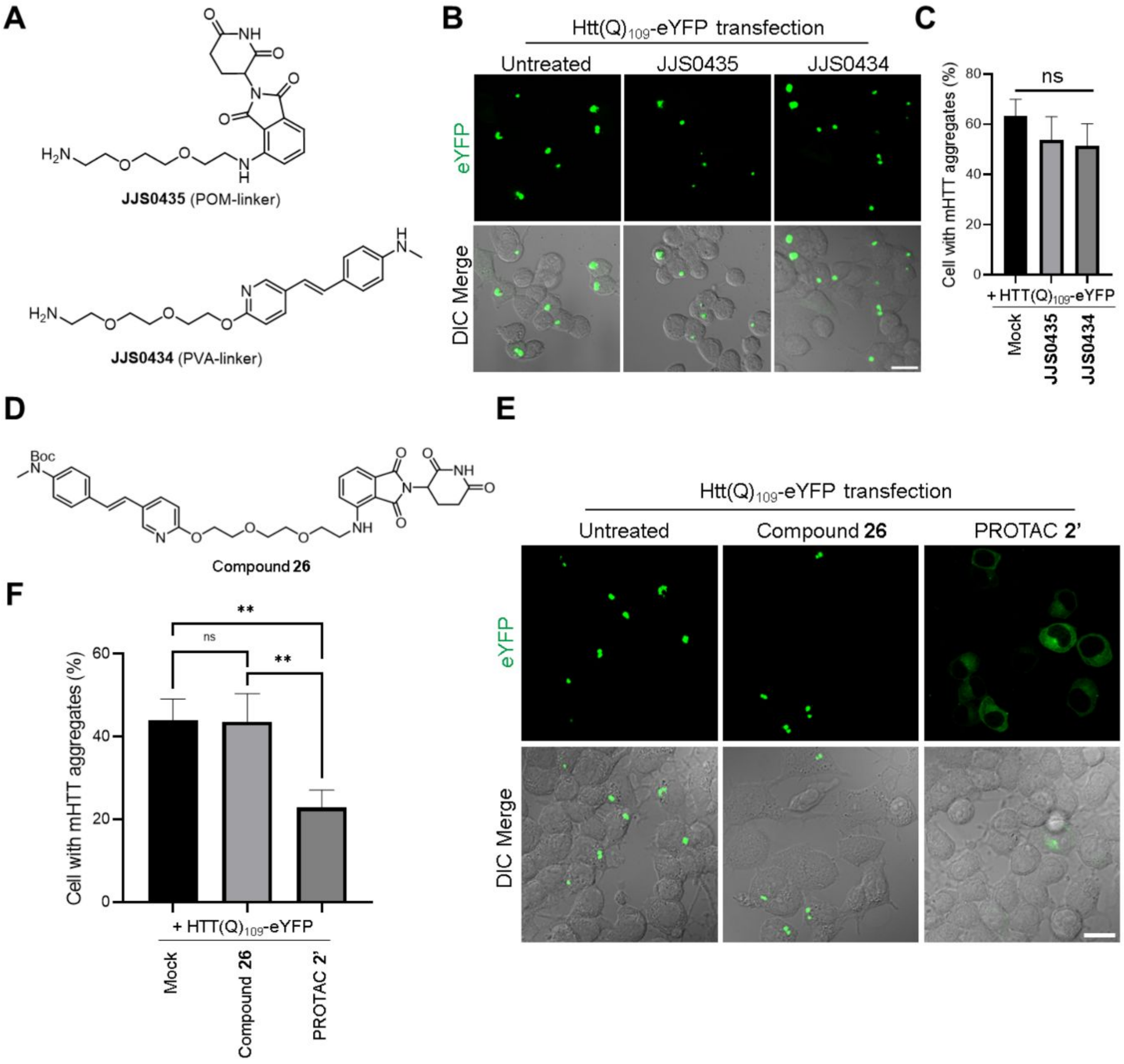

Figure S2. JJS0434 (PROTAC 2' without POM), JJS0435 (PROTAC 2' without PVA), and compound 26 lose their degradation ability against mHTT aggregates. (A) Molecular structure of **JJS0434** and **JJS0435** compounds. **(B)** Confocal images of HTT(Q)$_{109}$-eYFP-expressing N2a cells upon **JJS0434** (linker-PVA, 1 μM) or **JJS0435** (linker-POM, 1 μM) treatment. Scale bar = 20 μm. **(C)** Quantification of images in B (n = 3). **(D)** Molecular structure of compound **26**. **(E)** Confocal images of HTT(Q)$_{109}$-eYFP-expressing N2a cells upon compound **26** (1 μM) and PROTAC **2'** (1 μM) treatment. Scale bar = 20 μm. **(F)** Quantification of images in E (n = 3). The statistical results were calculated manually and shown as mean ± SD. Panel C and F were analyzed by one-way ANOVA with Tukey's post-hoc test (**P < 0.01).

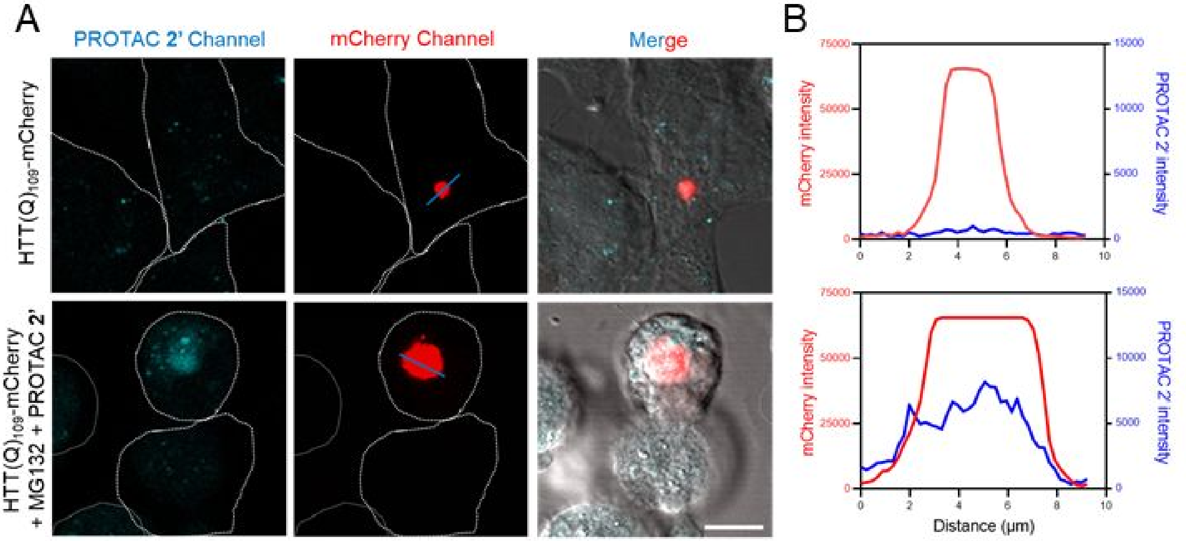

Figure S3. PROTAC 2' colocalized with mHTT aggregates in the presence of MG132 in N2a cells. (A) Confocal images of HTT(Q)$_{109}$-mCherry aggregates and PROTAC **2'** distribution in the N2a cells with or without 1 µM PROTAC **2'** (14 hr after MG132 pretreatment). To facilitate the visualization of the HTT(Q)$_{109}$-mCherry aggregates, MG132 (1 µM, 2 hr after transfection) was pretreated in PROTAC **2'** treated group. **(B)** Intensity profiles of PROTAC **2'** (blue) and HTT(Q)$_{109}$-mCherry (red) along the blue lines in panel A.

A B

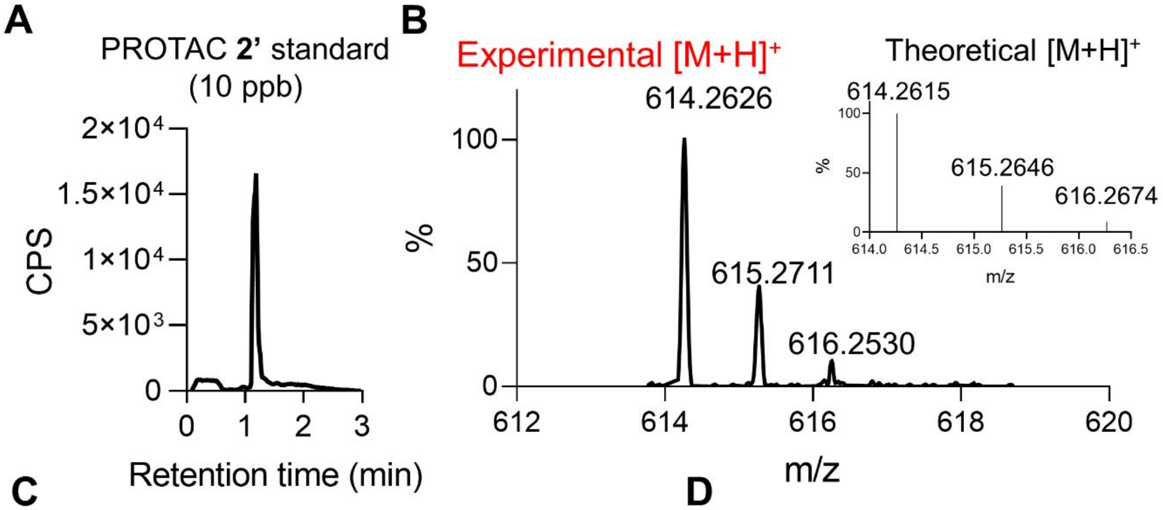

C

PROTAC **2’** extraction quality

	Recovery (%)	RSD (%)
Extraction Recovery (P/M)	85.9 ± 10.2	11.8
Matrix Effect (M/S)	102.8 ± 10.4	10.1
Process Efficiency (P/S)	87.7 ± 5.7	6.5

D

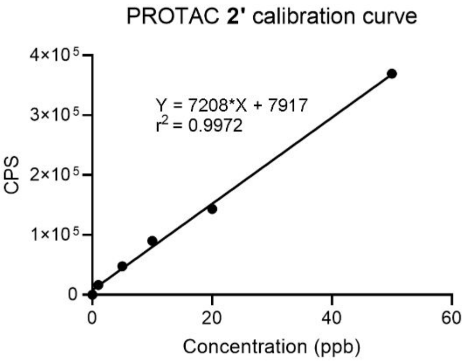

Figure S4. Quantification of PROTAC 2’ concentration in mouse brain using LC-MS/MS. **(A)** Retention time of 10 ppb PROTAC **2′** standard determined by LC-MS/MS. **(B)** Precursor ion mass spectrum of PROTAC **2′**, showing a mass accuracy of 1.8 ppm relative to the theoretical m/z value of 614.2615. **(C)** Evaluation of the extraction protocol, including recovery, matrix effect, and processing efficiency. Each value represents the mean of three replicates. S = standard solution; P = pre-extraction spiked sample; M = post-extraction spiked sample. **(D)** Calibration curve of PROTAC **2′** spiked into brain homogenates at concentrations ranging from 1 ppb to 50 ppb. Each data point represents the mean of three replicates.

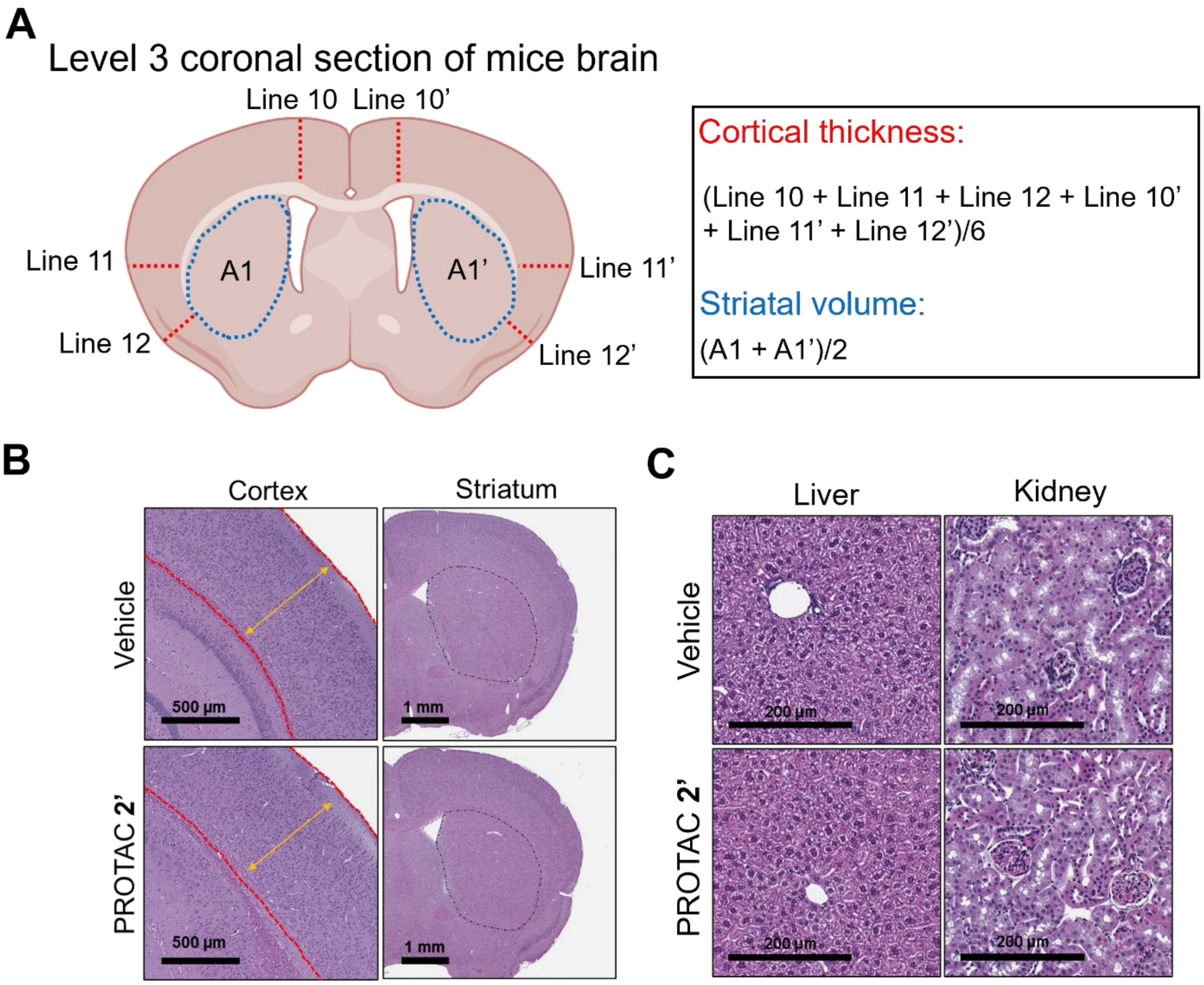

Figure S5. The morphology of brain, liver, and kidney tissue section in WT mice after PROTAC 2' administration. (A) Illustration of level 3 coronal section and measurement method (details in materials and methods). The reddish dotted line corresponding to the cortical thickness. The blue dotted line corresponding to the striatal volume. **(B)** The representative images of H&E staining of WT mice brain section with or without PROTAC **2'** (3 mg/kg) SC injection from the level 3 coronal section. The cortical (reddish dotted line) between line 10' to line 11' and striatal region (black dotted line) from A1' were shown accordingly. Yellow double arrow represents the thickness of cortex. **(C)** Representative images showed the morphology of liver and kidney tissue with or without PROTAC **2'** (3 mg/kg) SC injection.

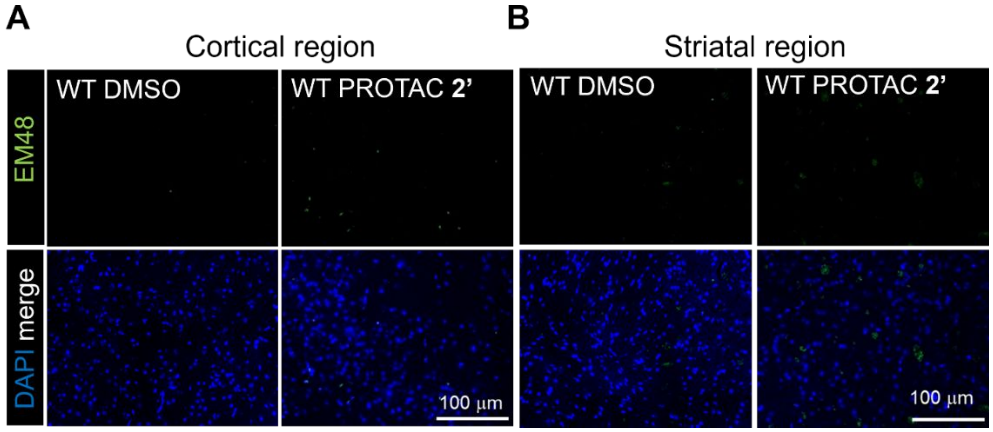

Figure S6. WT mice brain section showed weak and few EM48 antibody background signal. **(A–B)** The representative images of EM48 IF staining of B6CBA-WT mice cortex (A) and striatum (B) tissue section with or without PROTAC **2'** administration (3 mg/kg/week).

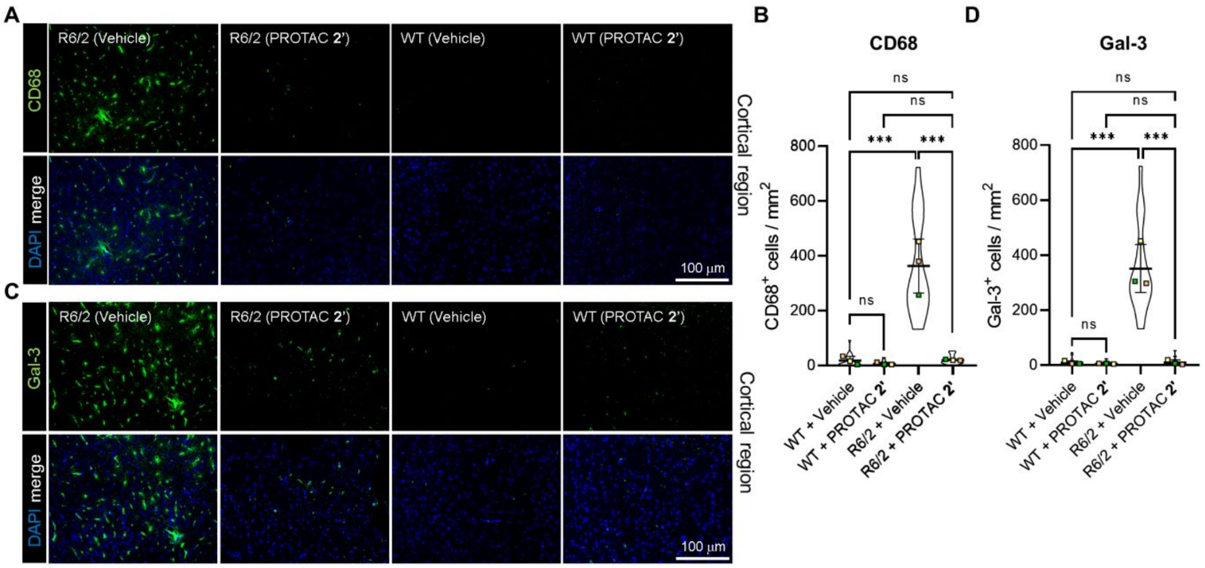

Figure S7. PRTOAC 2' reduced inflammatory microglia cells in R6/2 mice brain. (A) Representative images of immunofluorescence staining (anti-CD68) of WT and R6/2 mouse brains with or without PROTAC **2'** treatment. **(B)** Quantification of inflammation level in panel A. The region for statistical analysis was arbitrarily selected (N = 3, each dot represents an independent mouse brain; the technical replicates shown by violin plot, n = 6). **(C)** Representative images of immunofluorescence staining (anti-Gal-3) of WT and R6/2 mouse brains with or without PROTAC **2'** treatment. **(D)** Quantification of inflammation level in panel C. The region for statistical analysis was arbitrarily selected (N = 3, each dot represents an independent mouse brain; the technical replicates shown by violin plot, n = 6). All statistical results were quantified by ImageJ and shown as mean ± SD. Panel B and D was analyzed by one-way ANOVA with Tukey's post-hoc test (***$P < 0.001$).

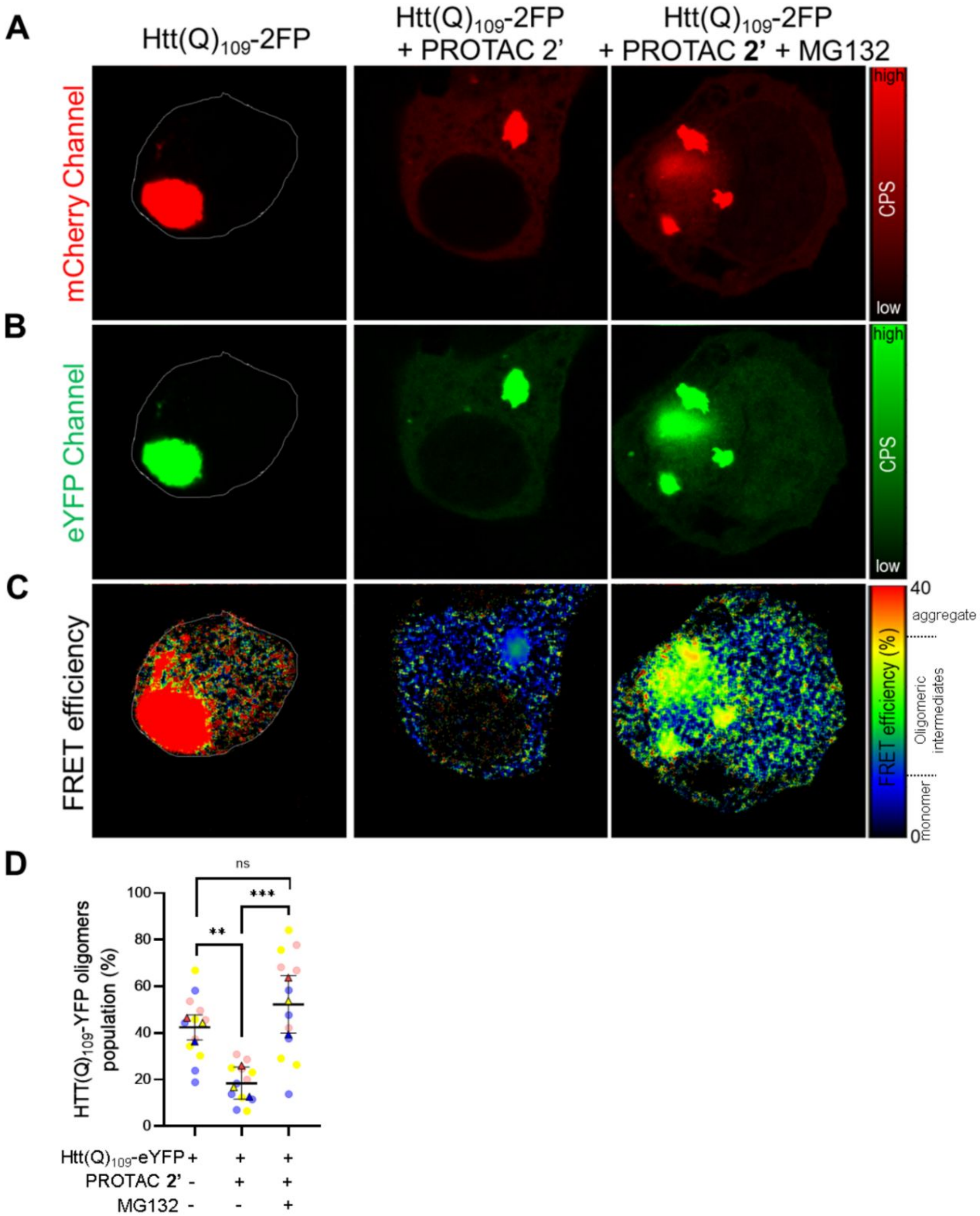

Figure S8. PROTAC 2' could decrease the population of mHTT oligomeric intermediate via UPS. (A-C) The representative images of HTT(Q)$_{109}$-mCherry- (A) and HTT(Q)$_{109}$-YFP- (B) expressing N2a cells treated with or without PROTAC **2'** (1 μM) and/or MG132 (1 μM). The images of FRET efficiency (C) revealed that the aggregation level of HTT(Q)$_{109}$ species according to the palette. **(D)** Quantification of the population of HTT(Q)$_{109}$ oligomeric intermediates (Each triangle represents an independent experiment, N = 3; each dot represents the technical replicates within each N, n = 4) in panel C. The statistical results were shown as mean ± SD. Panel D were analyzed by one-way ANOVA with Tukey's post-hoc test (**P < 0.01, ***P < 0.001).

Supplementary Materials and Methods

Frequency-domain fluorescence lifetime imaging. To study the degradation ability of PROTAC **2'** against mHTT oligomeric intermediates, we seeded 2 × 10^5 cells/dish of N2a cells in sterile 35 mm μ-Dish and transfected with both 0.55 μg Htt(Q)$_{109}$-eYFP and 0.55 μg mHtt(Q)$_{109}$-mCherry. After 2 h, PROTAC **2'** was delivered to the experimental group. MG132 (1 μM) was pre-treated 1 h before adding PROTAC **2'**. After 48 h incubation, the N2a cells were fixed (4% paraformaldehyde in 15 min and stored in 1× PBS buffer) and further analyzed by Q2 FastFLIM system (ISS Inc.). The expression level of both HTT(Q)$_{109}$-mCherry and HTT(Q)$_{109}$-eYFP were confirm in Figure S8A and S8B, respectively. The N2a cells were monitored and captured under oil-immersion objective observation [Nikon Plan Apo 100×/numerical aperture (NA) 1.4]. The HTT(Q)$_{109}$-eYFP and HTT(Q)$_{109}$-mCherry excitation sources came from a 488 nm (5 mW) and 561 mm (20 mW) sub-nanosecond modulated pulsed laser at the fundamental frequency of 20 MHz, which was controlled by ISS VistaVision software. The photon counts of eYFP were collected by GaAs photomultiplier tube (PMT) detector with EM1 filter (530/43 nm bandpass filter). The photon counts of mCherry were collected by GaAs photomultiplier tube (PMT) detector with EM1 filter (600/45 nm bandpass filter). To precisely obtain the lifetime value, the calibration of the system was operated by measuring fluorescein, a fluorophore with a single exponential lifetime around 4 ns in ddH_2O, every time before the measurement.

FLIM-FRET data analysis. The fitting method for FastFLIM images was detailed in the "Experimental Section" of a previous publication.[1] For the "frame" lifetime fitting model, the lifetime of each pixel in eYFP channel FLIM images (Figure S8B) were directly obtained by ISS Software VistaVision and subsequently transformed into E_{FRET} maps (Figure S8C). For the "highlighted-pixel" lifetime fitting model, we filtered out the aggregate species by thresholding photon counts of mHTT aggregates against high photon count pixels in Figure S8B. Then, we subsequently fitted the soluble mHTT (namely monomer and oligomer) with 2-exponential fitting to get the fraction of mHTT monomers and oligomeric intermediates. The fraction of HTT(Q)$_{109}$-eYFP oligomeric intermediates were obtained by fixing the HTT(Q)$_{109}$-eYFP monomers at 3.1 ns (the lifetime of eYFP). To fairly judge the E_{FRET} oligomeric intermediates, the N2a expressing HTT(Q)$_{109}$-2FP were arbitrarily selected. All of the lifetime values in this study were carefully

fitted in a reasonable range with the acceptable chi-square value (χ^2). HTT(Q)$_{109}$-2FP = either eYFP or mCherry fused mHTT.

Supplementary Reference

1. He, R. Y.; Lai, X. M.; Sun, C. S.; Kung, T. S.; Hong, J. Y.; Jheng, Y. S.; Liao, W. N.; Chen, J. K.; Liao, Y. F.; Tu, P. H.; Huang, J. J., Nanoscopic Insights of Amphiphilic Peptide against the Oligomer Assembly Process to Treat Huntington's Disease. *Adv Sci (Weinh)* **2020,** *7* (2), 1901165.

Supplementary Table 1

Primer sets for mouse genotyping

mHtt	5'-ACGGCCGCTCAGGTTCTG-3'<br>5'-AGGACTTGAGGGACTCGAAG-3'
CAG repeat	5'-CCGCTCAGGTTCTGCTTTTA-3'<br>5'-GGCTGAGGAAGCTGAGGAG-3'

Experimental Section for Organic Synthesis

Materials and Methods. All reagents were commercially available and used without further purification unless indicated otherwise. All solvents were anhydrous grade unless indicated otherwise. All nonaqueous reactions were carried out in an oven-dried glassware under a slight positive pressure of argon unless noted otherwise. Reactions were magnetically stirred and monitored by thin-layer chromatography on silica gel. Flash chromatography was performed on silica gel of 60–200 μm particle size. Yields are reported for spectroscopically pure compounds. Melting points were recorded on a Fargo MP-2D melting point apparatus and are not corrected. ^{1}H and ^{13}C NMR spectra were recorded on Bruker AV 600 (600 MHz), Bruker AV 500 (500 MHz), and Bruker AVIII 400 (400 MHz) spectrometers. Chemical shifts are given in δ values relative to tetramethylsilane (TMS, $\delta_H = 0$); coupling constants J are given in Hz. Internal standards were $CDCl_3$ (δ_H = 7.24), DMSO-d_6 (δ_H = 2.49) and CD_3OD (δ_H = 3.31) for ^{1}H NMR spectra. $CDCl_3$ (δ_C = 77.0), DMSO-d_6 (δ_C = 39.5) and CD_3OD (δ_C = 49.0) for ^{13}C NMR spectra. The splitting patterns are reported as s (singlet), d (doublet), t (triplet), q (quartet), m (multiplet), br (broad), and dd (double of doublets). IR spectra were recorded on a Thermo Nicolet iS5 FT-IR spectrometer. High-resolution electrospray ionization (ESI) mass spectra were recorded on a JMS-T100LP AccuTOF LC-plus 4G mass spectrometer. High-resolution FAB mass spectra were conducted on a JMS-700 double focusing magnetic sector mass spectrometer (JEOL, Tokyo, Japan) with a resolution of 8000 (5% valley definition). For FAB-mass spectra, the source accelerating voltage was operated 10kV with Xe gun, using 3-nitrobenzyl alcohol (NBA) as matrix. High-resolution EI mass spectra were conducted on a JMS-700 double focusing magnetic sector mass spectrometer (JEOL, Tokyo, Japan) with a resolution of 8000 (5% valley definition).

Synthetic Procedures and Product Characterization

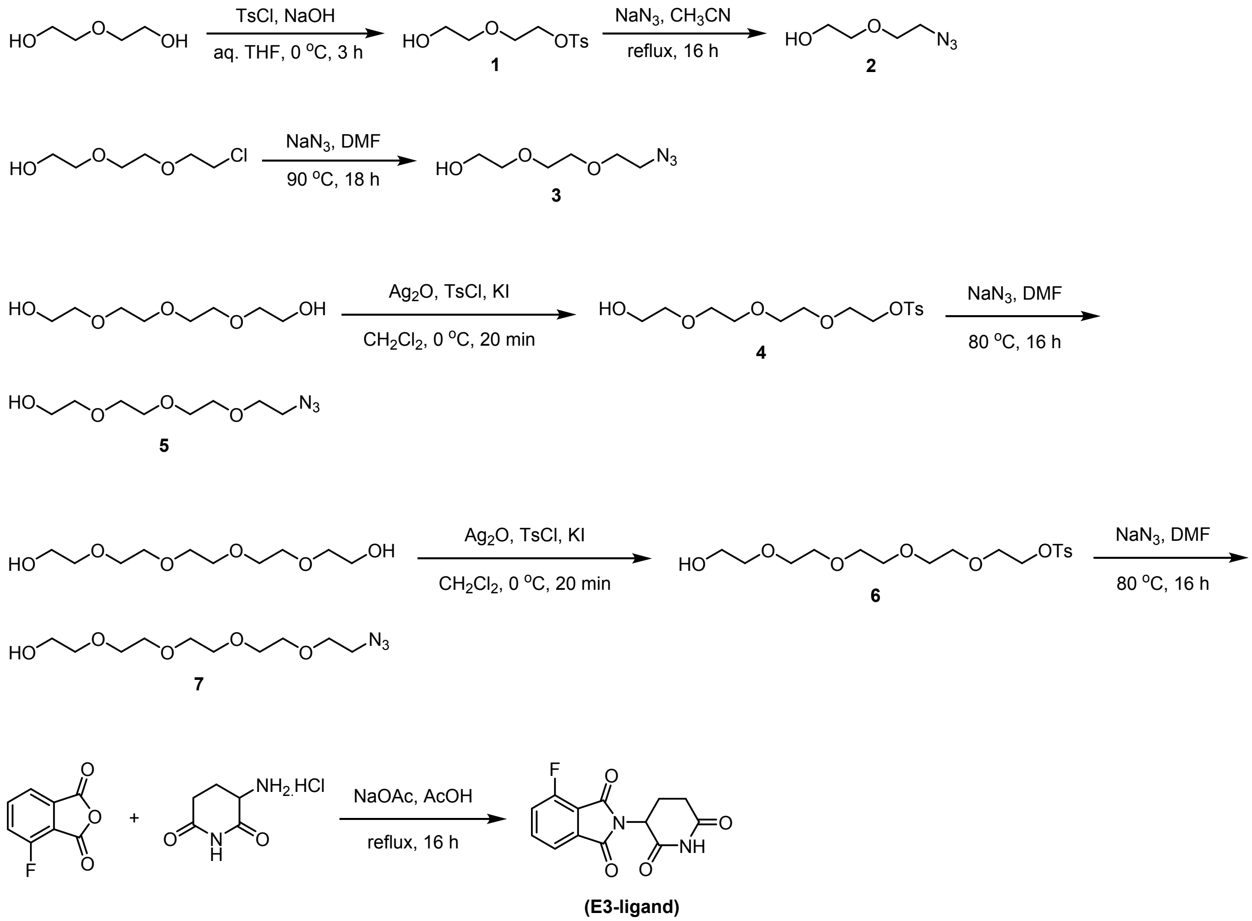

Scheme S1. Synthesis of various linkers and a E3-ligand for PROTAC degraders

2-(2-Azidoethoxy)ethan-1-ol (2)

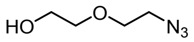

2

To a solution of diethylene glycol (19 mL, 200 mmol) in THF (15 mL) was added an aqueous solution of NaOH (1.28 g, 32 mmol) in H_2O (5 mL). The resulting mixture was slowly added a solution of TsCl (3.80 g, 20 mmol) in THF (5 mL) for 20 min at 0 °C. After stirring for 3 h at 0 °C, the reaction mixture was diluted with H_2O (80 mL) and extracted with CH_2Cl_2 (4 × 50 mL). The combined organic extracts were dried over $MgSO_4$, filtered, and concentrated to afford the crude compound **1**, which was used in next step without further purification.

To a solution of crude **1** in CH_3CN (50 mL) was added NaN_3 (2.08 g, 32 mmol). After refluxing for 16 h, the reaction mixture was diluted with H_2O (50 mL) and extracted with CH_2Cl_2 (3 × 100 mL). The combined organic extracts were dried over $MgSO_4$, filtered, and concentrated. The residue was purified by flash column chromatography on silica gel (25% acetone in hexane) to afford the desired product **2** (800 mg, 31% for two steps) as a colorless oil. $C_4H_9N_3O_2$; TLC (25% acetone in hexane) $R_f = 0.33$; ^1H NMR (500 MHz, $CDCl_3$) δ 3.73 (t, $J = 4.3$ Hz, 2H), 3.67 (t, $J = 4.8$ Hz, 2H), 3.59 (t, $J = 4.6$ Hz, 2H), 3.39 (t, $J = 4.9$ Hz, 2H); ^{13}C NMR (125 MHz, $CDCl_3$) δ 72.3, 70.0, 61.7, 50.7.

2-(2-(2-Azidoethoxy)ethoxy)ethan-1-ol (3)

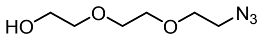

3

To a solution of 2-(2-(-2-chloroethoxy)ethoxy)ethanol (2.0 g, 11.8 mmol) in anhydrous DMF (20 mL) was added NaN_3 (1.15 g, 17.8 mmol). After stirring for 18 h at 90 °C, the reaction mixture was concentrated and the residue was diluted with H_2O, and extracted with CH_2Cl_2. The aqueous layer was extracted with CH_2Cl_2 (2 ×), The combined organic extracts were dried over $MgSO_4$, filtered, and concentrated to afford the desired product **3** (2.05 g, 99%) as a colorless oil. $C_6H_{13}N_3O_3$; TLC (15% acetone in hexane) $R_f = 0.26$; ^1H NMR (400 MHz, $CDCl_3$) δ 3.72 (t, $J = 4.4$ Hz, 2H), 3.68–3.64 (m, 6H), 3.60 (t, $J = 4.6$ Hz, 2H), 3.38 (t, $J = 5.0$ Hz, 2H); ^{13}C NMR (100 MHz, $CDCl_3$) δ 72.5, 70.7, 70.4, 70.0, 61.8, 50.7.

2-(2-(2-(2-Hydroxyethoxy)ethoxy)ethoxy)ethyl 4-methylbenzenesulfonate (4)

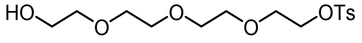

4

To a solution of tetraethylene glycol (3.10 g, 16 mmol) in anhydrous CH_2Cl_2 (160 mL) was added Ag_2O (5.56 g, 24 mmol), TsCl (3.35 g, 17.6 mmol) and KI (530 mg, 3.2 mmol) at 0 °C. After stirring for 20 min at 0 °C, the reaction mixture was filtered through a Celite pad and the residue was washed with EtOAc. The filtrate was concentrated and the residue was purified by flash column chromatography on silica gel (25% acetone in CH_2Cl_2) to afford the desired product **4** (4.06 g, 73%) as a colorless oil. $C_{15}H_{24}O_7S$; TLC (25% acetone in CH_2Cl_2) $R_f = 0.30$; ^1H NMR (500 MHz, $CDCl_3$) δ 7.77 (d, $J = 7.3$ Hz, 2H), 7.31 (d, $J = 7.8$ Hz, 2H), 4.14–4.12 (m, 2H), 3.69–3.65 (m, 4H), 3.63–3.60 (m, 4H), 3.58–3.56 (m, 6H), 2.41 (s, 3H); ^{13}C NMR (125 MHz, $CDCl_3$) δ 144.7, 132.9, 129.7, 127.9, 72.4, 70.6, 70.4, 70.2, 69.2, 68.6, 61.6, 21.6; ESI-HRMS calcd for $C_{15}H_{24}NaO_7S$ $[M + Na]^+$: 371.1134, found: *m/z* 371.1126.

2-(2-(2-(2-Azidoethoxy)ethoxy)ethoxy)ethan-1-ol (5)

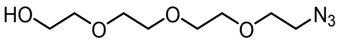

5

To a solution of **4** (3.80 g, 10.9 mmol) in anhydrous DMF (15 mL) was added NaN_3 (1.77 g, 27.2 mmol). After stirring for 16 h at 80 °C, the reaction mixture was diluted with H_2O (20 mL) and extracted with 5% methanol in CH_2Cl_2 (3 × 20 mL). The combined organic extracts were dried over $MgSO_4$, filtered and concentrated to afford the desired product **5** (2.36 g, 99%) as a colorless oil. $C_8H_{17}N_3O_4$; TLC (25% acetone in CH_2Cl_2) $R_f = 0.26$; ^1H NMR (500 MHz, $CDCl_3$) δ 3.70–3.67 (m, 2H), 3.65–3.62 (m, 10H), 3.59–3.56 (m, 2H), 3.37–3.34 (m, 2H), 2.48 (br s, 1H); ^{13}C NMR (125 MHz, $CDCl_3$) δ 72.4, 70.6 (2 ×), 70.5, 70.2, 69.9, 61.6, 50.6; ESI-HRMS calcd for $C_8H_{17}N_3NaO_4$ $[M + Na]^+$: 242.1111, found: *m/z* 242.1110.

14-Hydroxy-3,6,9,12-tetraoxatetradecyl 4-methylbenzenesulfonate (6)

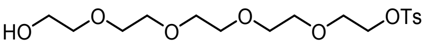

6

To a solution of pentaethylene glycol (3.81 g, 16 mmol) in anhydrous CH_2Cl_2 (160 mL) was added Ag_2O (5.56 g, 24 mmol), TsCl (3.35 g, 17.6 mmol) and KI (530 mg, 3.2 mmol) at 0 °C. After stirring for 20 min at 0 °C, the reaction mixture was filtered through a Celite pad and the residue was washed with EtOAc. The filtrate was concentrated and the residue was purified by flash column chromatography on silica gel (25% acetone in CH_2Cl_2) to afford the desired product **6** (4.39 g, 70%) as a colorless oil. $C_{17}H_{28}O_8S$; TLC (25% acetone in CH_2Cl_2) $R_f = 0.30$; ^1H NMR (400 MHz, $CDCl_3$) δ 7.77 (d, $J = 8.3$ Hz, 2H), 7.31 (d, $J = 8.1$ Hz, 2H), 4.13 (t, $J = 4.7$ Hz, 2H), 3.70–3.65 (m, 4H), 3.63–3.58 (m, 10H), 3.57–3.56 (m, 4H), 2.42 (s, 3H); ^{13}C NMR (100 MHz, $CDCl_3$) δ 144.7, 132.9, 129.8, 127.9, 72.5, 70.7, 70.5 (2 ×), 70.4 (2 ×), 70.2, 69.2, 68.6, 61.7, 21.6.

14-Azido-3,6,9,12-tetraoxatetradecan-1-ol (7)

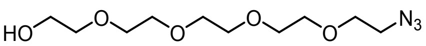

7

To a solution of compound **6** (1.96 g, 5 mmol) in anhydrous DMF (15 mL) was added NaN_3 (810 mg, 12.5 mmol). After stirring for 16 h at 80 °C, the reaction mixture was diluted with H_2O (20 mL) and extracted with 5% methanol in CH_2Cl_2 (3 × 20 mL). The combined organic extracts were dried over $MgSO_4$, filtered, and concentrated to afford the desired product **7** (1.30 g, 99%) as a colorless oil, which was used in next step without further purification. $C_{10}H_{21}N_3O_5$; TLC (25% acetone in CH_2Cl_2) $R_f = 0.26$; ^1H NMR (500 MHz, $CDCl_3$) δ 3.70–3.68 (m, 2H), 3.65–3.62 (m, 14H), 3.59–3.56 (m, 2H), 3.36–3.35 (m, 2H); ^{13}C NMR (125 MHz, $CDCl_3$) δ 72.5, 70.6, 70.5 (3

×), 70.2 (2 ×), 69.9, 61.7, 50.6; ESI-HRMS calcd for $C_{10}H_{21}N_3NaO_5$ $[M + Na]^+$: 286.1373, found: *m/z* 286.1376.

E3-ligand

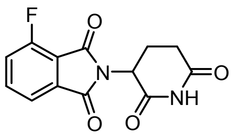

(E3-ligand)

To a solution of 3-fluoropthalic anhydride (5.98 g, 36 mmol) and 3-aminopiperidine-2,6-dione hydrochloride (5.92 g, 36 mmol) in HOAc (450 mL) was added NaOAc (3.54 g, 43.2 mmol). After refluxing for 16 h, the reaction mixture was concentrated, diluted with EtOAc, washed with water (3 × 100 mL). The aqueous layers extracted with EtOAc (4 × 100 mL) and the combined organic extracts were dried over $MgSO_4$, filtered and concentrated. The residue was purified by flash column chromatography on silica gel (2% CH_3OH in CH_2Cl_2) to afford the desired product **E3-ligand** (8.94 g, 90%) as a white solid. $C_{13}H_9FN_2O_4$; mp 289–290 °C; TLC (2% CH_3OH in CH_2Cl_2) $R_f = 0.33$; ^1H NMR (500 MHz, DMSO-d_6) δ 11.13 (s, 1H), 7.93 (ddd, J = 12.0, 7.5, 4.5 Hz, 1H), 7.77 (d, J = 7.3 Hz, 1H), 7.72 (t, J = 8.9 Hz, 1H), 5.14 (dd, J = 12.9, 5.5 Hz, 1H), 2.91–2.84 (m, 1H), 2.62–2.51 (m, 2H), 2.08–2.03 (m, 1H); ^{13}C NMR (125 MHz, DMSO-d_6) δ 172.7, 169.6, 166.0, 163.9, 157.8 (d, J_{C-F} = 260.9 Hz), 138.0 (d, J_{C-F} = 8.1 Hz), 133.4, 123.0 (d, J_{C-F} = 19.2 Hz), 120.0, 117.0 (d, J_{C-F} = 12.1 Hz), 49.0, 30.8, 21.8; FAB-HRMS calcd for $C_{13}H_{10}FN_2O_4$ $[M + H]^+$: 277.0625, found: *m/z* 277.0629.

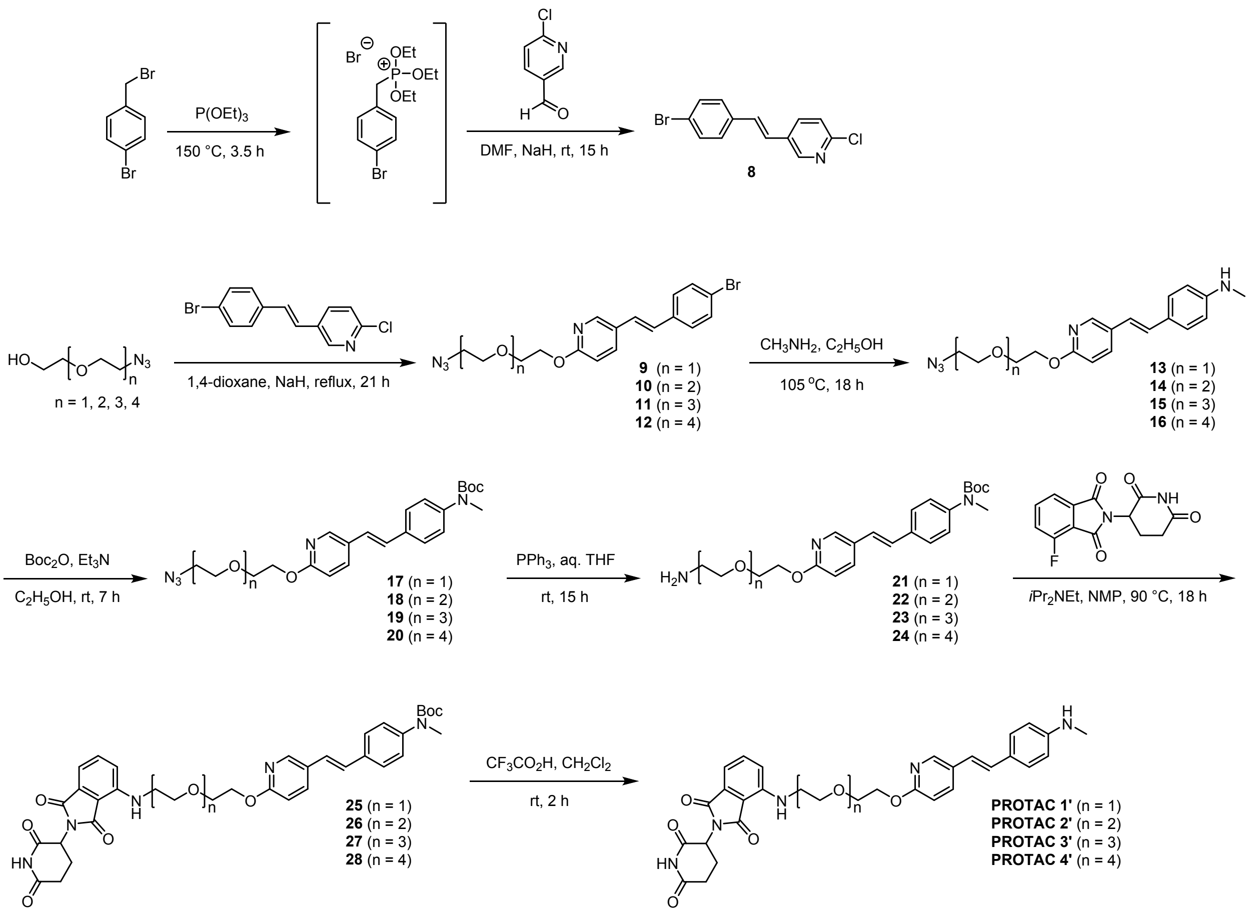

Scheme S2. Synthesis of PROTAC degraders (**PROTAC 1'–PROTAC 4'**)

(*E*)-5-(4-Bromostyryl)-2-chloropyridine (8)

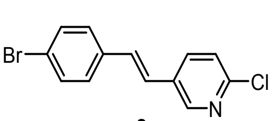

8

A solution of 4-bromobenzyl bromide (5 g, 20 mmol) in $P(OEt)_3$ (4.11 mL, 24 mmol) was stirred at 150 °C for 3.5 h. The resulting mixture was dissolved in anhydrous DMF (50 mL) and added NaH (710 mg, 30 mmol) to the reaction mixture. After stirring for 30 min at room temperature, the reaction mixture was slowly added a solution of 6-chloronicotin-3-carbaldehyde (3.40 g, 24 mmol) in anhydrous DMF (10 mL) at 0 °C. After stirring for 15 h at room temperature, the reaction mixture was diluted with EtOAc and washed with H_2O (2 × 25 mL). The aqueous layer was extracted with EtOAc (× 2) and the combined organic extracts were dried over $MgSO_4$, filtered and concentrated. The residue was purified by flash column chromatography on silica gel (40% EtOAc in hexane) to afford the desired product **8** (5.58 g, 94%) as a white solid. $C_{13}H_9BrClN$; mp 157–158 °C; TLC (30% EtOAc in hexane) R_f = 0.40; ^1H NMR (500 MHz, $CDCl_3$) δ 8.44 (d, *J* =

2.4 Hz, 1H), 7.77 (dd, J = 8.3, 2.5 Hz, 1H), 7.47 (d, J = 8.6 Hz, 2H), 7.35 (d, J = 8.6 Hz, 2H), 7.29 (d, J = 8.3 Hz, 1H), 7.03 (d, J = 16.2 Hz, 1H), 6.98 (d, J = 16.2 Hz, 1H); ^{13}C NMR (125 MHz, $CDCl_3$) δ 150.1, 148.1, 135.3, 135.2, 131.9, 131.7, 130.2, 128.1, 124.2, 124.0, 122.3; FAB-HRMS calcd for $C_{13}H_{10}BrClN$ [M + H]$^+$: 295.9668, found: *m/z* 295.9658.

General synthetic procedure and product characterization of compounds (9–12)

To a solution of azido-linker in anhydrous 1,4-dioxane was added NaH (2 equiv). After stirring for 1 h at room temperature, the resulting mixture was added compound **8** (1.5 equiv). After refluxing for 21 h, the reaction mixture was diluted with EtOAc and washed with H_2O (2 × 25 mL). The aqueous layer was extracted with EtOAc (2 ×) and the combined organic extracts were dried over $MgSO_4$, filtered and concentrated. The residue was purified by flash column chromatography on silica gel to afford the desired product (**9–12**).

(*E*)-2-(2-(2-Azidoethoxy)ethoxy)-5-(4-bromostyryl)pyridine (9)

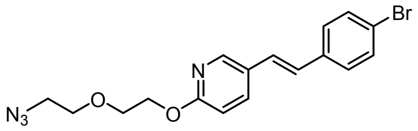

9

Compound **9** was prepared from a solution of azido-linker **2** (700 mg, 5.33 mmol), NaH (427 mg, 10.6 mmol) and compound **8** (2.36 g, 8 mmol) in anhydrous 1,4-dioxane (26 mL). The crude residue was purified by flash column chromatography on silica gel (30% EtOAc in hexane) to afford the desired product **9** (1.47 g, 71%) as a white solid. $C_{17}H_{17}BrN_4O_2$; mp 68–69 °C; TLC (40% EtOAc in hexane) R_f = 0.39; ^{1}H NMR (400 MHz, $CDCl_3$) δ 8.14 (d, J = 2.3 Hz, 1H), 7.74 (dd, J = 8.7, 2.5 Hz, 1H), 7.43 (d, J = 8.5 Hz, 2H), 7.30 (d, J = 8.6 Hz, 2H), 6.96 (d, J = 16.2 Hz, 1H), 6.86 (d, J = 16.2 Hz, 1H), 6.77 (d, J = 8.7 Hz, 1H), 4.48 (t, J = 4.7 Hz, 2H), 3.84 (t, J = 4.7 Hz, 2H), 3.70 (t, J = 4.9 Hz, 2H), 3.38 (t, J = 5.2 Hz, 2H); ^{13}C NMR (100 MHz, $CDCl_3$) δ 163.0, 145.7, 135.9, 135.3, 131.7 (2 ×), 127.7 (2 ×), 126.6, 126.3, 125.3, 121.2, 111.4, 69.9, 69.6, 65.1, 50.6; ESI-HRMS calcd for $C_{17}H_{18}BrN_4O_2$ [M + H]$^+$: 389.0607, found: *m/z* 389.0605.

(*E*)-2-(2-(2-(2-Azidoethoxy)ethoxy)ethoxy)-5-(4-bromostyryl)pyridine (10)

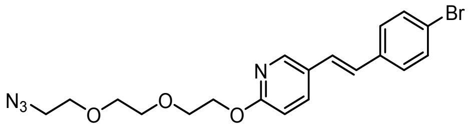

10

Compound **10** was prepared from a solution of azido-linker **3** (3.97 g, 13.5 mmol), NaH (719 mg, 18 mmol) and compound **8** (3.97 g, 13.5 mmol) in anhydrous 1,4-dioxane (66 mL). The crude residue was purified by flash column chromatography on silica gel (35% EtOAc in hexane) to afford the desired product **10** (2.88 g, 74%) as a white solid. $C_{19}H_{21}BrN_4O_3$; mp 48–49 °C; TLC (40% EtOAc in hexane) R_f = 0.36; ^{1}H NMR (500 MHz, $CDCl_3$) δ 8.16 (d, *J* = 2.2 Hz, 1H), 7.76 (dd, *J* = 8.6, 2.4 Hz, 1H), 7.44 (d, *J* = 8.4 Hz, 2H), 7.32 (d, *J* = 8.4 Hz, 2H), 6.98 (d, *J* = 16.3 Hz, 1H), 6.88 (d, *J* = 16.3 Hz, 1H), 6.78 (d, *J* = 8.6 Hz, 1H), 4.48 (td, *J* = 4.7, 3.4 Hz, 2H), 3.85 (td, *J* = 4.7, 3.4 Hz, 2H), 3.71–3.70 (m, 2H), 3.67–3.64 (m, 4H), 3.36 (t, *J* = 5.2 Hz, 2H); ^{13}C NMR (125 MHz, $CDCl_3$) δ 163.1, 145.5, 135.9, 135.5, 131.7 (2 ×), 127.7 (2 ×), 126.8, 126.4, 125.3, 121.3, 111.5, 70.7 (2 ×), 70.0, 69.7, 69.5, 50.6; ESI-HRMS calcd for $C_{19}H_{21}BrN_4NaO_3$ $[M + Na]^+$: 455.0689, found: *m/z* 455.0695.

(*E*)-2-(2-(2-(2-(2-Azidoethoxy)ethoxy)ethoxy)ethoxy)-5-(4-bromostyryl)pyridine (11)

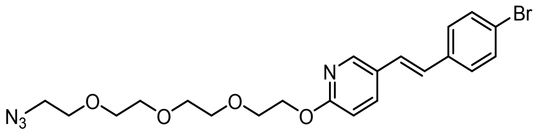

11

Compound **11** was prepared from a solution of azido-linker **5** (440 mg, 2 mmol), NaH (160 mg, 4 mmol) and compound **8** (883 mg, 3 mmol) in anhydrous 1,4-dioxane (10 mL). The crude residue was purified by flash column chromatography on silica gel (50% EtOAc in hexane) to afford the desired product **11** (705 mg, 74%) as a white solid. $C_{21}H_{25}BrN_4O_4$; mp 72–73 °C; TLC (50% EtOAc in hexane) R_f = 0.33; ^{1}H NMR (400 MHz, $CDCl_3$) δ 8.15 (d, *J* = 2.4 Hz, 1H), 7.75 (dd, *J* = 8.6, 2.4 Hz, 1H), 7.44 (d, *J* = 8.5 Hz, 2H), 7.31 (d, *J* = 8.5 Hz, 2H), 6.98 (d, *J* = 16.3 Hz, 1H), 6.88 (d, *J* = 16.3 Hz, 1H), 6.77 (d, *J* = 8.6 Hz, 1H), 4.47 (t, *J* = 4.7 Hz, 2H), 3.83 (t, *J* = 4.8 Hz, 2H), 3.71–3.61 (m, 10H), 3.35 (t, *J* = 5.2 Hz, 2H); ^{13}C NMR (100 MHz, $CDCl_3$) δ 163.2, 145.7, 136.0, 135.4, 131.7 (2 ×), 127.7 (2 ×), 126.7, 126.3, 125.4, 121.2, 111.4, 70.6 (4 ×), 70.0, 69.6, 65.3, 50.6; ESI-HRMS calcd for $C_{21}H_{25}BrN_4NaO_4$ $[M + Na]^+$: 499.0951, found: *m/z* 499.0957.

(*E*)-2-((14-Azido-3,6,9,12-tetraoxatetradecyl)oxy)-5-(4-bromostyryl)pyridine (12)

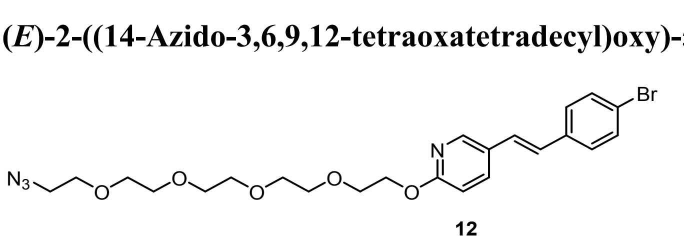

12

Compound **12** was prepared from a solution of azido-linker **7** (821 mg, 3.12 mmol), NaH (250 mg, 6.24 mmol) and compound **8** (1.83 g, 6.24 mmol) in anhydrous 1,4-dioxane (16 mL). The crude residue was purified by flash column chromatography on silica gel (50% EtOAc in hexane) to

afford the desired product **12** (1.12 g, 69%) as a white solid. $C_{23}H_{29}BrN_4O_5$; mp 44–45 °C; TLC (50% EtOAc in hexane) R_f = 0.31; ^1H NMR (400 MHz, $CDCl_3$) δ 8.15 (d, J = 2.3 Hz, 1H), 7.75 (dd, J = 8.7, 2.4 Hz, 1H), 7.44 (d, J = 8.6 Hz, 2H), 7.32 (d, J = 8.5 Hz, 2H), 6.98 (d, J = 16.3 Hz, 1H), 6.88 (d, J = 16.3 Hz, 1H), 6.77 (d, J = 6.5 Hz, 1H), 4.47 (t, J = 4.6 Hz, 2H), 3.83 (t, J = 4.8 Hz, 2H), 3.70–3.63 (m, 14H), 3.35 (t, J = 5.1 Hz, 2H); ^{13}C NMR (100 MHz, $CDCl_3$) δ 163.2, 145.7, 136.0, 135.4, 131.7 (2 ×), 127.7 (2 ×), 126.7, 126.3, 125.4, 121.3, 111.5, 70.6 (6 ×), 69.9, 69.6, 65.3, 50.6; ESI-HRMS calcd for $C_{23}H_{29}BrN_4NaO_5$ $[M + Na]^+$: 543.1213, found: *m/z* 543.1205.

General synthetic procedure and product characterization of compounds (13–16)

To a solution of compound (**9**–**12**) in ethanol and 40% aqueous CH_3NH_2 was added copper (0.1 equiv) in a sealed tube. After stirring for 18 h at 105 °C, the reaction mixture was diluted with EtOAc and washed with H_2O. The aqueous layer was extracted with EtOAc (2 ×) and the combined organic extracts were dried over $MgSO_4$, filtered and concentrated. The residue was purified by flash column chromatography on silica gel to afford the desired product (**13**–**16**).

(*E*)-4-(2-(6-(2-(2-Azidoethoxy)ethoxy)pyridin-3-yl)vinyl)-*N*-methylaniline (13)

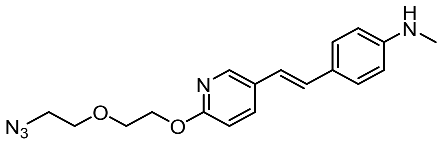

13

Compound **13** was prepared from compound **9** (195 mg, 0.5 mmol) and copper (3.2 mg, 0.05 mmol) in ethanol (1 mL) and 40% aqueous CH_3NH_2 (0.6 mL, 6.6 mmol). The crude residue was purified by flash column chromatography on silica gel (30% EtOAc in hexane) to afford the desired product **13** (118 mg, 70%) as a yellowish solid. $C_{18}H_{21}N_5O_2$; mp 64–65 °C; TLC (30% EtOAc in hexane) R_f = 0.32; ^1H NMR (500 MHz, $CDCl_3$) δ 8.12 (d, J = 2.3 Hz, 1H), 7.74 (dd, J = 8.7, 2.5 Hz, 1H), 7.32 (d, J = 8.5 Hz, 2H), 6.88 (d, J = 16.2 Hz, 1H), 6.79 (d, J = 16.2 Hz, 1H), 6.5 (d, J = 8.6 Hz, 1H), 6.58 (d, J = 8.3 Hz, 2H), 4.47 (t, J = 4.7 Hz, 2H), 3.84 (t, J = 4.7 Hz, 2H), 3.71 (t, J = 4.9 Hz, 2H), 3.39 (t, J = 5.2 Hz, 2H), 2.83 (s, 3H); ^{13}C NMR (125 MHz, $CDCl_3$) δ 162.4, 148.7, 144.8, 135.0, 128.2, 127.5 (3 ×), 126.5, 120.3, 112.5, 111.2 (2 ×), 69.9, 69.7, 65.0, 50.6, 30.7; ESI-HRMS calcd for $C_{18}H_{22}N_5O_2$ $[M + H]^+$: 340.1768, found: *m/z* 340.1760.

(*E*)-4-(2-(6-(2-(2-(2-Azidoethoxy)ethoxy)ethoxy)pyridin-3-yl)vinyl)-*N*-methylaniline (14)

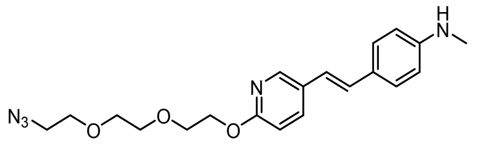

14

Compound **14** was prepared from compound **10** (1.01 g, 2.34 mmol) and copper (15 mg, 0.22 mmol) in ethanol (4 mL) and 40% aqueous CH_3NH_2 (2 mL, 22 mmol). The crude residue was purified by flash column chromatography on silica gel (40% EtOAc in hexane) to afford the desired product **14** (610 mg, 68%) as a yellowish solid. $C_{20}H_{25}N_5O_3$; mp 48–50 °C; TLC (30% EtOAc in hexane) $R_f = 0.30$; ^{1}H NMR (500 MHz, $CDCl_3$) δ 8.11 (d, $J = 2.2$ Hz, 1H), 7.73 (dd, $J = 8.6$, 2.4 Hz, 1H), 7.33 (d, $J = 8.5$ Hz, 2H), 6.88 (d, $J = 16.2$ Hz, 1H), 6.78 (d, $J = 16.2$ Hz, 1H), 6.74 (d, $J = 8.6$ Hz, 1H), 6.62–6.60 (m, 2H), 4.46 (td, $J = 4.7$, 3.4 Hz, 2H), 3.84 (td, $J = 4.7$, 3.4 Hz, 2H), 3.72–3.70 (m, 2H), 3.67–3.64 (m, 4H), 3.36 (t, $J = 5.2$ Hz, 2H), 2.84 (s, 3H); ^{13}C NMR (125 MHz, $CDCl_3$) δ 162.5, 148.3, 144.9, 135.0, 128.1, 127.5 (2 ×), 127.4, 127.0, 120.6, 112.9, 112.8, 111.2, 70.6 (2 ×), 70.0, 69.8, 65.1, 50.6, 30.9; ESI-HRMS calcd for $C_{20}H_{25}N_5NaO_3$ [M + Na]$^+$: 406.1849, found: *m/z* 406.1851.

(*E*)-4-(2-(6-(2-(2-(2-(2-Azidoethoxy)ethoxy)ethoxy)ethoxy)pyridin-3-yl)vinyl)-*N*-methylaniline (15)

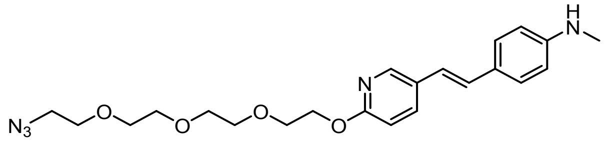

15

Compound **15** was prepared from compound **11** (750 mg, 1.57 mmol) and copper (15 mg, 0.22 mmol) in ethanol (3 mL) and 40% aqueous CH_3NH_2 (3 mL, 33 mmol). The crude residue was purified by flash column chromatography on silica gel (50% EtOAc in hexane) to afford the desired product **15** (490 mg, 73%) as a yellowish foam. $C_{22}H_{29}N_5O_4$; TLC (50% EtOAc in hexane) $R_f = 0.28$; ^{1}H NMR (400 MHz, $CDCl_3$) δ 8.11 (d, $J = 2.3$ Hz, 1H), 7.72 (dd, $J = 8.6$, 2.4 Hz, 1H), 7.31 (d, $J = 8.5$ Hz, 2H), 6.87 (d, $J = 16.3$ Hz, 1H), 6.77 (d, $J = 16.3$ Hz, 1H), 6.73 (d, $J = 8.6$ Hz, 1H), 6.57 (d, $J = 8.4$ Hz, 2H), 4.45 (t, $J = 4.7$ Hz, 2H), 3.83 (t, $J = 4.8$ Hz, 2H), 3.71–3.61 (m, 10H), 3.34 (t, $J = 5.2$ Hz, 2H), 2.82 (s, 3H); ^{13}C NMR (100 MHz, $CDCl_3$) δ 162.4, 148.8, 144.8, 135.0, 128.2, 127.5 (3 ×), 126.5, 120.3, 112.4 (2 ×), 111.1, 70.6 (4 ×), 69.9, 69.6, 65.1, 50.6, 30.6; ESI-HRMS calcd for $C_{22}H_{29}N_5NaO_4$ [M + Na]$^+$: 450.2111, found: *m/z* 450.2111.

(*E*)-4-(2-(6-((14-Azido-3,6,9,12-tetraoxatetradecyl)oxy)pyridin-3-yl)vinyl)-*N*-methylaniline (16)

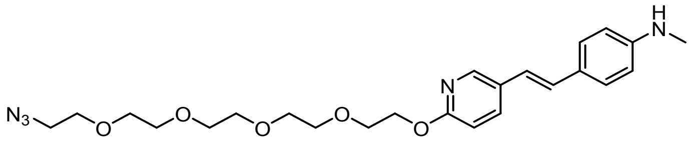

16

Compound **15** was prepared from compound **12** (854 mg, 1.63 mmol) and copper (15 mg, 0.22 mmol) in ethanol (3 mL) and 40% aqueous CH_3NH_2 (3 mL, 33 mmol). The crude residue was purified by flash column chromatography on silica gel (50% EtOAc in hexane) to afford the desired product **16** (538 mg, 70%) as a yellowish foam. $C_{24}H_{33}N_5O_5$; TLC (50% EtOAc in hexane) R_f = 0.26; ^{1}H NMR (500 MHz, $CDCl_3$) δ 8.10 (d, J = 2.2 Hz, 1H), 7.72 (dd, J = 8.7, 2.3 Hz, 1H), 7.30 (d, J = 8.3 Hz, 2H), 6.87 (d, J = 16.3 Hz, 1H), 6.77 (d, J = 16.3 Hz, 1H), 6.73 (d, J = 8.7 Hz, 1H), 6.56 (d, J = 8.3 Hz, 2H), 4.45 (t, J = 4.5 Hz, 2H), 3.82 (t, J = 4.6 Hz, 2H), 3.69–3.63 (m, 14H), 3.34 (t, J = 5.1 Hz, 2H), 2.82 (s, 3H); ^{13}C NMR (125 MHz, $CDCl_3$) δ 162.4, 148.8, 144.8, 134.9, 128.2, 127.5 (2 ×), 127.4, 126.3, 120.2, 112.4 (2 ×), 111.1, 70.5 (6 ×), 69.9, 69.6, 65.1, 50.5, 30.5; ESI-HRMS calcd for $C_{24}H_{33}N_5NaO_5$ $[M + Na]^+$: 494.2373, found: *m/z* 494.2379.

General synthetic procedure and product characterization of compounds (17–20)

To a solution of compound (**13**–**16**) in ethanol were added Boc_2O (4 equiv) and Et_3N (4 equiv). After stirring for 7 h at room temperature, the reaction mixture was concentrated and the residue was purified by flash column chromatography on silica gel to afford the desired product (**17**–**20**).

***tert*-Butyl (*E*)-(4-(2-(6-(2-(2-azidoethoxy)ethoxy)pyridin-3-yl)vinyl)phenyl) methyl carbamate (17)**

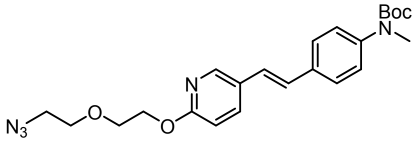

17

Compound **17** was prepared from compound **13** (170 mg, 0.5 mmol), Boc_2O (436 mg, 2 mmol) and Et_3N (0.28 mL, 2 mmol) in ethanol (4.6 mL). The crude residue was purified by flash column chromatography on silica gel (40% EtOAc in hexane) to afford the desired product **17** (197 mg, 90%) as a yellowish solid. $C_{23}H_{29}N_5O_4$; mp 106–108 °C; TLC (40% EtOAc in hexane) R_f = 0.34; ^{1}H NMR (500 MHz, $CDCl_3$) δ 8.16 (d, J = 2.2 Hz, 1H), 7.77 (dd, J = 8.7, 2.4 Hz, 1H), 7.41 (d, J = 8.5 Hz, 2H), 7.20 (d, J = 8.4 Hz, 2H), 6.94 (d, J = 1.3 Hz, 2H), 6.78 (d, J = 8.5 Hz, 1H), 4.49 (t, J = 4.6 Hz, 2H), 3.85 (t, J = 4.8 Hz, 2H), 3.71 (t, J = 5.0 Hz, 2H), 3.39 (t, J = 5.2 Hz, 2H), 3.24 (s, 3H), 1.43 (s, 9H); ^{13}C NMR (125 MHz, $CDCl_3$) δ 162.8, 154.6, 145.3, 143.1, 135.5, 134.0, 127.4,

126.8, 126.4 (2 ×), 125.4 (2 ×), 124.3, 111.4, 80.4, 70.0, 69.6, 65.2, 50.6, 37.1, 28.3 (3 ×); ESI-HRMS calcd for $C_{23}H_{30}N_5O_4$ [M + H]$^+$: 440.2292, found: *m/z* 440.2288.

***tert*-Butyl (*E*)-(4-(2-(6-(2-(2-(2-azidoethoxy)ethoxy)ethoxy)pyridin-3-yl)vinyl)phenyl) methyl carbamate (18)**

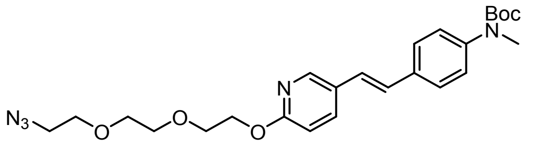

18

Compound **18** was prepared from compound **14** (479 mg, 1.25 mmol), Boc_2O (1.09 g, 5 mmol) and Et_3N (0.69 mL, 5 mmol) in ethanol (11.6 mL). The crude residue was purified by flash column chromatography on silica gel (40% EtOAc in hexane) to afford the desired product **18** (598 mg, 99%) as a yellowish sticky oil. $C_{25}H_{33}N_5O_5$; TLC (40% EtOAc in hexane) R_f= 0.32; ^{1}H NMR (500 MHz, $CDCl_3$) δ 8.16 (d, *J* = 2.3 Hz, 1H), 7.77 (dd, *J* = 8.7, 2.3 Hz, 1H), 7.41 (d, *J* = 8.5 Hz, 2H), 7.20–7.19 (m, 2H), 6.92 (d, *J* = 1.6 Hz, 2H), 6.77 (d, *J* = 8.6 Hz, 1H), 4.48 (td, *J* = 4.7, 3.4 Hz, 2H), 3.84 (td, *J* = 4.7, 3.4 Hz, 2H), 3.71–3.69 (m, 2H), 3.67–3.64 (m, 4H), 3.35 (t, *J* = 5.2 Hz, 2H), 3.24 (s, 3H), 1.42 (s, 9H); ^{13}C NMR (125 MHz, $CDCl_3$) δ 162.9, 154.6, 145.3, 143.1, 135.4, 134.0, 127.3, 126.7, 126.4 (2 ×), 125.4 (2 ×), 124.3, 111.4, 80.4, 70.6 (2 ×), 70.0, 69.7, 65.3, 50.6, 37.1, 28.2 (3 ×); ESI-HRMS calcd for $C_{25}H_{33}N_5NaO_5$ [M + Na]$^+$: 506.2373, found: *m/z* 506.2369.

***tert*-Butyl (*E*)-(4-(2-(6-(2-(2-(2-(2-azidoethoxy)ethoxy)ethoxy)ethoxy)pyridin-3-yl)vinyl) phenyl) methyl carbamate (19)**

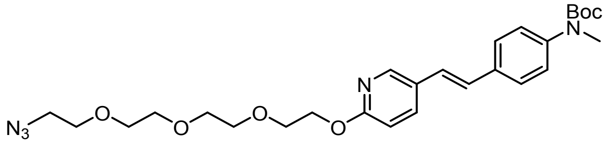

19

Compound **19** was prepared from compound **15** (480 mg, 1.12 mmol), Boc_2O (980 mg, 4.49 mmol) and Et_3N (0.62 mL, 4.49 mmol) in ethanol (10 mL). The crude residue was purified by flash column chromatography on silica gel (40% EtOAc in hexane) to afford the desired product **19** (584 mg, 99%) as a yellowish sticky oil. $C_{27}H_{37}N_5O_6$; TLC (50% EtOAc in hexane) R_f= 0.31; ^{1}H NMR (400 MHz, $CDCl_3$) δ 8.13 (d, *J* = 2.3 Hz, 1H), 7.74 (dd, *J* = 8.7, 2.4 Hz, 1H), 7.39 (d, *J* = 8.5 Hz, 2H), 7.18 (d, *J* = 8.5 Hz, 2H), 6.92 (d, *J* = 1.5 Hz, 2H), 6.74 (d, *J* = 8.7 Hz, 1H), 4.45 (t, *J* = 4.7 Hz, 2H), 3.82 (t, *J* = 4.8 Hz, 2H), 3.70–3.60 (m, 10H), 3.33 (t, *J* = 5.2 Hz, 2H), 3.22 (s, 3H), 1.42 (s, 9H); ^{13}C NMR (100 MHz, $CDCl_3$) δ 163.0, 154.5, 145.5, 143.0, 135.2, 134.0, 127.2, 126.6,

126.3 (2 ×), 125.3 (2 ×), 124.3, 111.3, 80.3, 70.5 (4 ×), 69.9, 69.6, 65.2, 50.5, 37.0, 28.2 (3 ×); ESI-HRMS calcd for $C_{27}H_{38}N_5O_6$ [M + H]$^+$: 528.2816, found: *m/z* 528.2810.

***tert*-Butyl (*E*)-(4-(2-(6-((14-azido-3,6,9,12-tetraoxatetradecyl)oxy)pyridin-3-yl)vinyl)phenyl) methyl carbamate (20)**

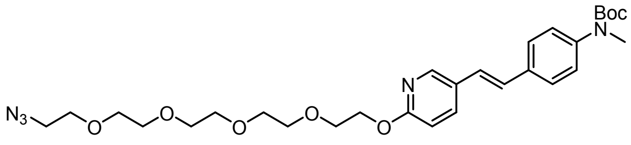

20

Compound **20** was prepared from compound **16** (471 mg, 1 mmol), Boc_2O (873 mg, 4 mmol) and Et_3N (0.55 mL, 4 mmol) in ethanol (10 mL). The crude residue was purified by flash column chromatography on silica gel (40% EtOAc in hexane) to afford the desired product **20** (566 mg, 99%) as a yellowish foam. $C_{29}H_{41}N_5O_7$; TLC (50% EtOAc in hexane) R_f = 0.29; ^{1}H NMR (400 MHz, $CDCl_3$) δ 8.14 (d, *J* = 2.4 Hz, 1H), 7.75 (dd, *J* = 8.7, 2.5 Hz, 1H), 7.40 (d, *J* = 8.5 Hz, 2H), 7.19 (d, *J* = 8.5 Hz, 2H), 6.93 (d, *J* = 1.6 Hz, 2H), 6.75 (d, *J* = 8.7 Hz, 1H), 4.46 (t, *J* = 4.7 Hz, 2H), 3.82 (t, *J* = 4.8 Hz, 2H), 3.69–3.62 (m, 14H), 3.34 (t, *J* = 5.2 Hz, 2H), 3.23 (s, 3H), 1.42 (s, 9H); ^{13}C NMR (100 MHz, $CDCl_3$) δ 163.0, 154.5, 145.5, 143.1, 135.3, 134.0, 127.2, 126.6, 126.3 (2 ×), 125.4 (2 ×), 124.4, 111.3, 80.3, 70.6 (5 ×), 70.5, 69.9, 69.6, 65.2, 50.6, 37.1, 28.2 (3 ×); ESI-HRMS calcd for $C_{29}H_{41}N_5NaO_7$ [M + Na]$^+$: 594.2898, found: *m/z* 594.2889.

General synthetic procedure and product characterization of compounds (21–24)

To a solution of compound (**17**–**20**) in THF and H_2O was added PPh_3 (3 equiv). After stirring for 15 h at room temperature, the reaction mixture was concentrated and the residue was purified by flash column chromatography on silica gel to afford the desired product (**21**–**24**).

***tert*-Butyl (*E*)-(4-(2-(6-(2-(2-aminoethoxy)ethoxy)pyridin-3-yl)vinyl)phenyl) methyl carbamate (21)**

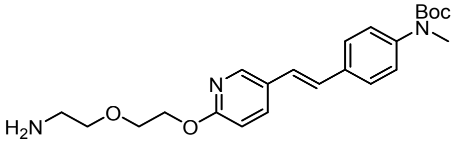

21

Compound **21** was prepared from compound **17** (191 mg, 0.43 mmol) and PPh_3 (343 mg, 1.31 mmol) in THF (3.21 mL) and H_2O (23 µL). The crude residue was purified by flash column chromatography on silica gel (10% CH_3OH in CH_2Cl_2) to afford the desired product **21** (173 mg,

96%) as a white solid. $C_{23}H_{31}N_3O_4$; mp 77–78 °C; TLC (5% CH_3OH in CH_2Cl_2) $R_f = 0.21$; ^{1}H NMR (400 MHz, $CDCl_3$) δ 8.13 (d, *J* = 2.2 Hz, 1H), 7.73 (dd, *J* = 8.7, 2.3 Hz, 1H), 7.38 (d, *J* = 8.5 Hz, 2H), 7.17 (d, *J* = 8.4 Hz, 2H), 6.90 (s, 2H), 6.74 (d, *J* = 8.6 Hz, 1H), 4.44 (t, *J* = 4.5 Hz, 2H), 3.78 (t, *J* = 4.5 Hz, 2H), 3.55–3.52 (m, 2H), 3.21 (s, 3H), 2.57–2.45 (m, 2H), 1.41 (s, 9H); ^{13}C NMR (100 MHz, $CDCl_3$) δ 162.9, 154.5, 145.5, 143.0, 135.3, 133.9, 127.2, 126.6, 126.3 (2 ×), 125.3 (2 ×), 124.3, 111.2, 80.3, 69.3, 65.1 (2 ×), 37.0, 30.8, 28.2 (3 ×); ESI-HRMS calcd for $C_{23}H_{32}N_3O_4$ $[M + H]^+$: 414.2387, found: *m/z* 414.2387.

tert-Butyl (_E_)-(4-(2-(6-(2-(2-(2-aminoethoxy)ethoxy)ethoxy)pyridin-3-yl)vinyl)phenyl) methyl carbamate (22)

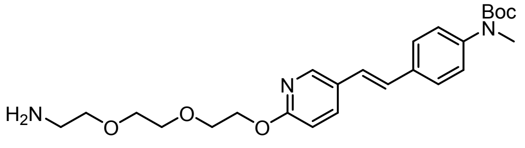

22

Compound **22** was prepared from compound **18** (590 mg, 1.22 mmol) and PPh_3 (944 mg, 3.6 mmol) in THF (9 mL) and H_2O (64 µL). The crude residue was purified by flash column chromatography on silica gel (10% CH_3OH in CH_2Cl_2) to afford the desired product **22** (536 mg, 96%) as a white solid. $C_{25}H_{35}N_3O_5$; mp 75–77 °C; TLC (5% CH_3OH in CH_2Cl_2) $R_f = 0.20$; ^{1}H NMR (500 MHz, $CDCl_3$) δ 8.14 (d, *J* = 2.8 Hz, 1H), 7.75 (dd, *J* = 8.7, 2.4 Hz, 1H), 7.40 (d, *J* = 8.5 Hz, 2H), 7.19–7.18 (m, 2H), 6.92 (d, *J* = 2.0 Hz, 2H), 6.77 (d, *J* = 8.6 Hz, 1H), 4.46 (t, *J* = 4.6 Hz, 2H), 3.83 (t, *J* = 4.7 Hz, 2H), 3.69–3.66 (m, 2H), 3.63–3.61 (m, 2H), 3.52 (t, *J* = 5.1 Hz, 2H), 3.23 (s, 3H), 2.87 (br s, 2H), 1.42 (s, 9H); ^{13}C NMR (125 MHz, $CDCl_3$) δ 162.9, 154.6, 145.5, 143.1, 135.3, 134.0, 127.2, 126.7, 126.4 (2 ×), 125.4 (2 ×), 124.3, 111.3, 80.4, 72.3, 70.5, 70.2 69.6, 65.2, 41.3, 37.1, 28.2 (3 ×); ESI-HRMS calcd for $C_{25}H_{36}N_3O_5$ $[M + H]^+$: 458.2649, found: *m/z* 458.2642.

tert-Butyl (_E_)-(4-(2-(6-(2-(2-(2-(2-aminoethoxy)ethoxy)ethoxy)ethoxy)pyridin-3-yl)vinyl)phenyl) methyl carbamate (23)

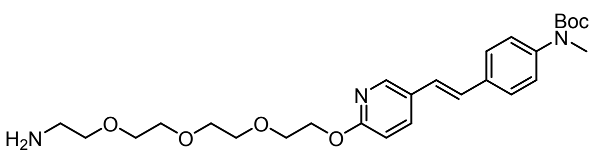

23

Compound **23** was prepared from compound **19** (527 mg, 1 mmol) and PPh_3 (787 mg, 3 mmol) in THF (7.36 mL) and H_2O (53 µL). The crude residue was purified by flash column chromatography on silica gel (10% CH_3OH in CH_2Cl_2) to afford the desired product **23** (481 mg, 96%) as a white solid. $C_{27}H_{39}N_3O_6$; mp 61–62 °C; TLC (5% CH_3OH in CH_2Cl_2) $R_f = 0.18$; ^{1}H NMR (400 MHz,

CDCl3) δ 8.14 (d, *J* = 2.3 Hz, 1H), 7.75 (dd, *J* = 8.7, 2.3 Hz, 1H), 7.40 (d, *J* = 8.5 Hz, 2H), 7.19 (d, *J* = 8.5 Hz, 2H), 6.92 (d, *J* = 1.2 Hz, 2H), 6.75 (d, *J* = 8.6 Hz, 1H), 4.46 (t, *J* = 4.7 Hz, 2H), 3.83 (t, *J* = 4.6 Hz, 2H), 3.70–3.58 (m, 8H), 3.49 (t, *J* = 5.2 Hz, 2H), 3.23 (s, 3H), 2.84 (t, *J* = 5.0 Hz, 2H), 1.43 (s, 9H); ^{13}C NMR (100 MHz, $CDCl_3$) δ 163.0, 154.6, 145.6, 143.1, 135.3, 134.1, 127.3, 126.7, 126.4 (2 ×), 125.4 (2 ×), 124.4, 111.3, 80.3, 72.9, 70.6, 70.5 (2 ×), 70.2, 69.6, 65.2, 41.6, 37.1, 28.3 (3 ×); ESI-HRMS calcd for $C_{27}H_{40}N_3O_6$ $[M + H]^+$: 502.2911, found: *m/z* 502.2920.

***tert*-Butyl (*E*)-(4-(2-(6-((14-amino-3,6,9,12-tetraoxatetradecyl)oxy)pyridin-3-yl)vinyl) phenyl) methyl carbamate (24)**

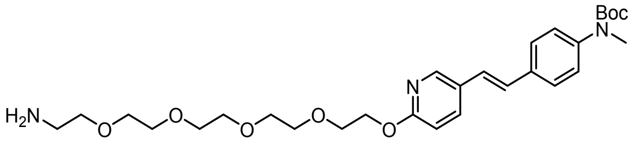

24

Compound **24** was prepared from compound **20** (514 mg, 0.9 mmol) and PPh_3 (708 mg, 2.7 mmol) in THF (6.5 mL) and H_2O (46 μL). The crude residue was purified by flash column chromatography on silica gel (10% CH_3OH in CH_2Cl_2) to afford the desired product **24** (476 mg, 97%) as a white solid. $C_{29}H_{43}N_3O_7$; mp 55–56 °C; TLC (5% CH_3OH in CH_2Cl_2) R_f = 0.16; ^{1}H NMR (500 MHz, $CDCl_3$) δ 8.15 (s, 1H), 7.77 (dd, *J* = 8.5, 1.5 Hz, 1H), 7.41 (d, *J* = 8.3 Hz, 2H), 7.19 (d, *J* = 8.1 Hz, 2H), 6.93 (s, 2H), 6.78 (d, *J* = 8.6 Hz, 1H), 4.46 (t, *J* = 4.4 Hz, 2H), 3.83 (t, *J* = 4.4 Hz, 2H), 3.69–3.61 (m, 12H), 3.51 (t, *J* = 5.0 Hz, 2H), 2.24 (s, 3H), 2.85 (t, *J* = 5.0 Hz, 2H), 1.43 (s, 9H); ^{13}C NMR (125 MHz, $CDCl_3$) δ 162.9, 154.6, 145.5, 143.1, 135.3, 134.0, 127.2, 126.7, 126.4 (2 ×), 125.4 (2 ×), 124.4, 111.3, 80.4, 72.7, 70.6, 70.5, 70.4 (3 ×), 70.2, 69.6, 65.2, 41.5, 37.1, 28.3 (3 ×); ESI-HRMS calcd for $C_{29}H_{44}N_3O_7$ $[M + H]^+$: 546.3173, found: *m/z* 546.3167.

General synthetic procedure and product characterization of compounds (25–28)

To a solution of compound (**21**–**24**), E3-ligand (2 equiv) in *N*-methyl-2-pyrrolidone was added *i*Pr_2NEt (2 equiv). After stirring for 18 h at 90 °C, the reaction mixture was diluted with EtOAc and washed with H_2O. The aqueous layer was extracted with EtOAc (3 ×) and the combined organic extracts were dried over $MgSO_4$, filtered and concentrated. The residue was purified by flash column chromatography on silica gel to afford the desired product (**25**–**28**).

***tert*-Butyl (*E*)-(4-(2-(6-(2-(2-((2-(2,6-dioxopiperidin-3-yl)-1,3-dioxoisoindolin-4-yl)amino) ethoxy)ethoxy)pyridin-3-yl)vinyl)phenyl) methyl carbamate (25)**

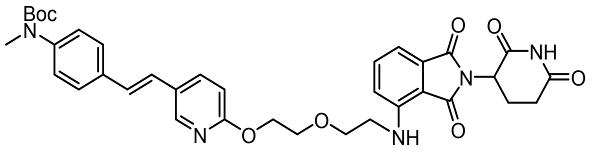

25

Compound **25** was prepared from compound **21** (161 mg, 0.39 mmol), E3-ligand (216 mg, 0.78 mmol) and *i*Pr_2NEt (136 µL, 0.78 mmol) in *N*-methyl-2-pyrrolidone (2.2 mL). The crude residue was purified by flash column chromatography on silica gel (2% CH_3OH in CH_2Cl_2) to afford the desired product **25** (159 mg, 61%) as a yellow solid. $C_{36}H_{39}N_5O_8$; mp 112–114 °C; TLC (2% CH_3OH in CH_2Cl_2) R_f = 0.32. ^{1}H NMR (400 MHz, $CDCl_3$) δ 8.27 (br s, 1H), 8.14 (s, 1H), 7.76 (dd, *J* = 8.7, 2.0 Hz, 1H), 7.45–7.41 (m, 3H), 7.20 (d, *J* = 8.5 Hz, 2H), 7.05 (d, *J* = 7.0 Hz, 1H), 6.93 (s, 2H), 6.89 (d, *J* = 8.6 Hz, 1H), 6.75 (d, *J* = 8.6 Hz, 1H), 6.48 (br s, 1H), 4.88 (dd, *J* = 12.2, 5.5 Hz, 1H), 4.48 (t, *J* = 4.3 Hz, 2H), 3.84 (t, *J* = 4.6 Hz, 2H), 3.75 (t, *J* = 5.4 Hz, 2H), 3.47–3.45 (m, 2H), 3.24 (s, 3H), 2.85–2.80 (m, 1H), 2.77–2.64 (m, 2H), 2.09–2.05 (m, 1H), 1.43 (s, 9H); ^{13}C NMR (100 MHz, $CDCl_3$) δ 171.0, 169.2, 168.3, 167.5, 162.8, 154.6, 146.8, 145.4, 143.1, 135.9, 135.4, 134.0, 132.4, 127.4, 126.7, 126.4 (2 ×), 125.4 (2 ×), 124.3, 116.7, 111.6, 111.4, 110.2, 80.4, 69.6, 69.5, 65.2, 48.8, 42.3, 37.1, 31.3, 28.3 (3 ×), 22.7; ESI-HRMS calcd for $C_{36}H_{39}N_5NaO_8$ [M + Na]$^+$: 692.2690, found: *m/z* 692.2681.

***tert*-Butyl (*E*)-(4-(2-(6-(2-(2-(2-((2-(2,6-dioxopiperidin-3-yl)-1,3-dioxoisoindolin-4-yl)amino) ethoxy)ethoxy)ethoxy)pyridin-3-yl)vinyl)phenyl) methyl carbamate (26)**

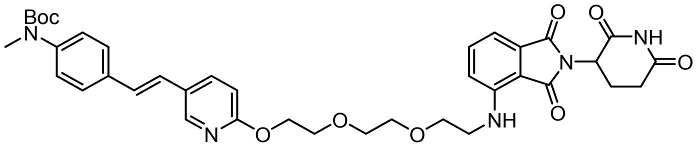

26

Compound **26** was prepared from compound **22** (515 mg, 1.12 mmol), E3-ligand (464 mg, 1.68 mmol) and *i*Pr_2NEt (0.39 mL, 2.24 mmol) in *N*-methyl-2-pyrrolidone (6 mL). The crude residue was purified by flash column chromatography on silica gel (2% CH_3OH in CH_2Cl_2) to afford the desired product **26** (464 mg, 58%) as a yellow solid. $C_{38}H_{43}N_5O_9$; mp 87–89 °C; TLC (2% CH_3OH in CH_2Cl_2) R_f = 0.31; ^{1}H NMR (400 MHz, $CDCl_3$) δ 8.83 (br s, 1H), 8.15 (d, *J* = 2.3 Hz, 1H), 7.74 (dd, *J* = 8.7, 2.4 Hz, 1H), 7.44–7.38 (m, 3H), 7.20–7.17 (m, 2H), 7.04 (d, *J* = 7.1 Hz, 1H), 6.91 (s, 2H), 6.86 (d, *J* = 8.5 Hz, 1H), 6.75 (d, *J* = 8.7 Hz, 1H), 6.47 (br s, 1H), 4.86 (dd, *J* = 12.0, 5.6 Hz, 1H), 4.46 (t, *J* = 4.6 Hz, 2H), 3.85 (td, *J* = 5.0, 1.3 Hz, 2H), 3.72–3.64 (m, 6H), 3.44–3.41 (m, 2H), 3.23 (s, 3H), 2.83–2.77 (m, 1H), 2.73–2.65 (m, 2H), 2.09–2.04 (m, 1H), 1.42 (s, 9H); ^{13}C NMR (100 MHz, $CDCl_3$) δ 171.2, 169.2, 168.5, 167.5, 162.9, 154.5, 146.7, 145.2, 143.1, 135.9, 135.5, 134.0, 132.4, 127.3, 126.7, 126.3 (2 ×), 125.4 (2 ×), 124.2, 116.6, 111.5, 111.4, 110.2, 80.3, 70.8,

70.5, 69.8, 69.3, 65.4, 48.8, 42.2, 37.1, 31.2, 28.2 (3 ×), 22.7; ESI-HRMS calcd for $C_{38}H_{44}N_5O_9$ $[M + H]^+$: 714.3133, found: *m/z* 714.3142.

tert-Butyl (_E_)-(4-(2-(6-(2-(2-(2-(2-((2-(2,6-dioxopiperidin-3-yl)-1,3-dioxoisoindolin-4-yl)amino)ethoxy)ethoxy)ethoxy)ethoxy)pyridin-3-yl)vinyl)phenyl)(methyl)carbamate (27)

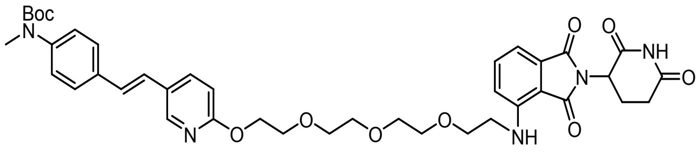

27

Compound **27** was prepared from compound **23** (250 mg, 0.5 mmol), E3-ligand (276 mg, 1 mmol) and *i*Pr_2NEt (0.17 mL, 1 mmol) in *N*-methyl-2-pyrrolidone (3 mL). The crude residue was purified by flash column chromatography on silica gel (2% CH_3OH in CH_2Cl_2) to afford the desired product **27** (178 mg, 47%) as a yellow solid. $C_{40}H_{47}N_5O_{10}$; mp 79–81 °C; TLC (2% CH_3OH in CH_2Cl_2) R_f = 0.29; ^1H NMR (500 MHz, $CDCl_3$) δ 8.43 (s, 1H), 8.14 (d, *J* = 1.3 Hz, 1H), 7.76 (dd, *J* = 8.5, 1.5 Hz, 1H), 7.44 (t, *J* = 7.4 Hz, 1H), 7.40 (d, *J* = 8.4 Hz, 2H), 7.19 (d, *J* = 8.2 Hz, 2H), 7.05 (d, *J* = 7.0 Hz, 1H), 6.92 (s, 2H), 6.87 (d, *J* = 8.5 Hz, 1H), 6.76 (d, *J* = 8.5 Hz, 1H), 6.45 (br s, 1H), 4.87 (dd, *J* = 12.1, 5.2 Hz, 1H), 4.46 (t, *J* = 4.4 Hz, 2H), 3.83 (t, *J* = 4.6 Hz, 2H), 3.69–3.64 (m, 10H), 3.48–3.42 (m, 2H), 3.24 (s, 3H), 2.85–2.80 (m, 1H), 2.77–2.65 (m, 2H), 2.10–2.05 (m, 1H), 1.43 (s, 9H); ^{13}C NMR (125 MHz, $CDCl_3$) δ 171.1, 169.1, 168.3, 167.5, 162.9, 154.5, 146.7, 145.4, 143.1, 135.9, 135.4, 134.0, 132.4, 127.3, 126.7, 126.3 (2 ×), 125.4 (2 ×), 124.3, 116.7, 111.5, 111.4, 110.2, 80.4, 70.6 (4 ×), 69.6, 69.4, 65.4, 48.8, 42.3, 37.1, 31.3, 28.2 (3 ×), 22.7; ESI-HRMS calcd for $C_{40}H_{47}N_5NaO_{10}$ $[M + Na]^+$: 780.3215, found: *m/z* 780.3215.

tert-Butyl (_E_)-(4-(2-(6-((14-((2-(2,6-dioxopiperidin-3-yl)-1,3-dioxoisoindolin-4-yl)amino)-3,6,9,12-tetraoxatetradecyl)oxy)pyridin-3-yl)vinyl)phenyl)(methyl)carbamate (28)

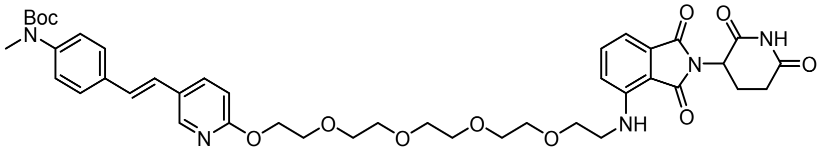

28

Compound **28** was prepared from compound **24** (270 mg, 0.5 mmol), E3-ligand (276 mg, 1 mmol) and *i*Pr_2NEt (0.17 mL, 1 mmol) in *N*-methyl-2-pyrrolidone (3 mL). The crude residue was purified by flash column chromatography on silica gel (2% CH_3OH in CH_2Cl_2) to afford the desired product **28** (230 mg, 58%) as a yellow solid. $C_{42}H_{51}N_5O_{11}$; mp 68–70 °C; TLC (2% CH_3OH in CH_2Cl_2) R_f = 0.28; ^1H NMR (500 MHz, $CDCl_3$) δ 8.46 (s, 1H), 8.14 (s, 1H), 7.74 (d, *J* = 7.1 Hz, 1H), 7.44 (t, *J* = 8.0 Hz, 1H), 7.40 (d, *J* = 8.5 Hz, 2H), 7.19 (d, *J* = 8.1 Hz, 2H), 7.05 (d, *J* = 7.0 Hz, 1H), 6.92

(d, J = 1.3 Hz, 2H), 6.87 (d, J = 8.5 Hz, 1H), 6.74 (d, J = 8.5 Hz, 1H), 6.45 (br s, 1H), 4.87 (dd, J = 12.0, 5.0 Hz, 1H), 4.45 (t, J = 4.2 Hz, 2H), 3.82 (t, J = 4.36 Hz, 2H), 3.68–3.63 (m, 14H), 3.43–3.42 (m, 2H), 3.23 (s, 3H), 2.84–2.79 (m, 1H), 2.76–2.63 (m, 2H), 2.08–2.05 (m, 1H), 1.43 (s, 9H); ^{13}C NMR (125 MHz, $CDCl_3$) δ 171.1, 159.2, 168.3, 167.5, 162.9, 154.6, 146.7, 145.5, 143.1, 135.9, 135.3, 134.0, 132.4, 127.2, 126.6, 126.3 (2 ×), 125.4 (2 ×), 124.3, 116.7, 111.5, 111.3, 110.2, 80.4, 70.6 (2 ×), 70.5 (4 ×), 69.6, 69.4, 65.2, 48.8, 42.3, 37.1, 31.3, 28.2 (3 ×), 22.7; ESI-HRMS calcd for $C_{42}H_{51}N_5NaO_{11}$ $[M + Na]^+$: 824.3477, found: m/z 824.3471.

General synthetic procedure and product characterization of compounds (PROTAC 1’–PROTAC 4’)

To a solution of compound (**21**–**24**) in CH_2Cl_2 was slowly added CF_3CO_2H at 0 °C. After stirring for 2 h at room temperature, the reaction mixture was concentrated and azeotrope with toluene (× 3) to remove CF_3CO_2H. The residue was dissolved in CH_2Cl_2, the solution was neutralized with aqueous saturated $NaHCO_3$ and extracted with CH_2Cl_2 (3 ×). The combined organic extracts were dried over $MgSO_4$, filtered and concentrated. The residue was purified by flash column chromatography on silica gel to afford the desired product (**PROTAC 1’**–**PROTAC 4’**).

PROTAC 1’

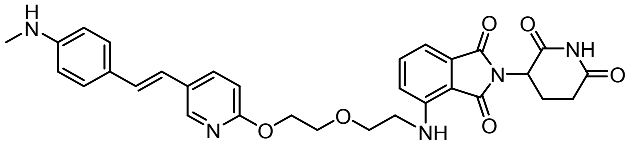

PROTAC 1'

PROTAC 1’ was prepared from compound **25** (151 mg, 0.22 mmol) and CF_3CO_2H (0.5 mL) in CH_2Cl_2 (2 mL). The crude residue was purified by flash column chromatography on silica gel (2% CH_3OH in CH_2Cl_2) to afford the desired product **PROTAC 1’** (110 mg, 86%) as a yellow solid. $C_{31}H_{31}N_5O_6$; mp 122–124 °C; TLC (2% CH_3OH in CH_2Cl_2) R_f= 0.26; ^1H NMR (500 MHz, $CDCl_3$) δ 8.12 (br s, 1H), 8.10 (d, J = 2.0 Hz, 1H), 7.72 (dd, J = 8.6, 2.2 Hz, 1H), 7.44 (t, J = 8.2 Hz, 1H), 7.34 (d, J = 8.5 Hz, 2H), 7.06 (d, J = 7.0 Hz, 1H), 6.89 (d, J = 8.4 Hz, 1H), 6.87 (d, J = 16.2 Hz, 1H), 6.80 (d, J = 16.2 Hz, 1H), 6.72 (d, J = 8.6 Hz, 1H), 6.68 (d, J = 8.4 Hz, 2H), 6.48 (t, J = 4.8 Hz, 1H), 4.86 (dd, J = 11.5, 4.5 Hz, 1H), 4.47 (t, J = 4.5 Hz, 2H), 3.84 (t, J = 4.6 Hz, 2H), 3.75 (t, J = 5.4 Hz, 2H), 3.48–3.45 (m, 2H), 2.86 (s, 3H), 2.82–2.79 (m, 1H), 2.77–2.64 (m, 2H), 2.08–2.05 (m, 1H); ^{13}C NMR (125 MHz, $CDCl_3$) δ 170.9, 169.1, 168.2, 167.5, 162.5, 147.4, 146.8, 144.9, 136.0, 135.1, 132.4, 128.0, 127.8, 127.6 (2 ×), 127.4, 120.9, 116.7, 113.6 (2 ×), 111.6, 111.2, 110.2, 69.7, 69.5, 65.1, 48.8, 42.4, 31.4, 31.3, 22.7; ESI-HRMS calcd for $C_{31}H_{31}N_5NaO_6$ $[M + Na]^+$: 592.2166, found: m/z 592.2157.

PROTAC 2'

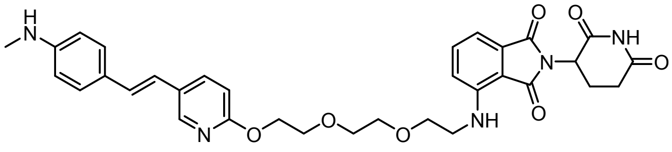

PROTAC 2'

PROTAC 2' was prepared from compound **26** (455 mg, 0.63 mmol) and CF_3CO_2H (2 mL) in CH_2Cl_2 (8 mL). The crude residue was purified by flash column chromatography on silica gel (2% CH_3OH in CH_2Cl_2) to afford the desired product **PROTAC 2'** (324 mg, 84%) as a yellow solid. $C_{33}H_{35}N_5O_7$; mp 98–100 °C; TLC (2% CH_3OH in CH_2Cl_2) R_f= 0.25; ^{1}H NMR (500 MHz, $CDCl_3$) δ 8.80 (br s, 1H), 8.11 (d, *J* = 2.2 Hz, 1H), 7.71 (dd, *J* = 8.6, 2.3 Hz, 1H), 7.42 (t, *J* = 8.3 Hz, 1H), 7.30 (d, *J* = 8.5 Hz, 2H), 7.05 (d, *J* = 7.0 Hz, 1H), 6.87–6.84 (m, 2H), 6.77 (d, *J* = 16.3 Hz, 1H), 6.72 (d, *J* = 8.7 Hz, 1H), 6.58 (d, *J* = 8.5 Hz, 2H), 6.47 (t, *J* = 5.1 Hz, 1H), 4.86 (dd, *J* = 12.2, 5.4 Hz, 1H), 4.45 (t, *J* = 4.5 Hz, 2H), 3.86–3.84 (m, 2H), 3.72–3.65 (m, 6H), 3.42–3.39 (m, 2H), 2.82 (s, 3H), 2.80–2.75 (m, 1H), 2.73–2.62 (m, 2H), 2.08–2.04 (m, 1H); ^{13}C NMR (125 MHz, $CDCl_3$) δ 171.3, 169.2, 168.5, 167.5, 162.4, 148.5, 146.7, 144.8, 135.9, 135.0, 132.4, 128.2, 127.5 (2 ×), 127.4, 126.6, 120.3, 116.7, 112.6 (2 ×), 111.5, 111.2, 110.2, 70.8, 70.5, 69.8, 69.3, 65.2, 48.7, 42.2, 31.2, 30.7, 22.7; ESI-HRMS calcd for $C_{33}H_{36}N_5O_7$ $[M + H]^+$: 614.2609, found: *m/z* 614.2613.

PROTAC 3'

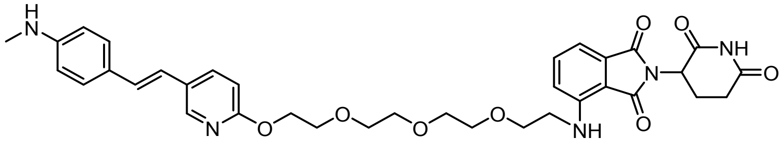

PROTAC 3'

PROTAC 3' was prepared from compound **27** (150 mg, 0.19 mmol) and CF_3CO_2H (0.5 mL) in CH_2Cl_2 (4.5 mL). The crude residue was purified by flash column chromatography on silica gel (2% CH_3OH in CH_2Cl_2) to afford the desired product **PROTAC 3'** (103 mg, 80%) as a yellow solid. $C_{35}H_{39}N_5O_8$; mp 138–140 °C; TLC (2% CH_3OH in CH_2Cl_2) R_f= 0.22; ^{1}H NMR (400 MHz, $CDCl_3$) δ 8.33 (s, 1H), 8.11 (d, *J* = 2.3 Hz, 1H), 7.72 (dd, *J* = 8.7, 2.4 Hz, 1H), 7.45 (t, *J* = 7.3 Hz, 1H), 7.32 (d, *J* = 8.5 Hz, 2H), 7.06 (d, *J* = 7.0 Hz, 1H), 6.89–6.85 (m, 2H), 6.78 (d, *J* = 16.5 Hz, 1H), 6.73 (d, *J* = 8.6 Hz, 1H), 6.67 (d, *J* = 8.5 Hz, 1H), 6.45 (br s, 1H), 4.87 (dd, *J* = 12.2, 5.1 Hz, 1H), 4.45 (t, *J* = 4.7 Hz, 2H), 3.83 (t, *J* = 4.6 Hz, 2H), 3.70–3.63 (m, 10H), 3.43–3.42 (m, 2H), 2.86 (s, 3H), 2.83–2.81 (m, 1H), 2.78–2.67 (m, 2H), 2.10–2.05 (m, 1H); ^{13}C NMR (125 MHz, $CDCl_3$) δ 171.2, 169.2, 168.4, 167.6, 162.4, 148.2, 146.7, 144.9, 135.9, 135.1, 132.4, 128.1, 127.5 (2 ×), 127.4, 127.0, 120.5, 116.7, 113.0 (2 ×), 111.5, 111.2, 110.2, 70.6 (2 ×), 70.5 (2 ×), 69.6,

69.4, 65.1, 48.8, 42.3, 31.3, 31.0, 22.7; ESI-HRMS calcd for $C_{35}H_{39}N_5NaO_8$ [M + Na]$^+$: 680.2690, found: *m/z* 680.2682.

PROTAC 4'

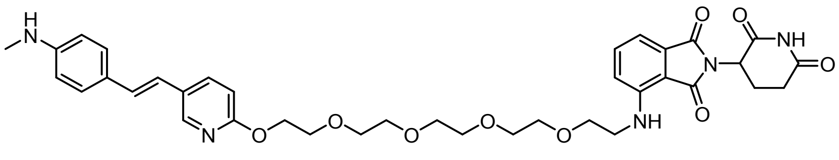

PROTAC 4'

PROTAC 4' was prepared from compound **28** (200 mg, 0.25 mmol) and CF_3CO_2H (0.5 mL) in CH_2Cl_2 (4.5 mL). The crude residue was purified by flash column chromatography on silica gel (2% CH_3OH in CH_2Cl_2) to afford the desired product **PROTAC 4'** (132 mg, 76%) as a yellow solid. $C_{37}H_{43}N_5O_9$; mp 78–80 °C; TLC (2% CH_3OH in CH_2Cl_2) R_f = 0.22; ^{1}H NMR (500 MHz, $CDCl_3$) δ 8.29 (br s, 1H), 8.10 (s, 1H), 7.72 (d, J = 8.43 Hz, 1H), 7.45 (t, J = 7.6 Hz, 1H), 7.32 (d, J = 8.1 Hz, 2H), 7.06 (d, J = 7.2 Hz, 1H), 6.89–6.85 (m, 3H), 6.78 (d, J = 16.2 Hz, 1H), 6.73 (d, J = 8.6 Hz, 1H), 6.66 (d, J = 8.0 Hz, 2H), 6.45 (br s, 1H), 4.87 (dd, J = 12.0, 5.0 Hz, 1H), 4.45 (t, J = 4.6 Hz, 2H), 3.83 (t, J = 4.7 Hz, 2H), 3.69–3.63 (m, 14H), 3.44–3.41 (m, 2H), 2.85 (s, 3H), 2.82–2.80 (m, 1H), 2.77–2.64 (m, 2H), 2.09–2.06 (m, 1H); ^{13}C NMR (125 MHz, $CDCl_3$) δ 171.1, 169.2, 168.3, 167.6, 162.5, 148.0, 146.7, 144.9, 135.9, 135.0, 132.4, 128.1, 127.5 (2 ×), 127.4, 127.1, 120.6, 116.7, 113.1 (2 ×), 111.5, 111.2, 110.2, 70.6 (2 ×), 70.5 (4 ×), 69.6, 69.4, 65.1, 48.8, 42.3, 31.3, 31.0, 22.7; ESI-HRMS calcd for $C_{37}H_{43}N_5NaO_9$ [M + Na]$^+$: 724.2953, found: *m/z* 724.2952.

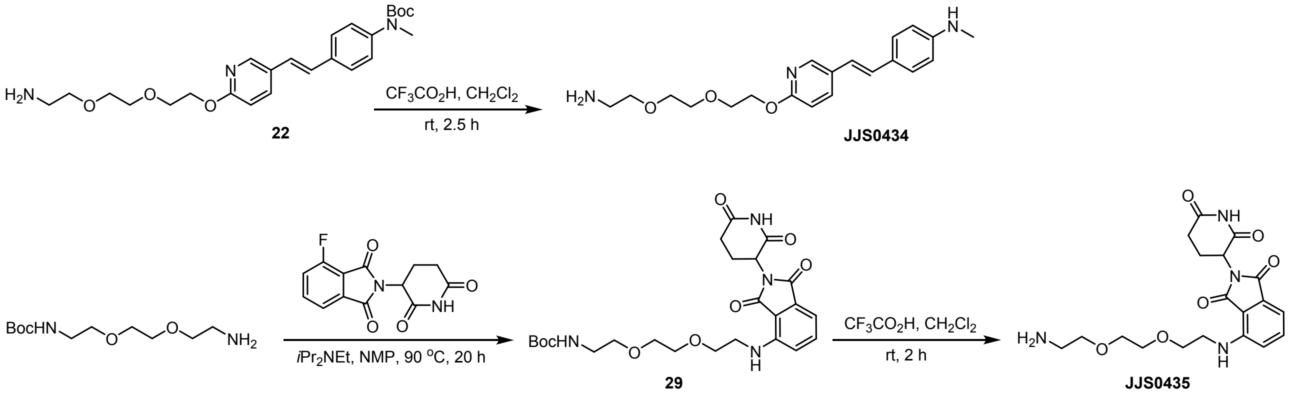

Scheme S3. Synthesis of control fragments (**JJS0434** and **JJS0435**) of PROTAC degrader

(*E*)-4-(2-(6-(2-(2-(2-Aminoethoxy)ethoxy)ethoxy)pyridin-3-yl)vinyl)-*N*-methylaniline (JJS0434)

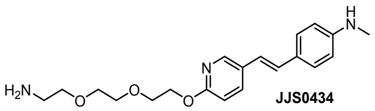

To a solution of compound **22** (22 mg, 0.04 mmol) in CH_2Cl_2 (2 mL) was slowly added CF_3CO_2H (0.4 mL) at 0 °C. After stirring for 2.5 h at room temperature, the reaction mixture was concentrated and azeotrope with toluene (3 ×). The residue was dissolved in CH_2Cl_2 and the solution was neutralized with aqueous saturated $NaHCO_3$ (3 mL). The aqueous layer was extracted with CH_2Cl_2 (3 ×) and the combined organic extracts were dried over $MgSO_4$, filtered and concentrated. The residue was purified by flash column chromatography on silica gel (10% CH_3OH in CH_2Cl_2) to afford the desired product **JJS0434** (13 mg, 83%) as a yellow solid. $C_{20}H_{27}N_3O_3$; mp 102–105 °C; TLC (10% CH_3OH in CH_2Cl_2) R_f = 0.19; ^1H NMR (500 MHz, $CDCl_3$) δ 8.11 (d, J = 3.5 Hz, 1H), 7.72 (d, J = 7.5 Hz, 1H), 7.31 (d, J = 7.7 Hz, 2H), 6.87 (d, J = 16.3 Hz, 1H), 6.76 (d, J = 16.5 Hz, 1H), 6.73 (s, 1H), 6.55 (d, J = 7.7 Hz, 2H), 4.44 (s, 2H), 3.83–3.28 (m, 10H), 2.82 (s, 3H); ^{13}C NMR (125 MHz, $CDCl_3$) δ 162.3, 149.0, 144.8, 135.1, 128.3, 127.6, 127.5 (2 ×), 126.3, 120.1, 112.3 (2 ×), 111.1, 70.6, 70.5, 70.2, 69.7 (2 ×), 65.1, 30.5; ESI-HRMS calcd for $C_{20}H_{28}N_3O_3$ $[M + H]^+$: 358.2125, found: *m/z* 358.2126.

***tert*-Butyl (2-(2-(2-((2-(2,6-dioxopiperidin-3-yl)-1,3-dioxoisoindolin-4-yl)amino)ethoxy) ethoxy)ethyl)carbamate (29)**

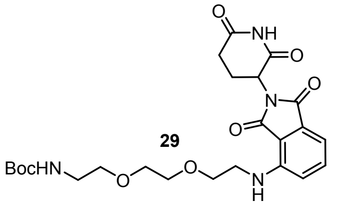

To a solution of Boc-containing linker (124 mg, 0.5 mmol) and E3-ligand (276 mg, 1 mmol) in *N*-methyl-2-pyrrolidone (7 mL) was added iPr$_2$NEt (0.26 mL, 1.5 mmol). After stirring for 20 h at 90 °C, the reaction mixture was diluted with EtOAc and washed with H_2O (3 ×). The combined aqueous layers were extracted with EtOAc (2 ×). The combined organic extracts were dried over $MgSO_4$, filtered and concentrated. The residue was purified by flash column chromatography on silica gel (1% CH_3OH in CH_2Cl_2) to afford the desired product **29** (121 mg, 48%) as a yellow foam. $C_{24}H_{32}N_4O_8$; TLC (1% CH_3OH in CH_2Cl_2); R_f = 0.31; ^1H NMR (500 MHz, $CDCl_3$) δ 8.50 (s, 1H), 7.46 (t, J = 7.9 Hz, 1H), 7.07 (d, J = 7.1 Hz, 1H), 6.88 (d, J = 8.5 Hz, 1H), 6.48 (s, 1H), 5.04 (s, 1H), 4.89–4.87 (m, 1H), 3.69 (t, J = 5.3 Hz, 2H), 3.63–3.60 (m, 4H), 3.54–3.52 (m, 2H), 3.46–3.42 (m, 2H), 3.29–3.28 (m, 2H), 2.85–2.79 (m, 1H), 2.76–2.69 (m, 2H), 2.11–2.07 (m, 1H), 1.39 (s, 9H); ^{13}C NMR (125 MHz, $CDCl_3$) δ 171.1, 169.3, 168.4, 167.5, 156.0, 146.7, 136.0, 132.5, 116.6, 111.6, 110.3, 79.2, 70.7, 70.3, 70.1, 69.3, 48.8, 42.2, 40.3, 31.3, 28.3 (3 ×), 22.8; ESI-HRMS calcd for $C_{24}H_{32}N_4NaO_8$ $[M + Na]^+$: 527.2112, found: *m/z* 527.2103.

4-((2-(2-(2-Aminoethoxy)ethoxy)ethyl)amino)-2-(2,6-dioxopiperidin-3-yl)isoindoline-1,3-dione (JJS0435)

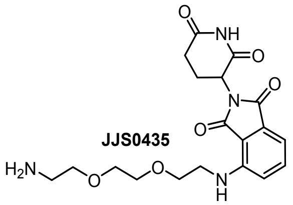

To a solution of compound **29** (20 mg, 0.03 mmol) in CH_2Cl_2 (1.8 mL) was added CF_3CO_2H (0.2 mL) at 0 °C. After stirring for 2 h at room temperature, the reaction mixture was concentrated and azeotrope with toluene (3 ×). The residue was dissolved in CH_2Cl_2 and the solution was neutralized with aqueous saturated $NaHCO_3$. The resulting mixture was extracted with CH_2Cl_2 (3 ×) and the combined organic extracts were dried over $MgSO_4$, filtered and concentrated. The residue was purified by flash column chromatography on silica gel (15% CH_3OH in CH_2Cl_2) to afford the desired product **JJS0435** (9 mg, 57%) as a yellow solid. $C_{19}H_{24}N_4O_6$; mp 146–149 °C; TLC (10% CH_3OH in CH_2Cl_2) R_f = 0.18; ^1H NMR (500 MHz, CD_3OD) δ 7.58 (t, *J* = 7.5 Hz, 1H), 7.11 (d, *J* = 8.7 Hz, 1H), 7.09 (d, *J* = 7.0 Hz, 1H), 5.06 (dd, *J* = 13.0, 5.5 Hz, 1H), 3.75 (t, *J* = 4.7 Hz, 2H), 3.70 (s, 6H), 3.53 (t, *J* = 4.8 Hz, 2H), 3.11 (t, *J* = 4.5 Hz, 2H), 2.90–2.83 (m, 1H), 2.78–2.66 (m, 2H), 2.13–2.11 (m, 1H); ^{13}C NMR (125 MHz, CD_3OD) δ 174.8, 171.8, 170.9, 169.3, 148.1, 137.4, 133.8, 118.3, 112.2, 111.2, 71.5, 71.3, 70.4, 67.9, 50.2, 43.0, 40.7, 32.1, 23.7; ESI-HRMS calcd for $C_{19}H_{25}N_4O_6$ $[M + H]^+$: 405.1768, found: *m/z* 405.1772.

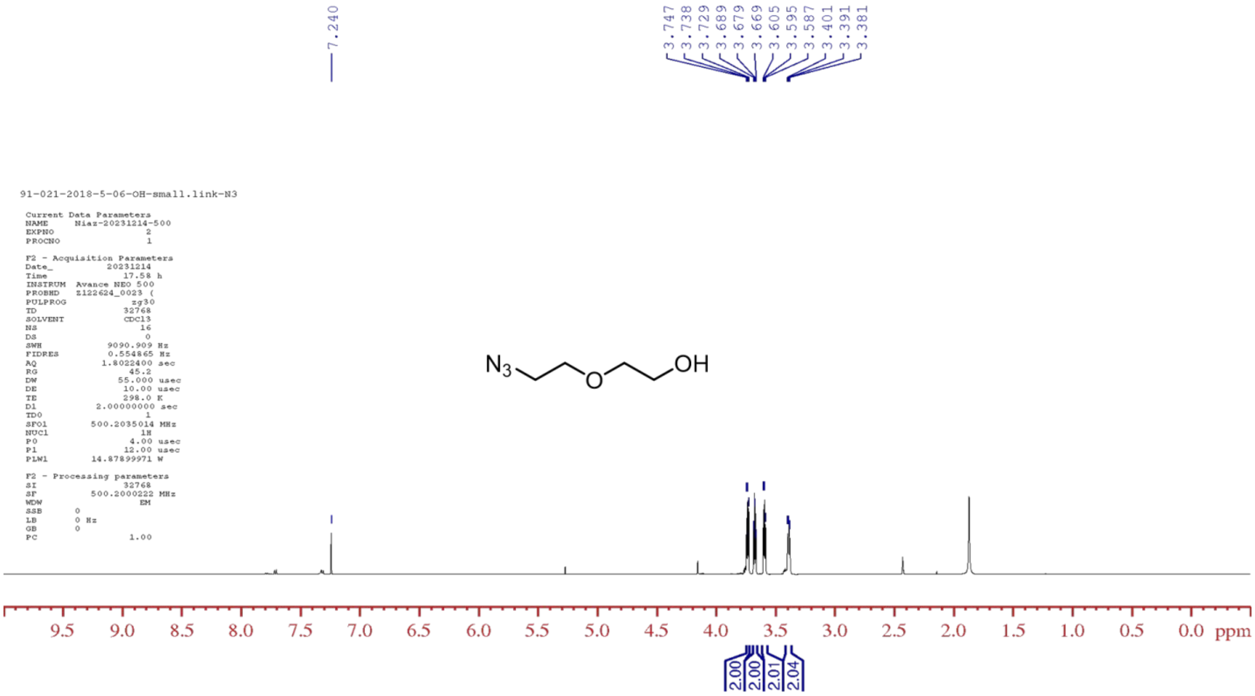

^{1}H NMR Spectrum of Compound **2** (500 MHz, $CDCl_3$)

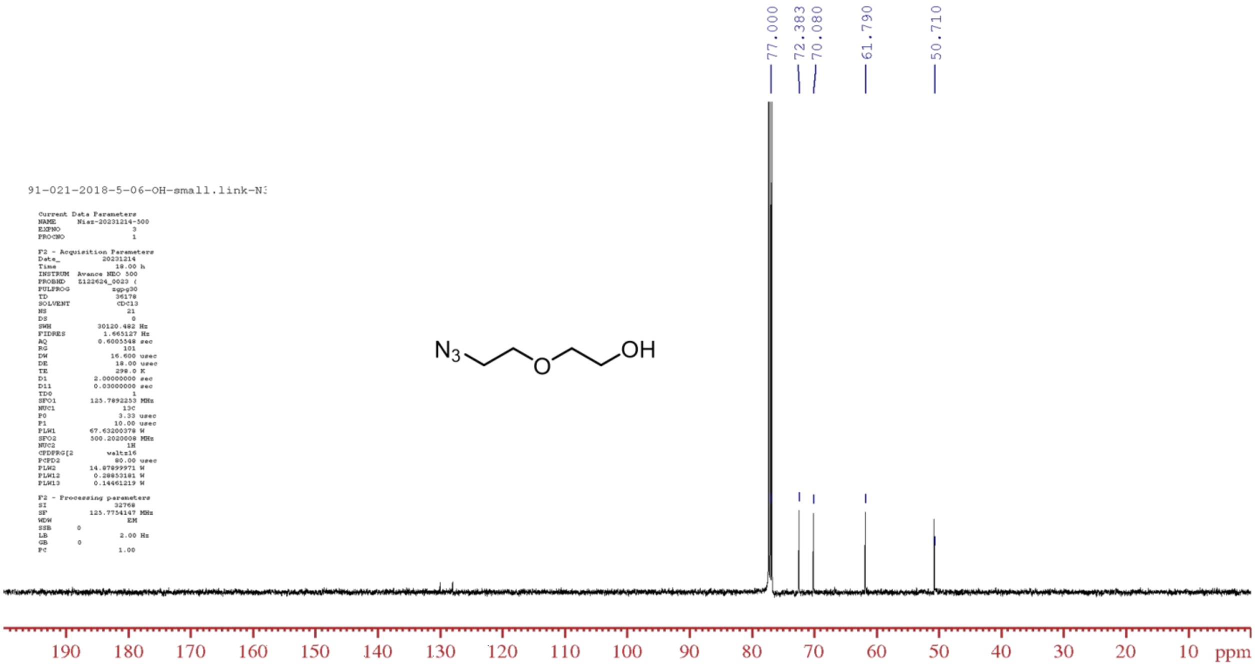

^{13}C NMR Spectrum of Compound **2** (125 MHz, $CDCl_3$)

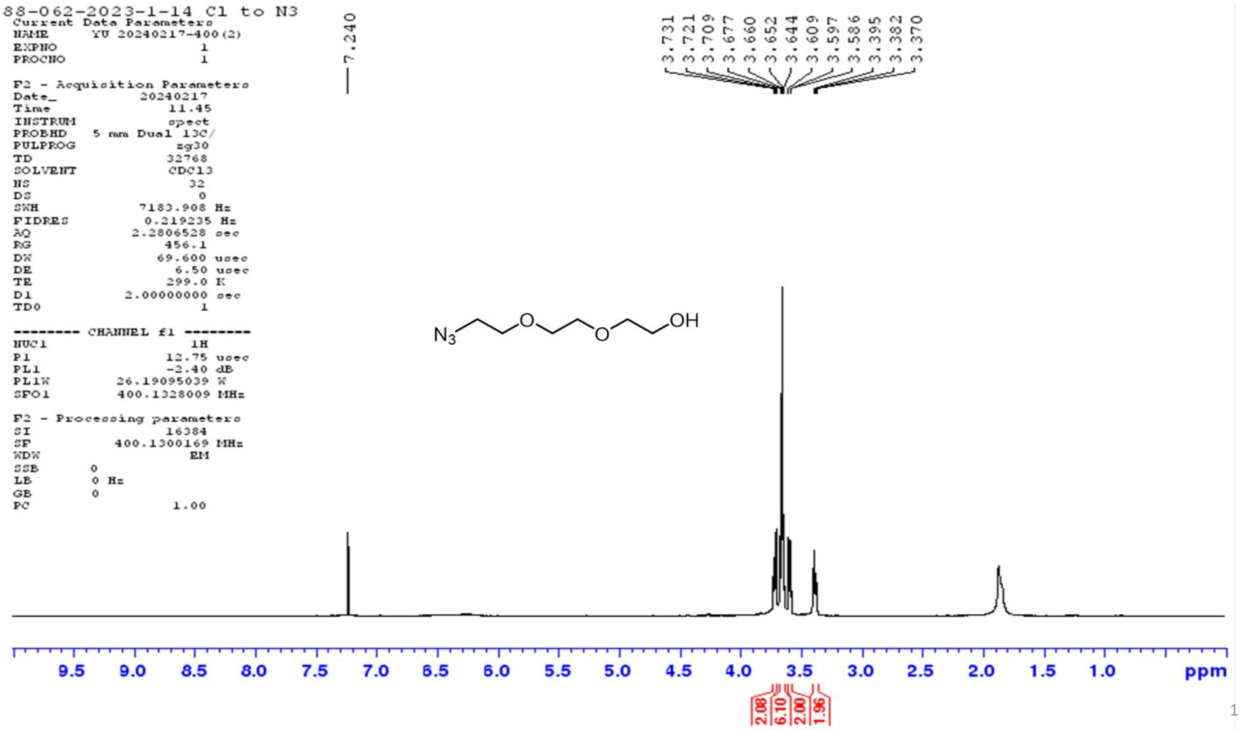

^{1}H NMR Spectrum of Compound **3** (400 MHz, $CDCl_3$)

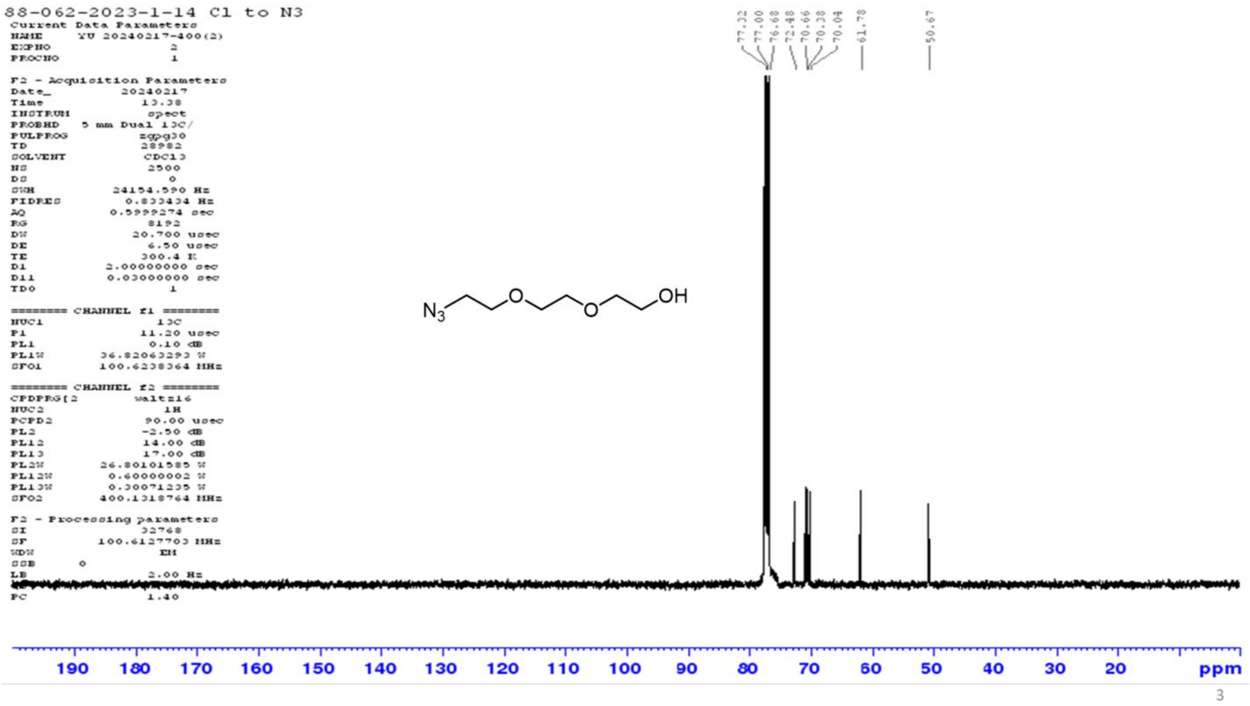

^{13}C NMR Spectrum of Compound **3** (100 MHz, $CDCl_3$)

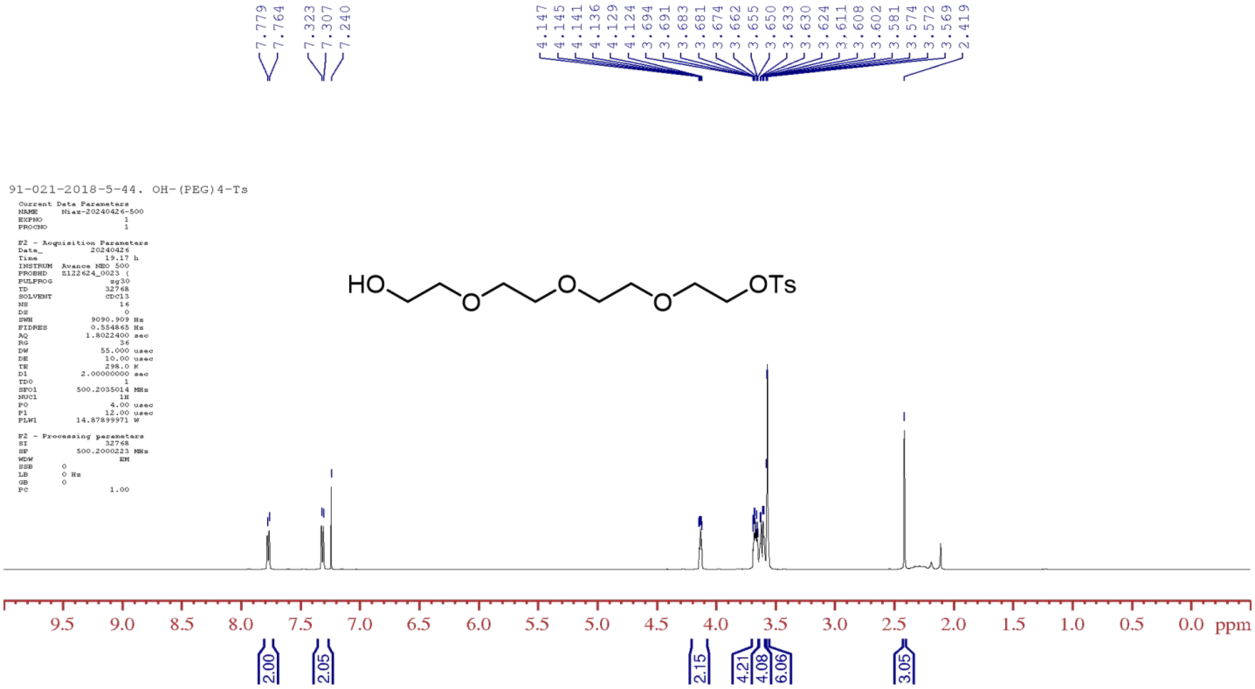

^{1}H NMR Spectrum of Compound **4** (500 MHz, $CDCl_3$)

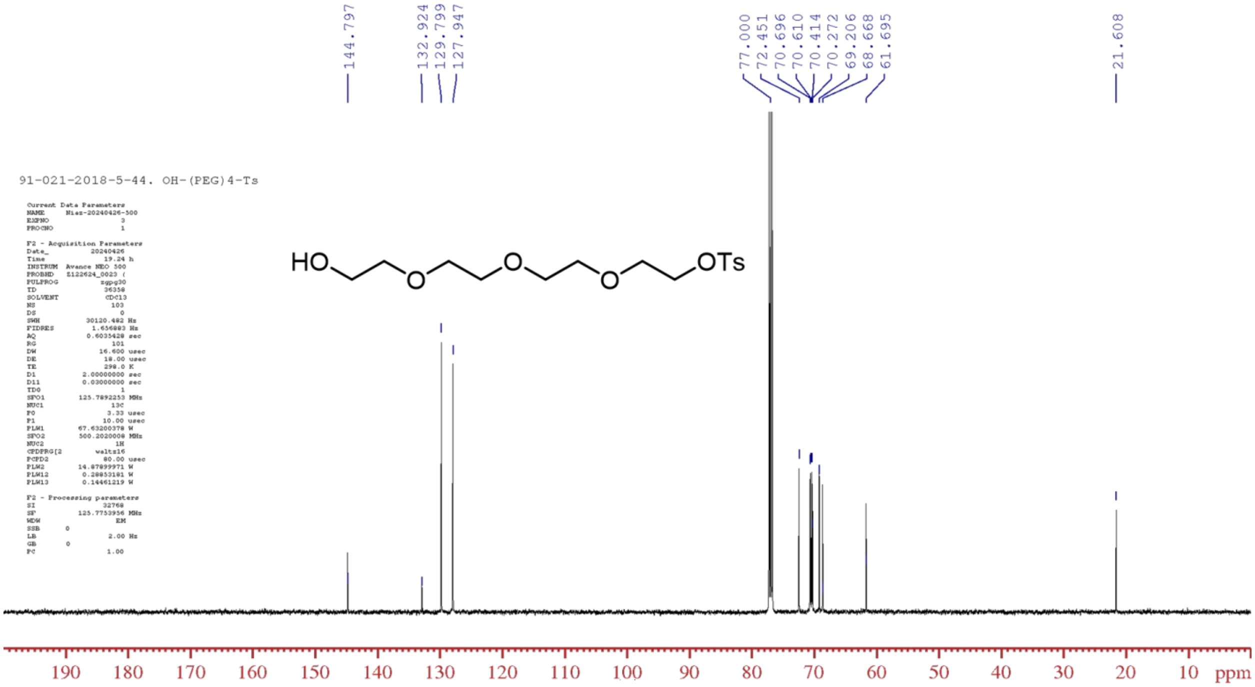

^{13}C NMR Spectrum of Compound **4** (125 MHz, $CDCl_3$)

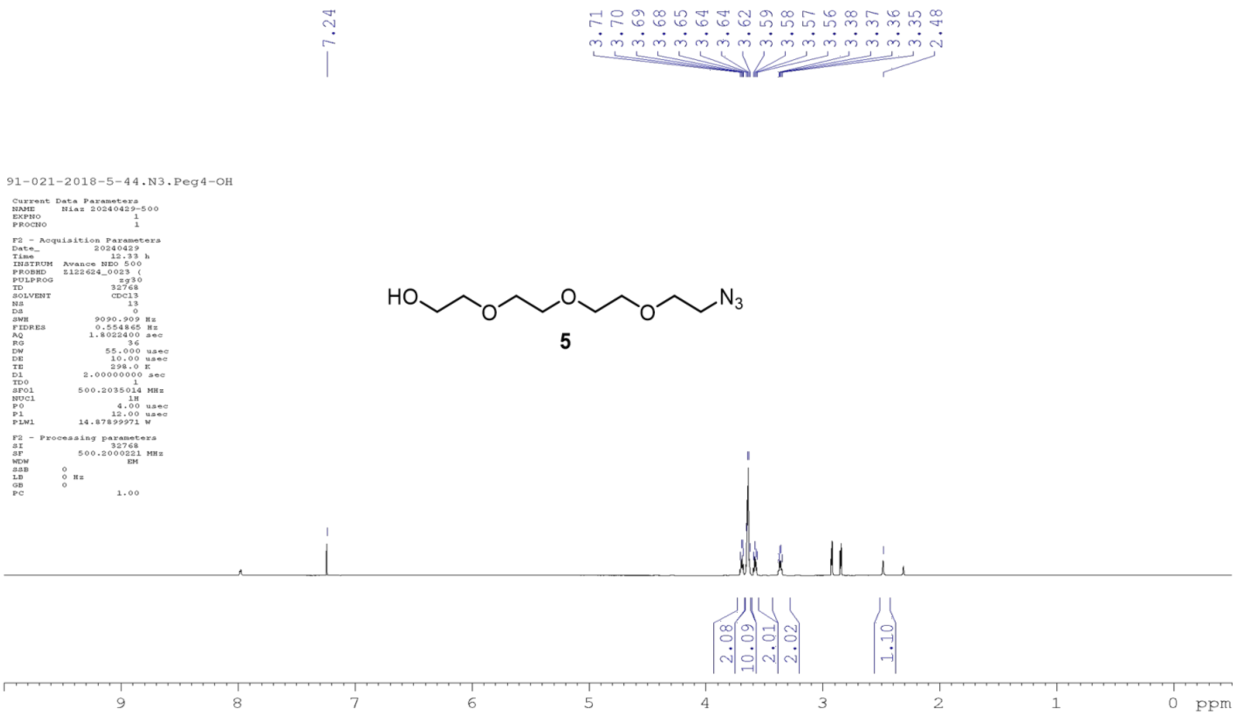

^{1}H NMR Spectrum of Compound **5** (500 MHz, $CDCl_3$)

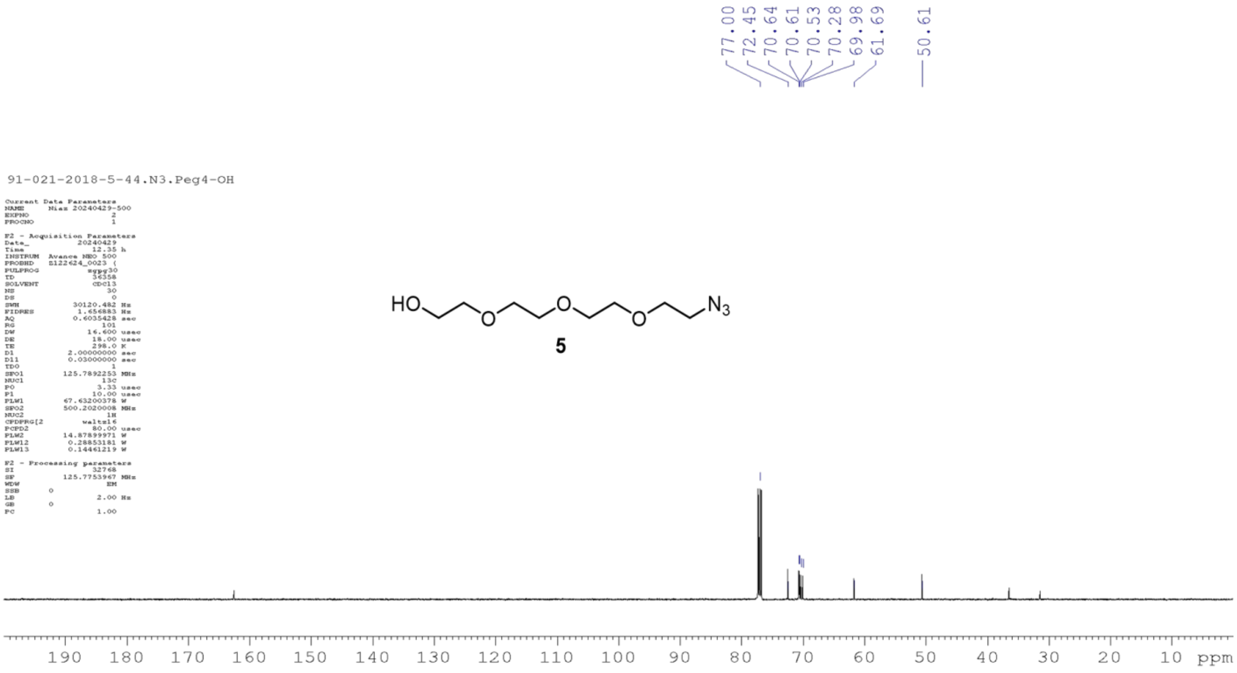

^{13}C NMR Spectrum of Compound **5** (125 MHz, $CDCl_3$)

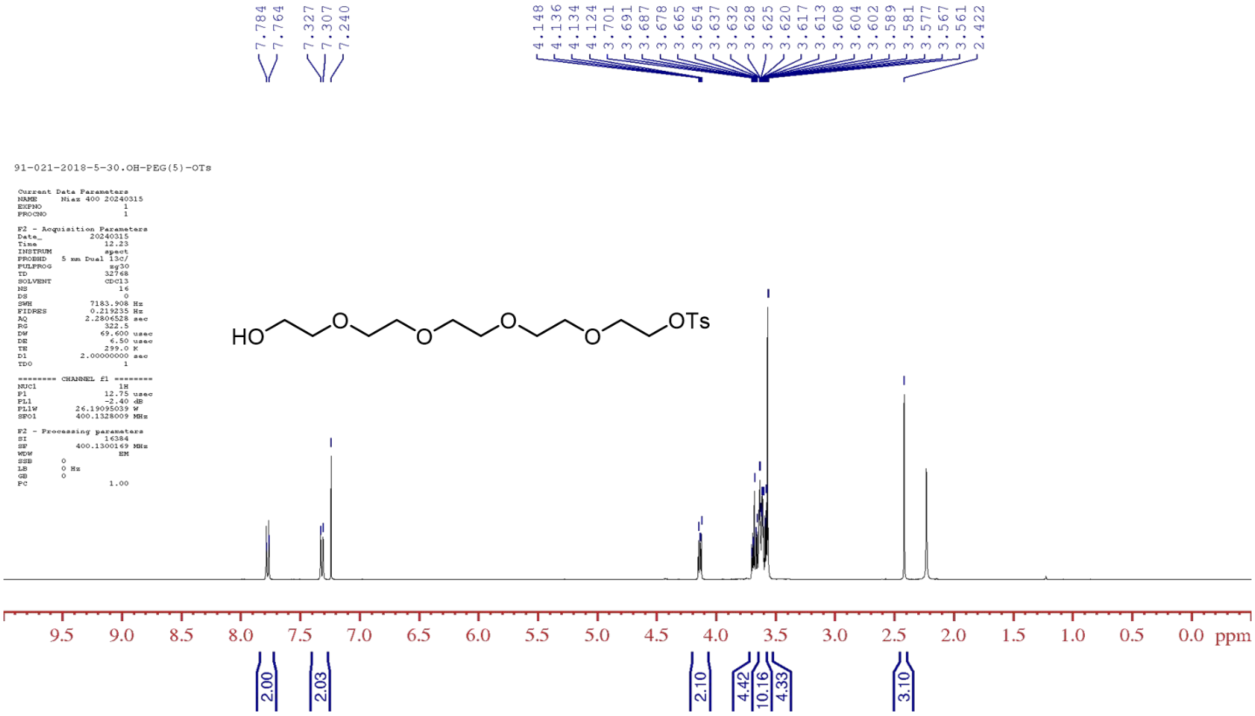

^{1}H NMR Spectrum of Compound **6** (400 MHz, $CDCl_3$)

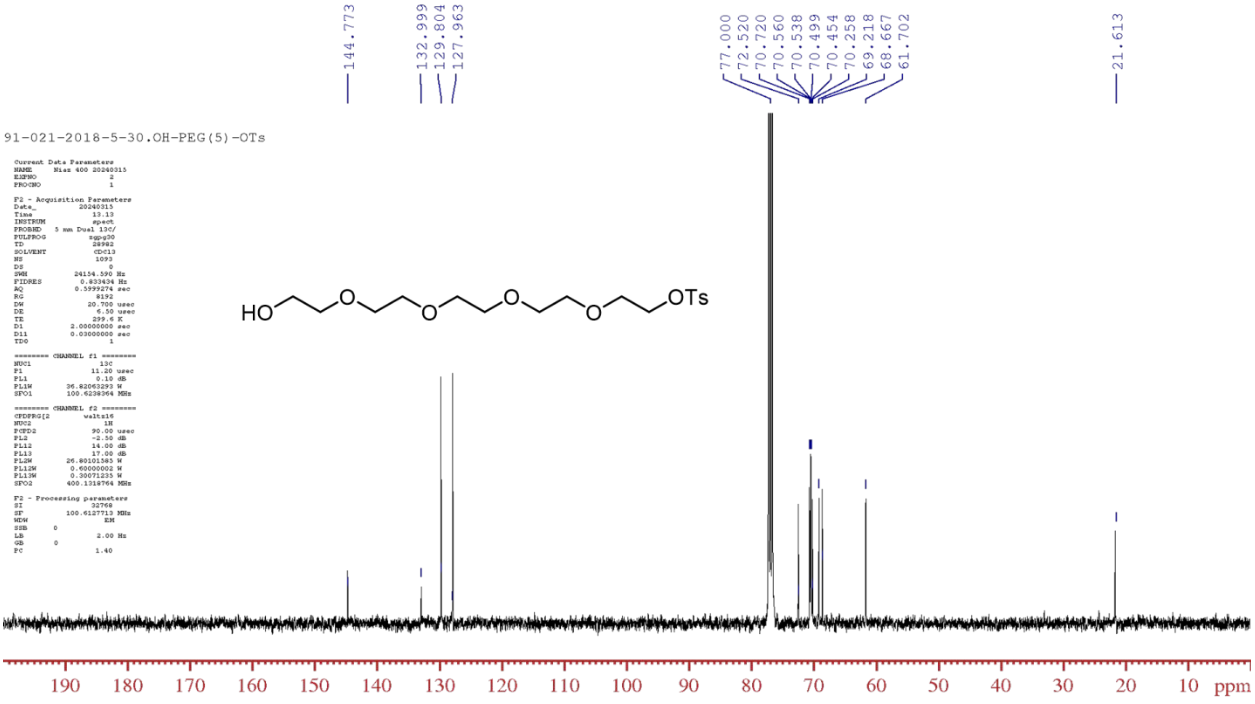

^{13}C NMR Spectrum of Compound **6** (100 MHz, $CDCl_3$)

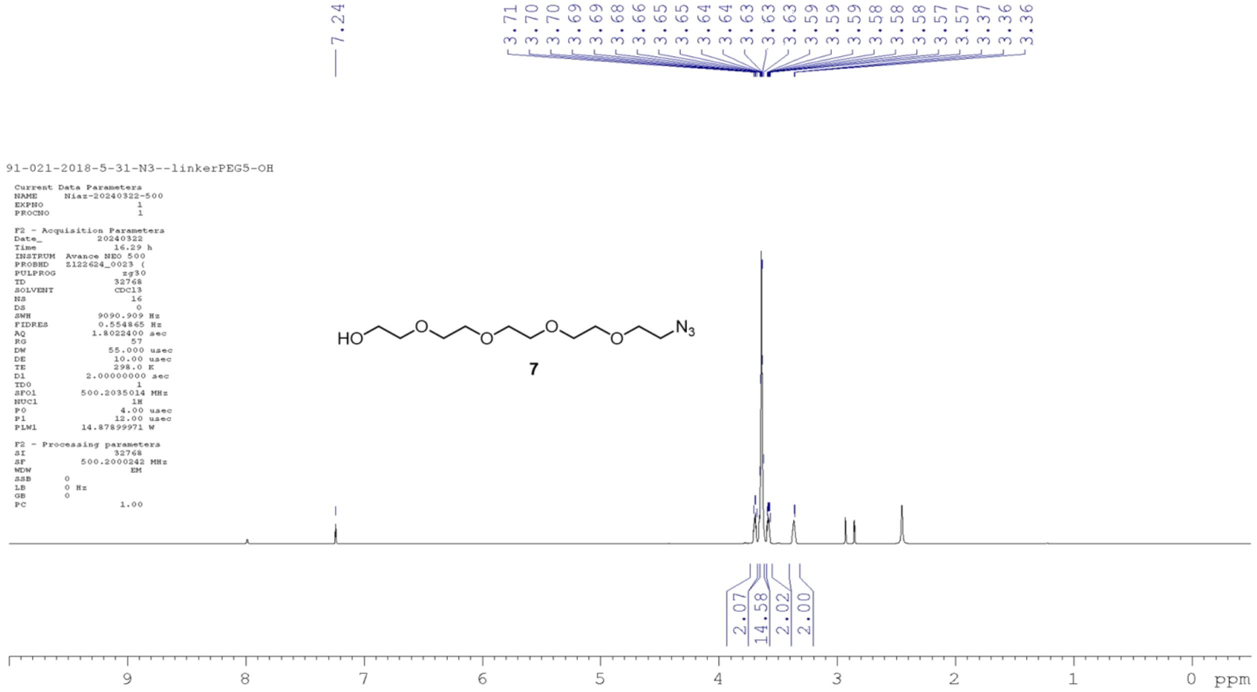

^{1}H NMR Spectrum of Compound **7** (500 MHz, $CDCl_3$)

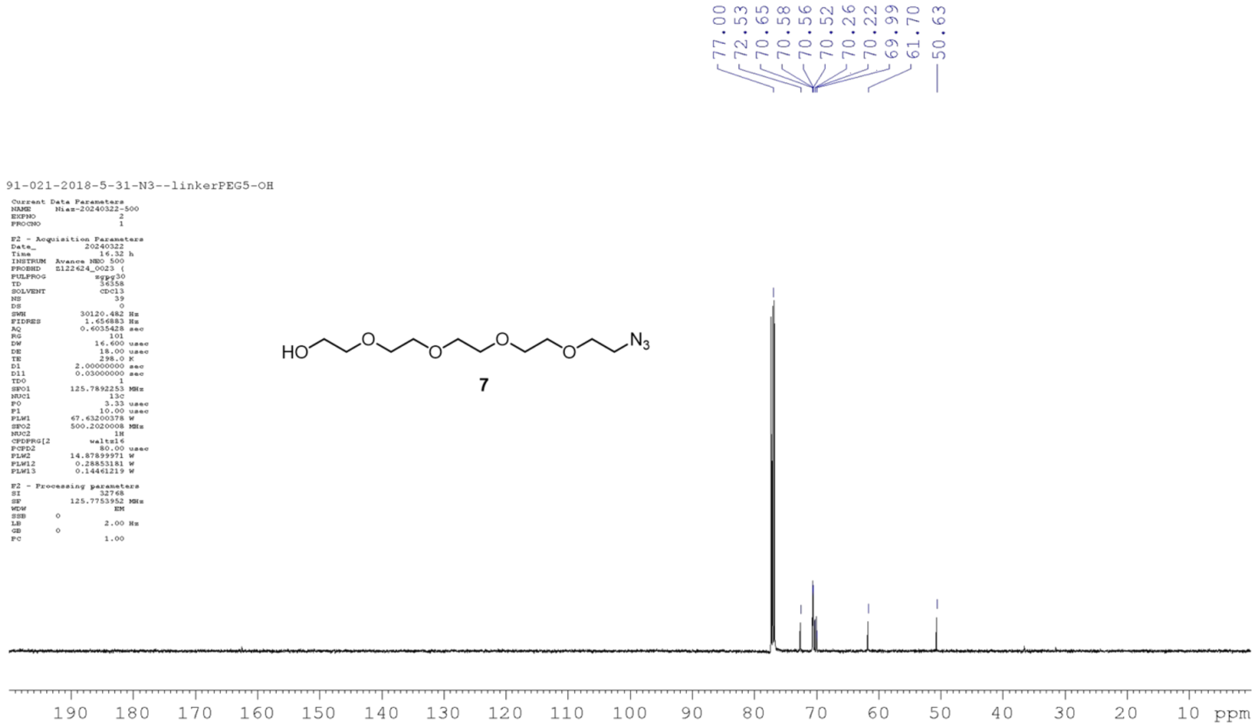

^{13}C NMR Spectrum of Compound **7** (125 MHz, $CDCl_3$)

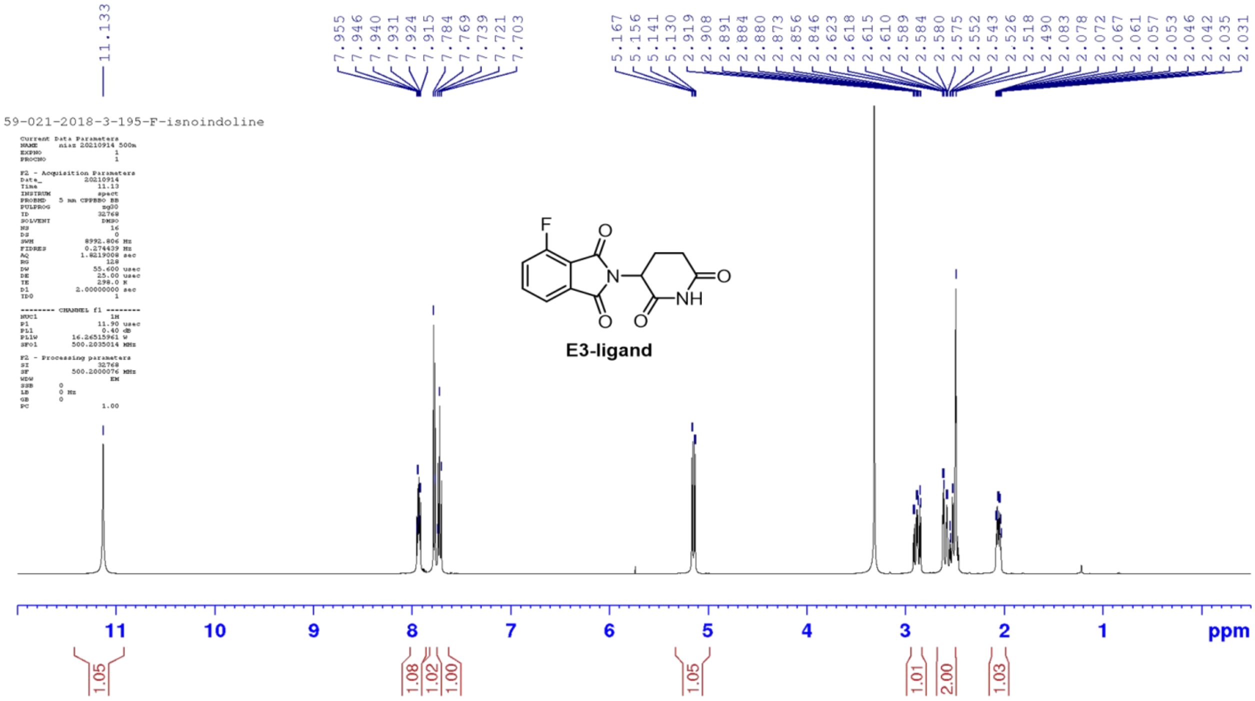

^{1}H NMR Spectrum of Compound **E3-ligand** (500 MHz, DMSO-d_6)

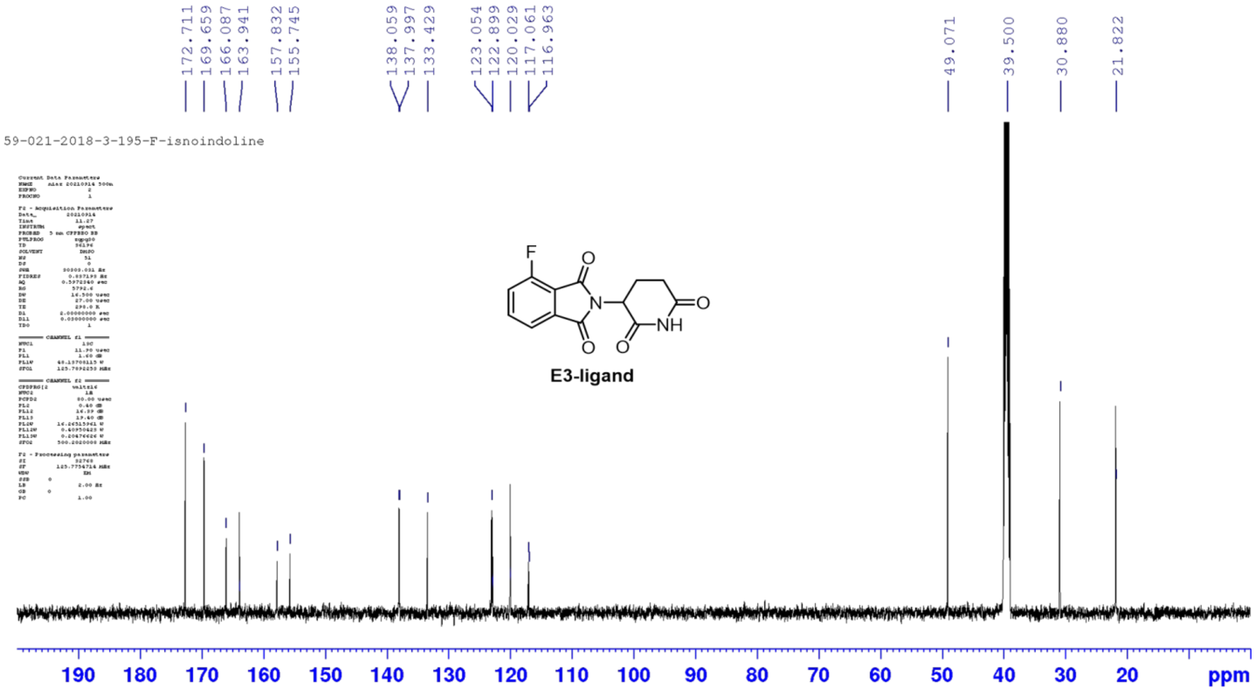

^{13}C NMR Spectrum of Compound **E3-ligand** (125 MHz, DMSO-d_6)

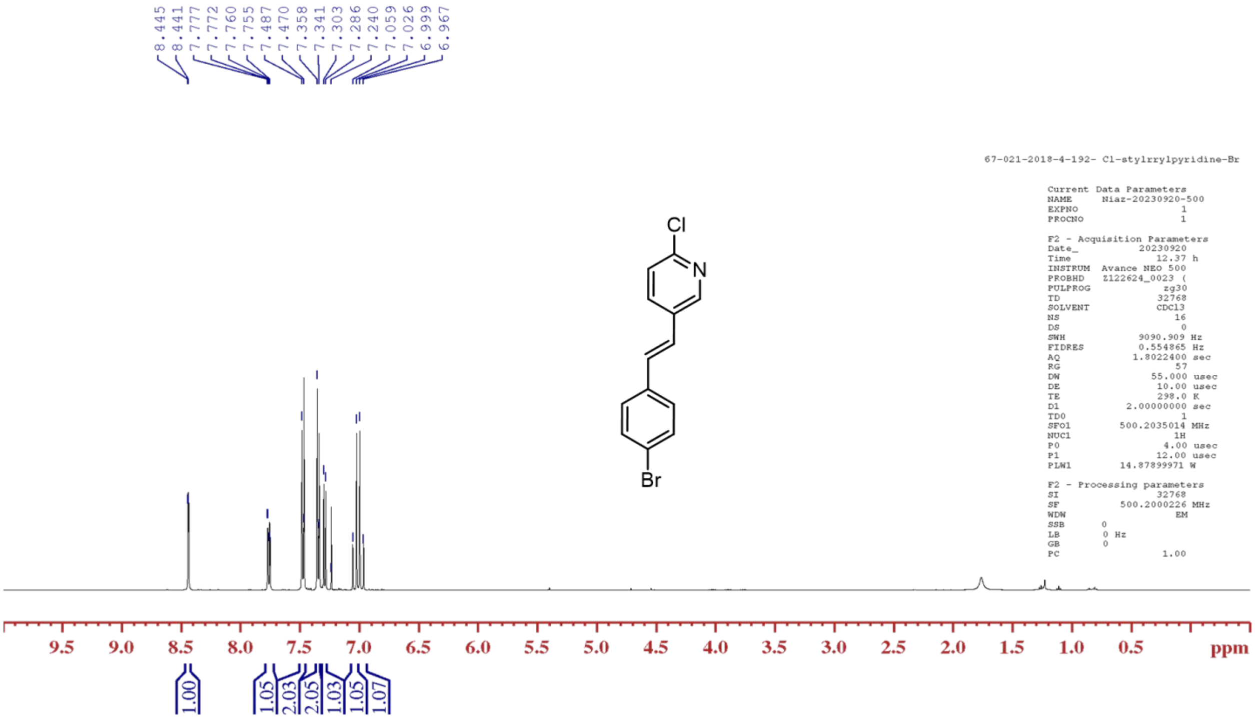

^{1}H NMR Spectrum of Compound **8** (500 MHz, $CDCl_3$)

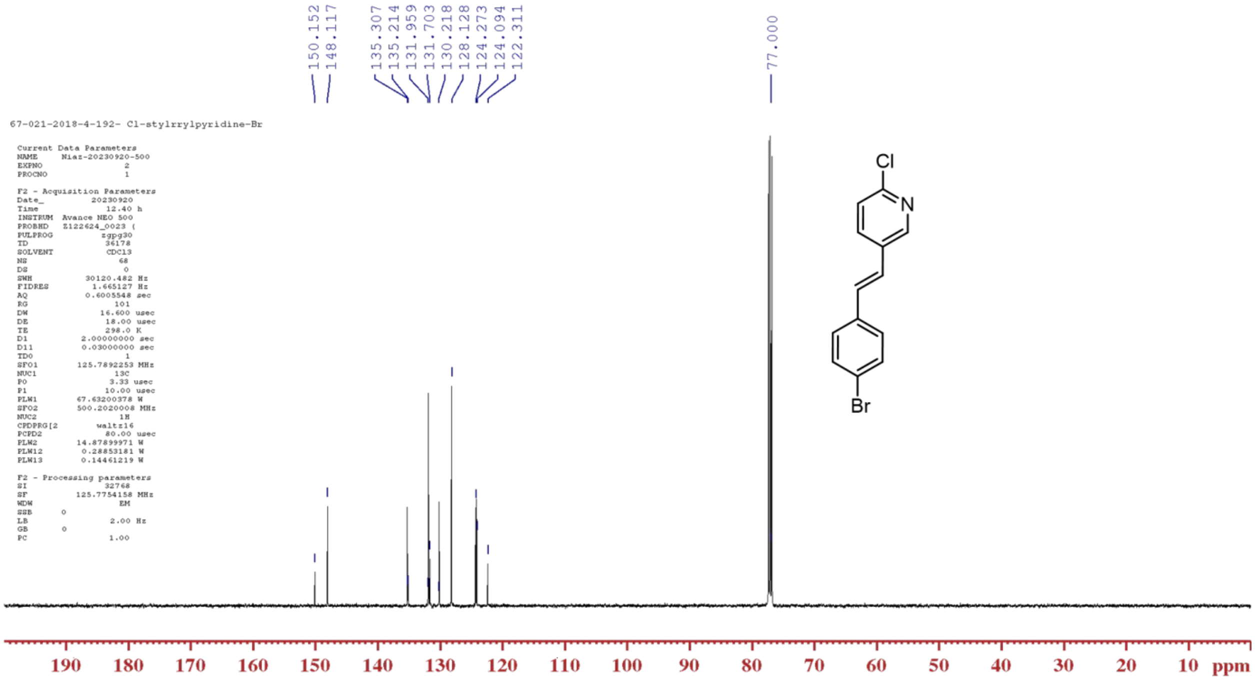

^{13}C NMR Spectrum of Compound **8** (125 MHz, $CDCl_3$)

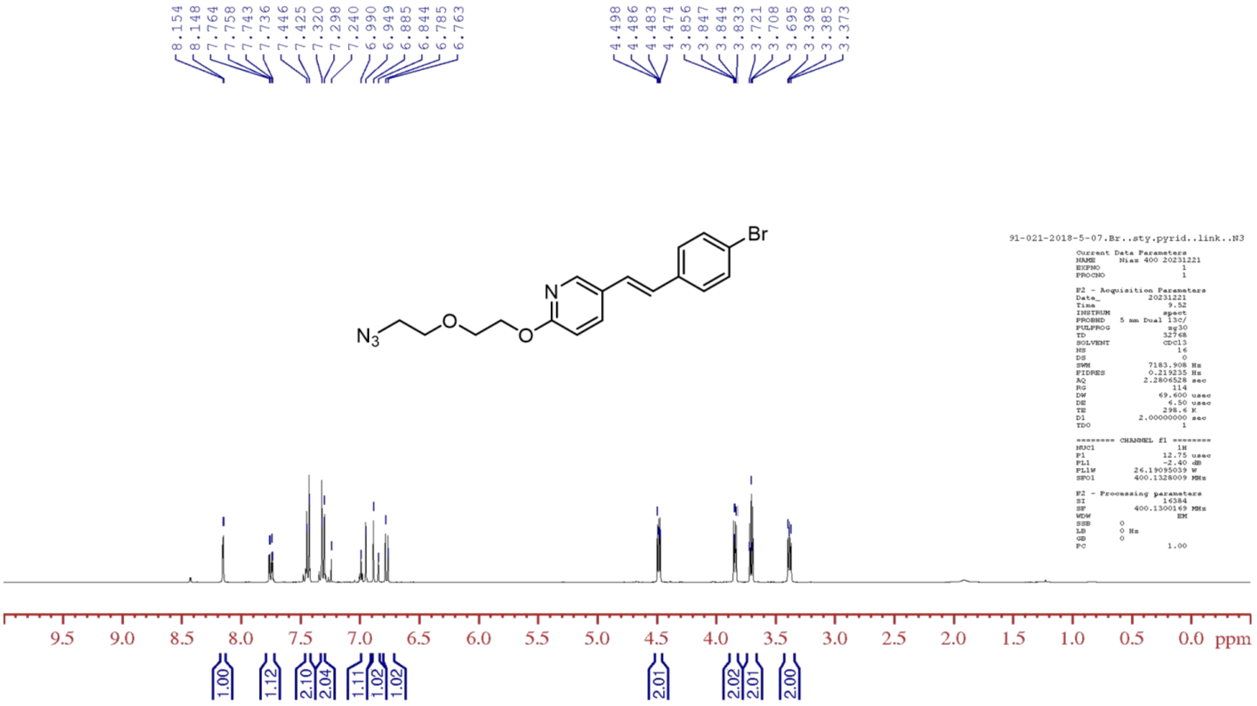

^{1}H NMR Spectrum of Compound **9** (400 MHz, $CDCl_3$)

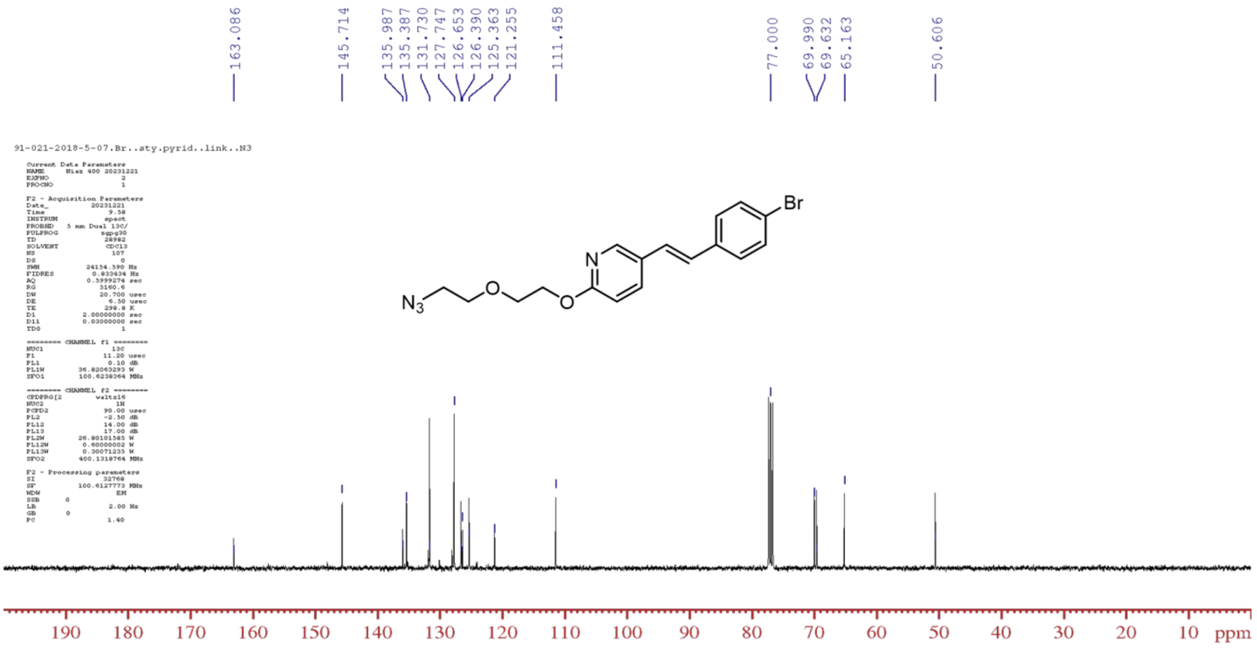

^{13}C NMR Spectrum of Compound **9** (100 MHz, $CDCl_3$)

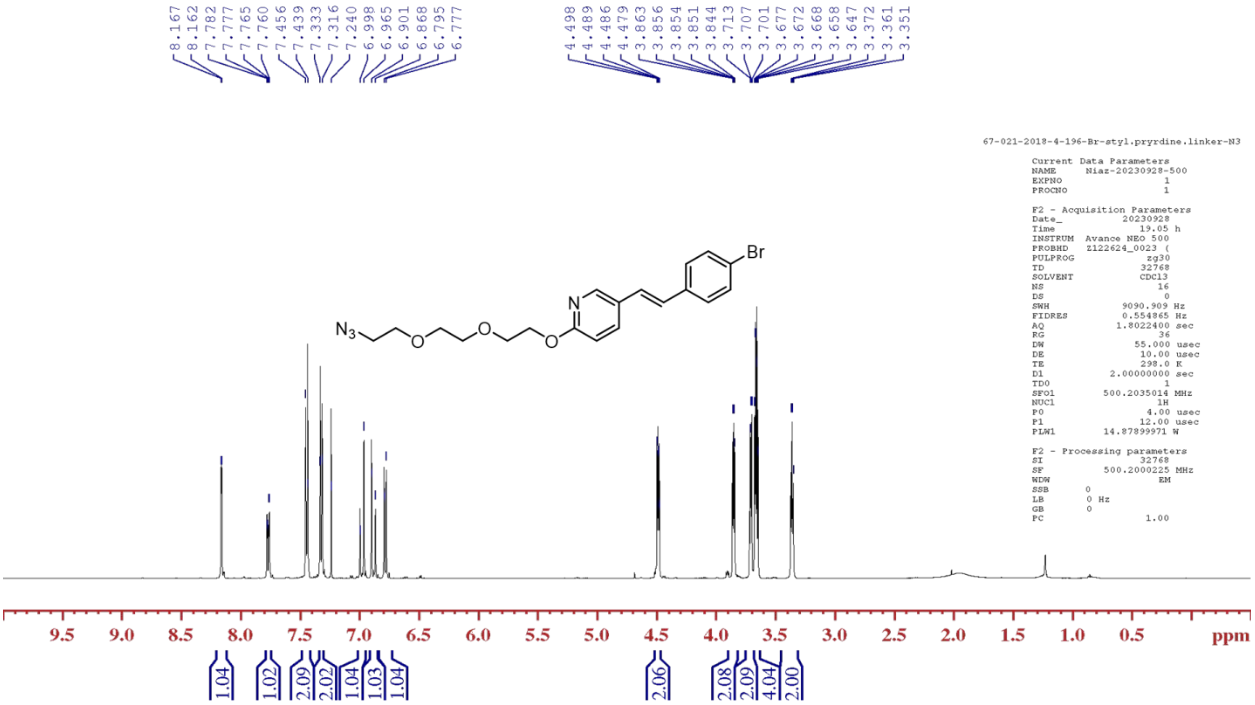

^{1}H NMR Spectrum of Compound **10** (500 MHz, $CDCl_3$)

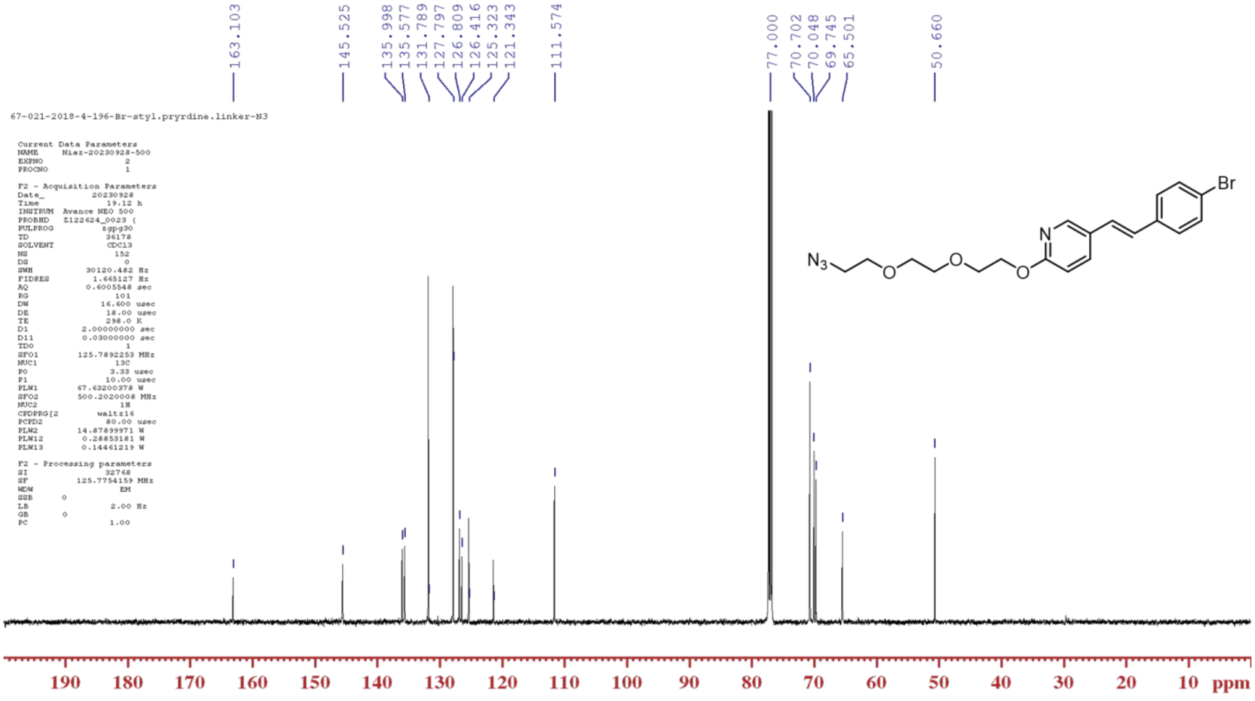

^{13}C NMR Spectrum of Compound **10** (125 MHz, $CDCl_3$)

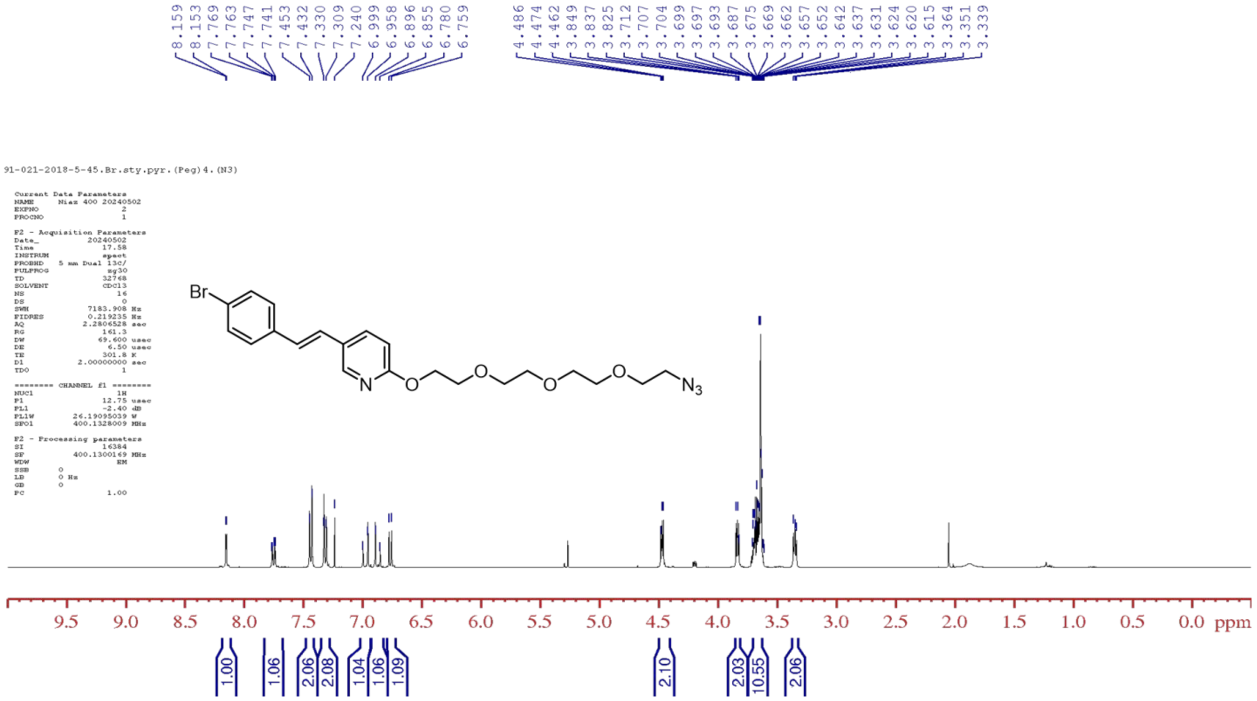

^{1}H NMR Spectrum of Compound **11** (400 MHz, $CDCl_3$)

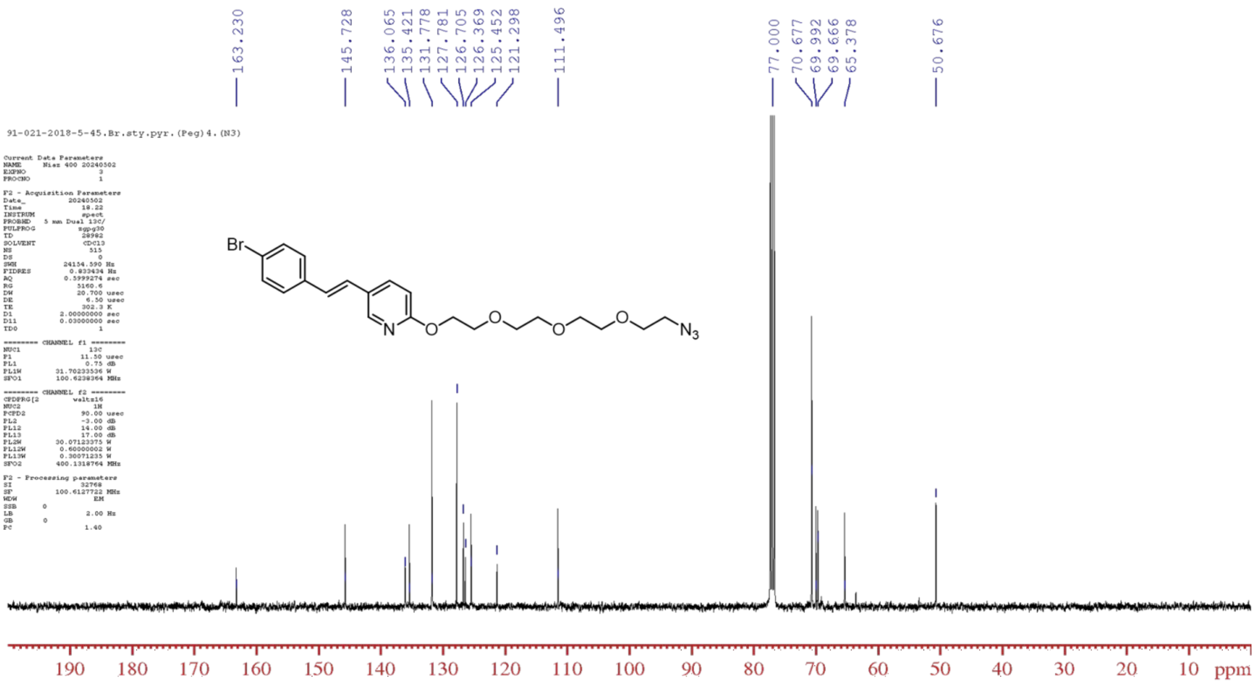

^{13}C NMR Spectrum of Compound **11** (100 MHz, $CDCl_3$)

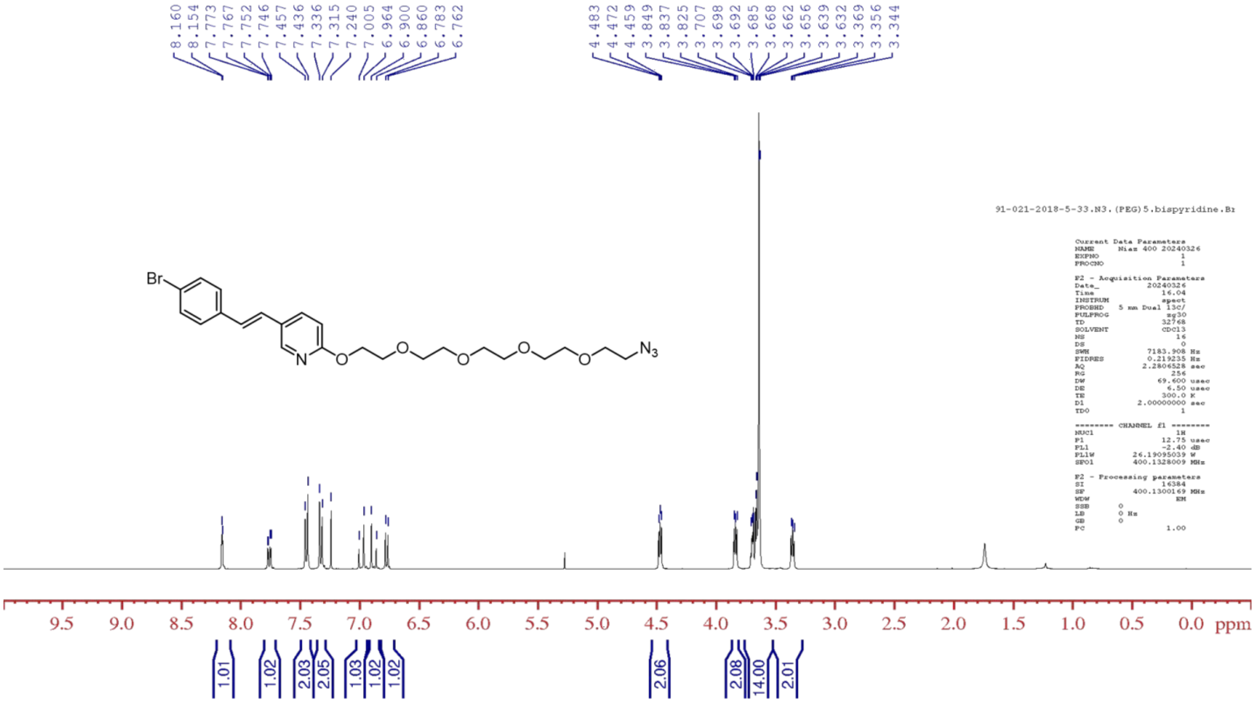

^{1}H NMR Spectrum of Compound **12** (100 MHz, $CDCl_3$)

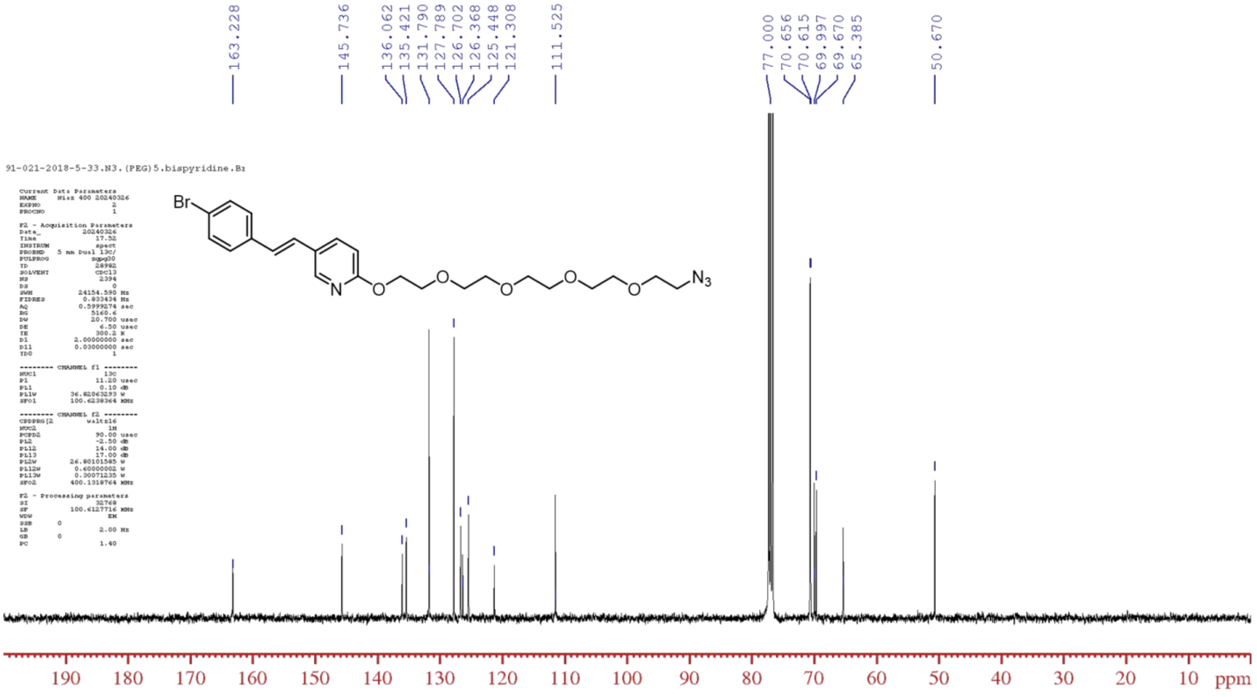

^{13}C NMR Spectrum of Compound **12** (100 MHz, $CDCl_3$)

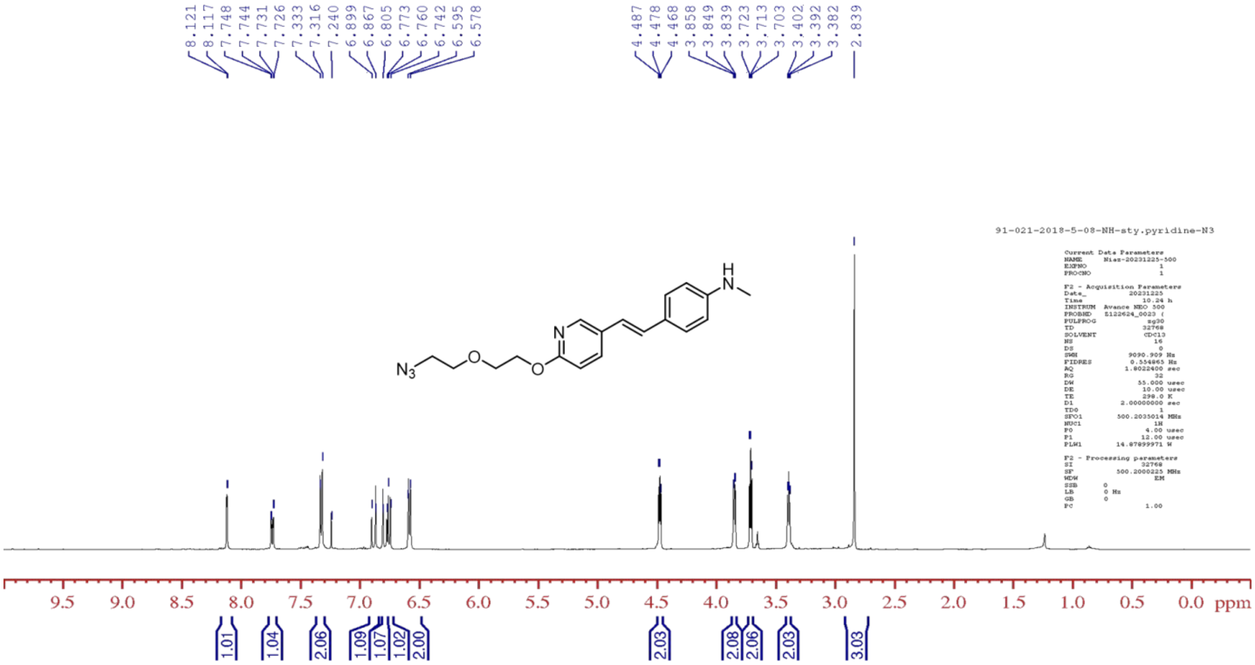

^{1}H NMR Spectrum of Compound **13** (500 MHz, $CDCl_3$)

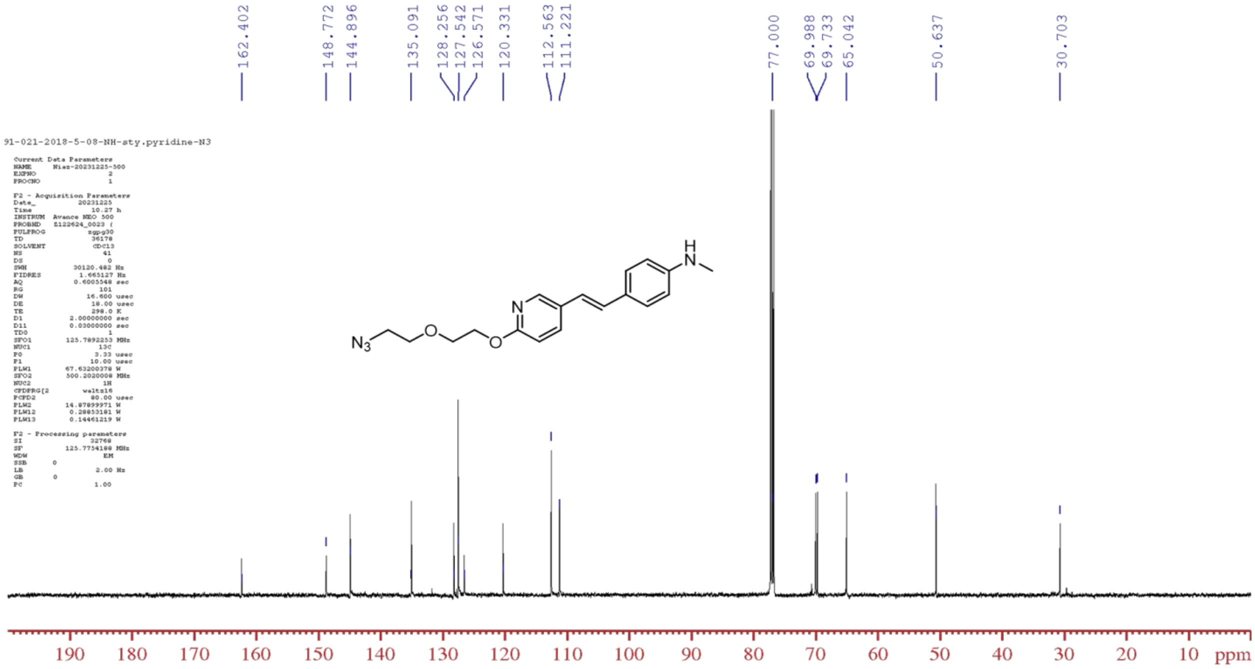

^{13}C NMR Spectrum of Compound **13** (125 MHz, $CDCl_3$)

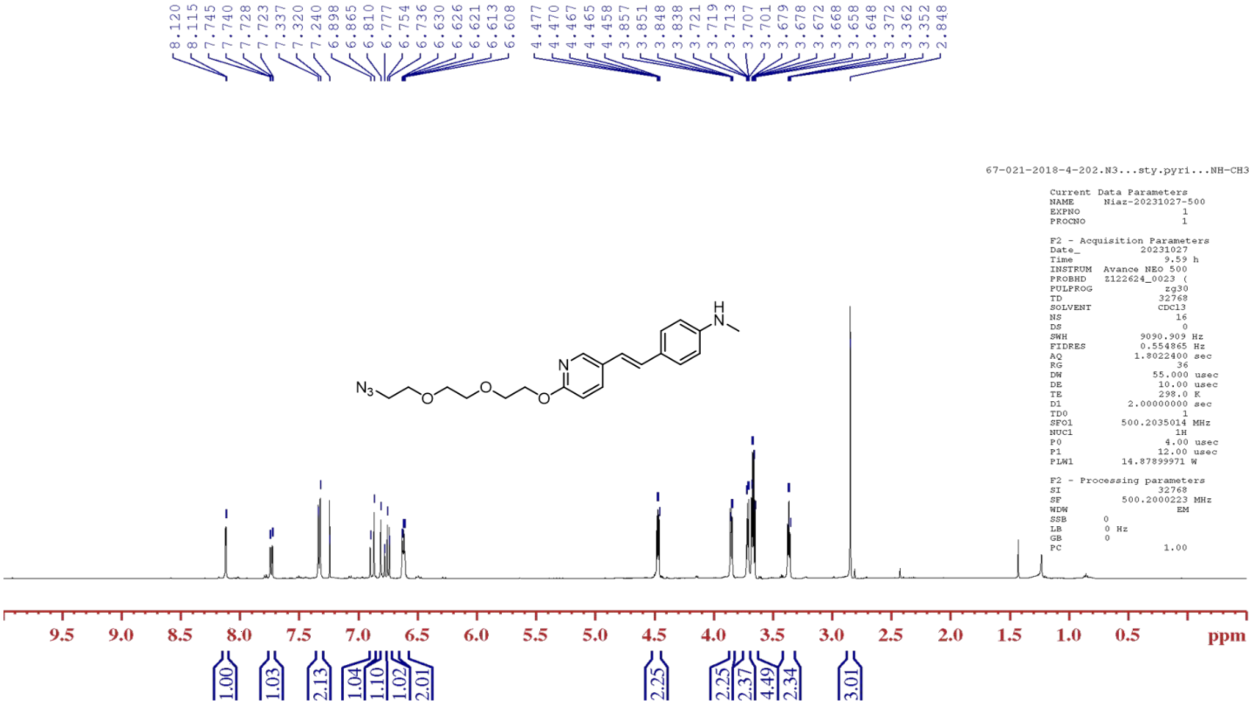

^{1}H NMR Spectrum of Compound **14** (500 MHz, $CDCl_3$)

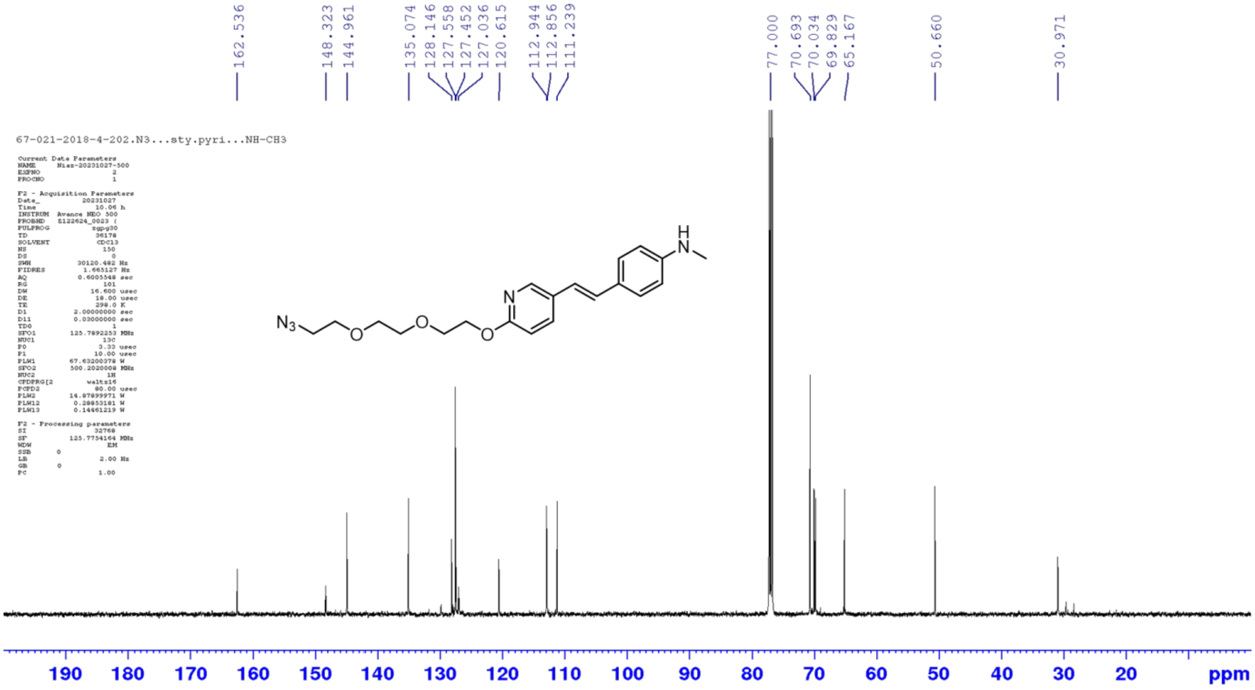

^{13}C NMR Spectrum of Compound **14** (125 MHz, $CDCl_3$)

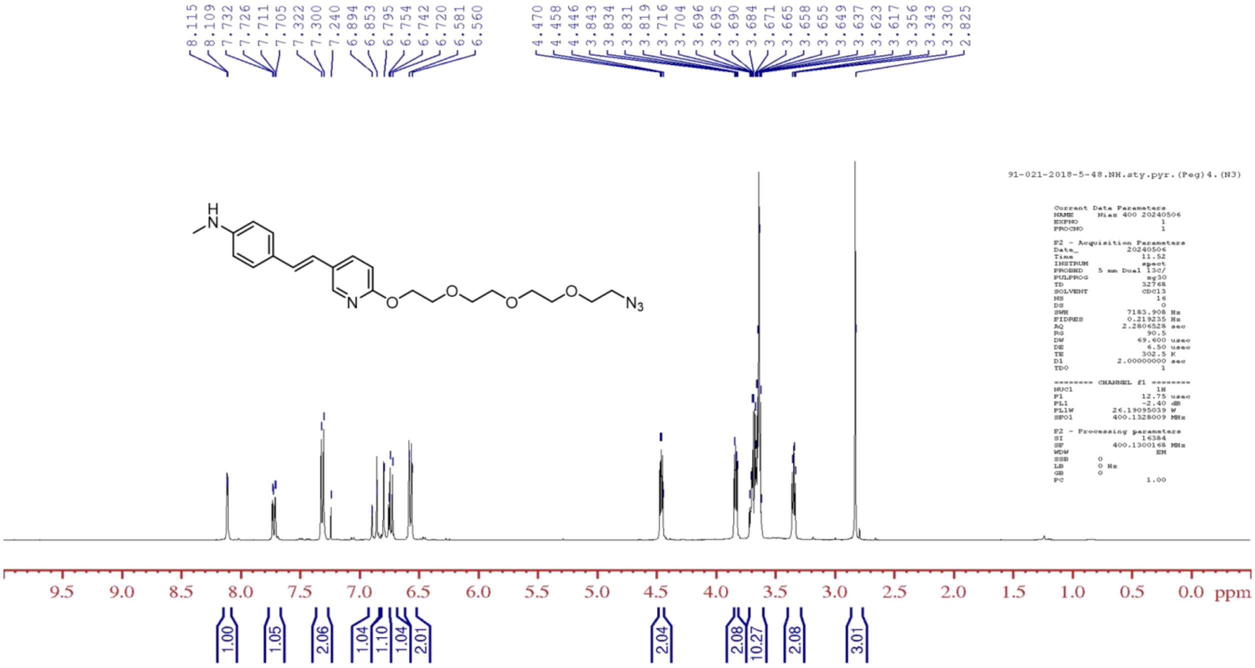

^{1}H NMR Spectrum of Compound **15** (400 MHz, $CDCl_3$)

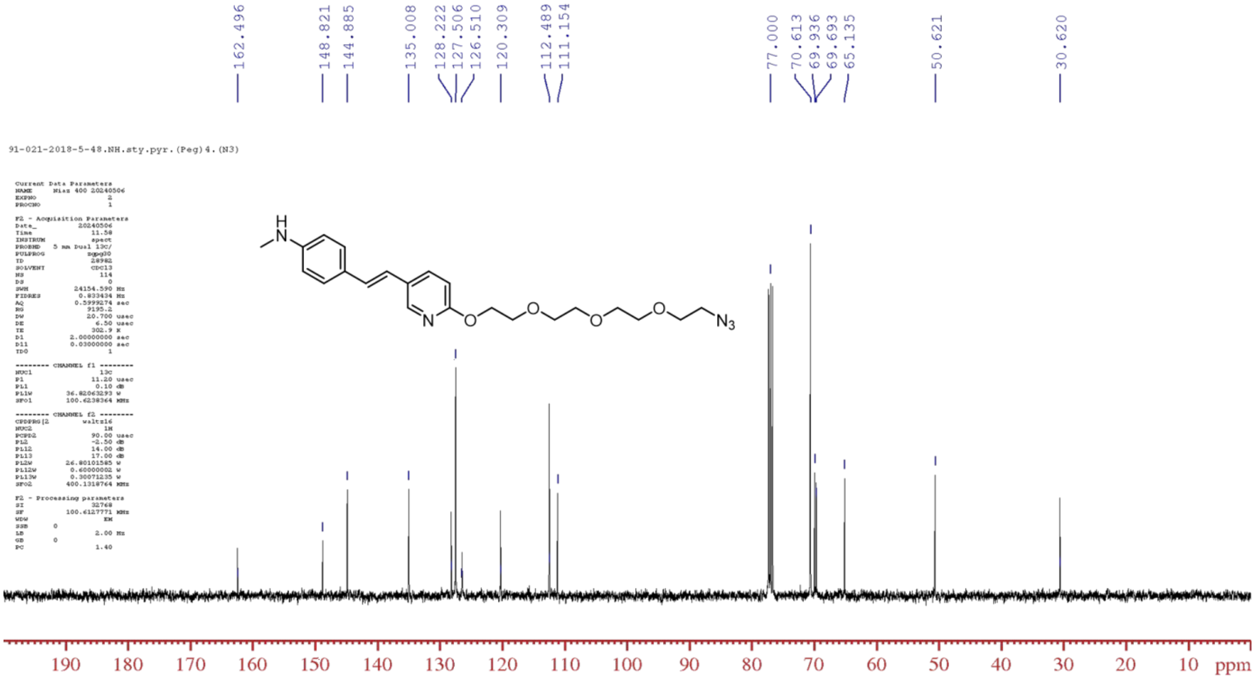

^{13}C NMR Spectrum of Compound **15** (100 MHz, $CDCl_3$)

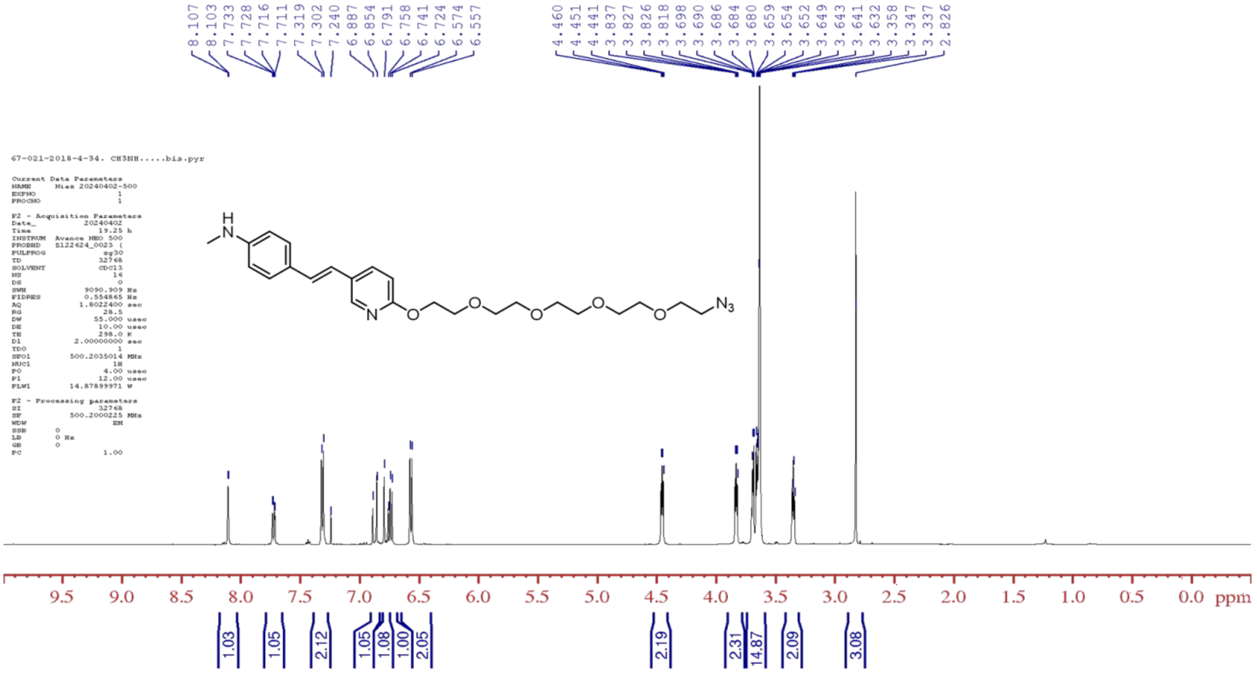

^{1}H NMR Spectrum of Compound **16** (500 MHz, $CDCl_3$)

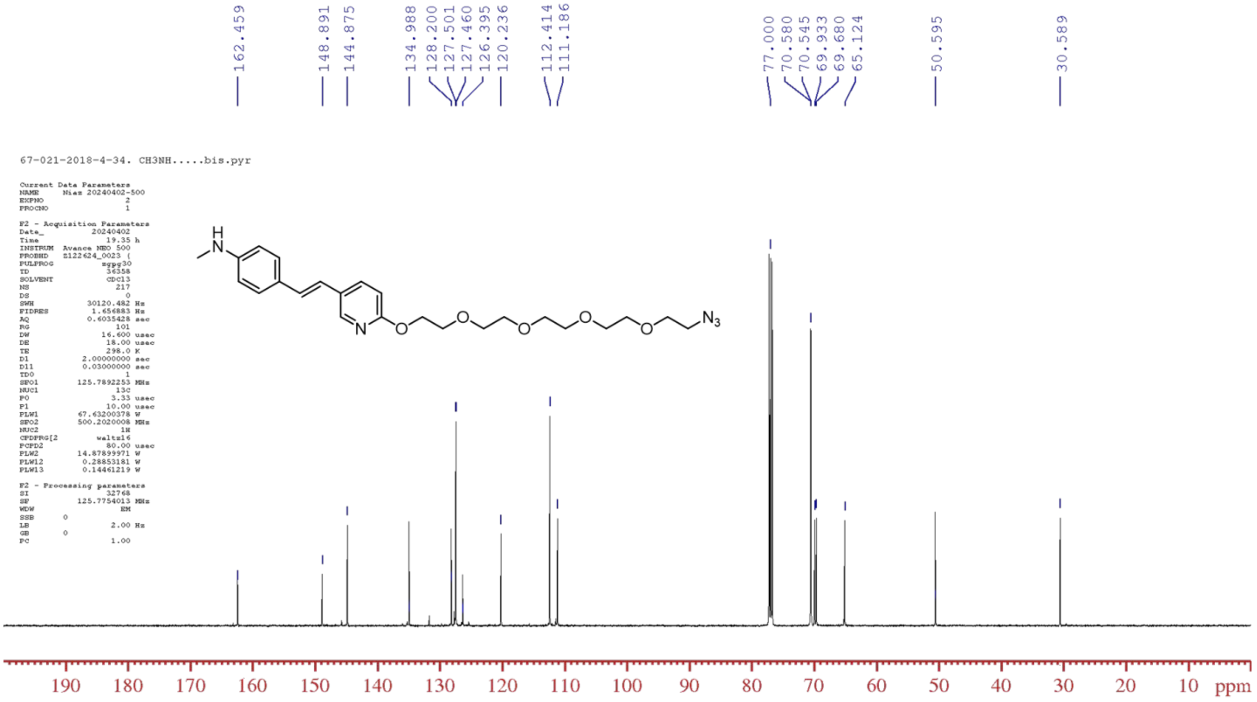

^{13}C NMR Spectrum of Compound **16** (125 MHz, $CDCl_3$)

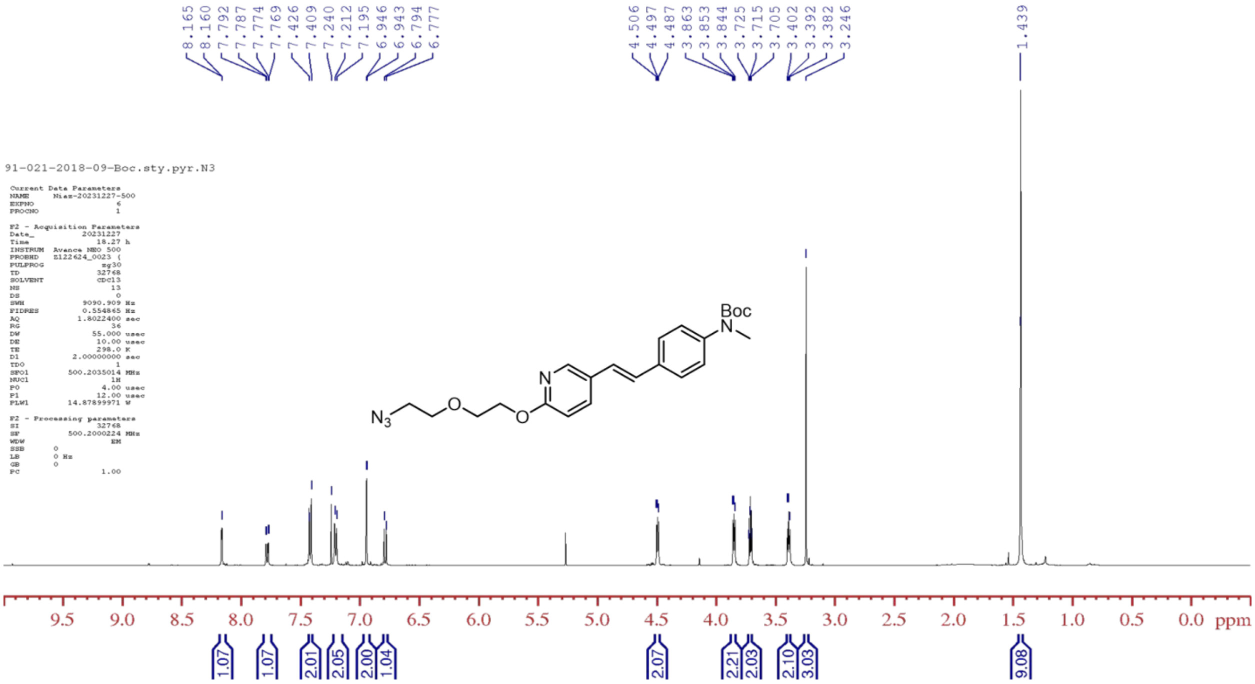

^{1}H NMR Spectrum of Compound **17** (500 MHz, $CDCl_3$)

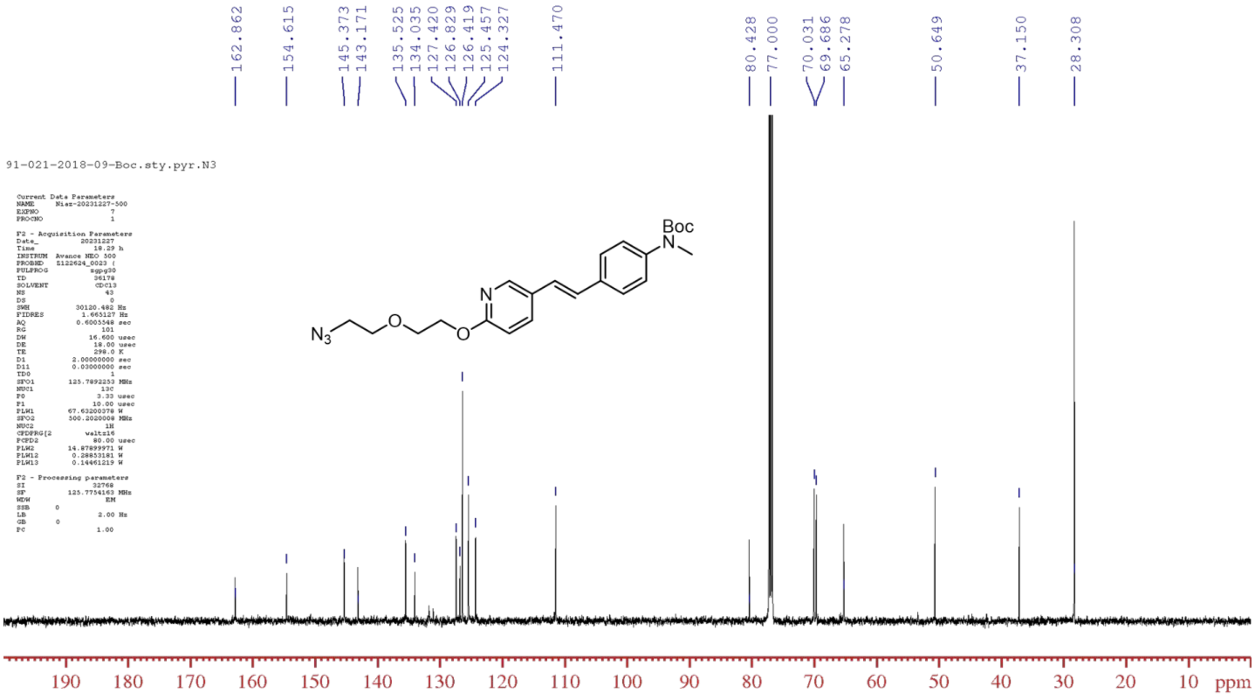

^{13}C NMR Spectrum of Compound **17** (125 MHz, $CDCl_3$)

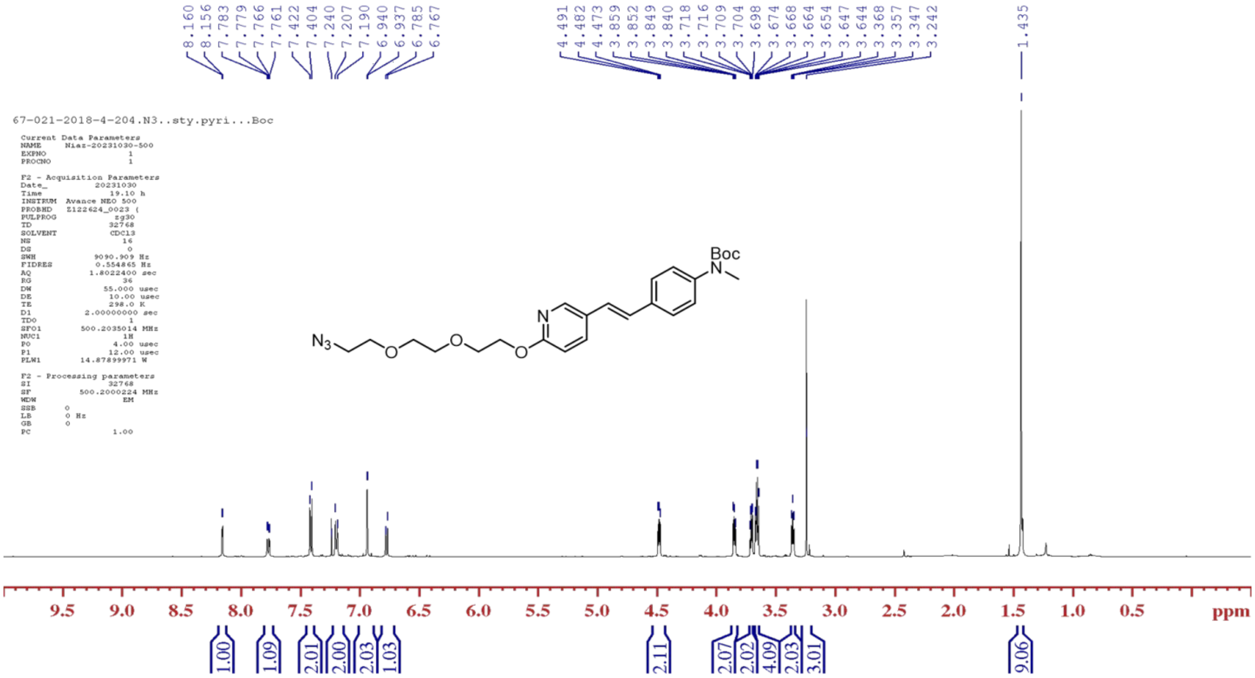

^{1}H NMR Spectrum of Compound **18** (500 MHz, $CDCl_3$)

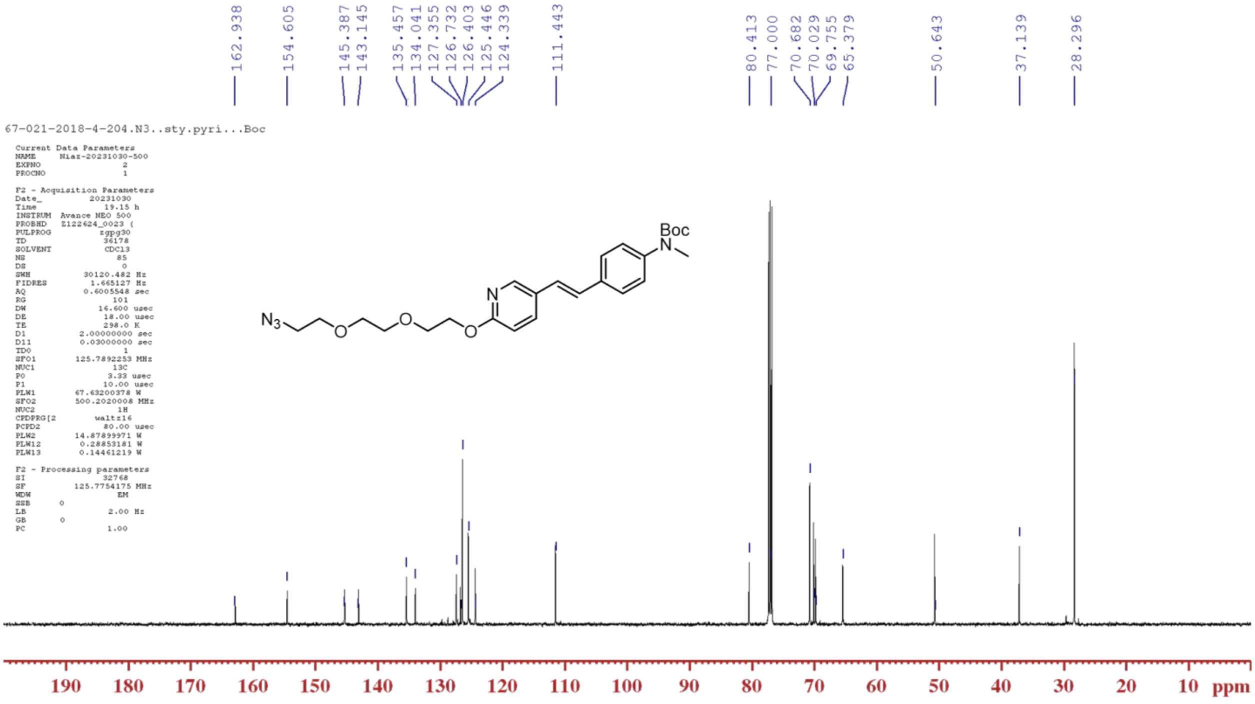

^{13}C NMR Spectrum of Compound **18** (125 MHz, $CDCl_3$)

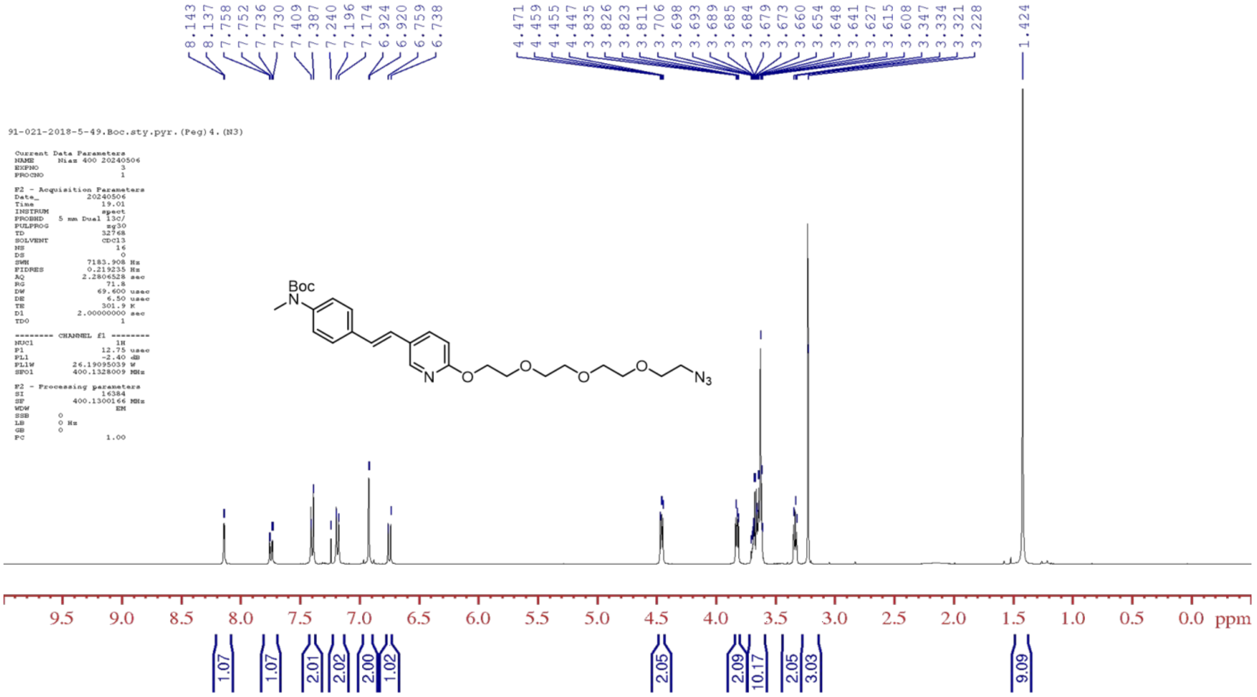

^{1}H NMR Spectrum of Compound **19** (400 MHz, $CDCl_3$)

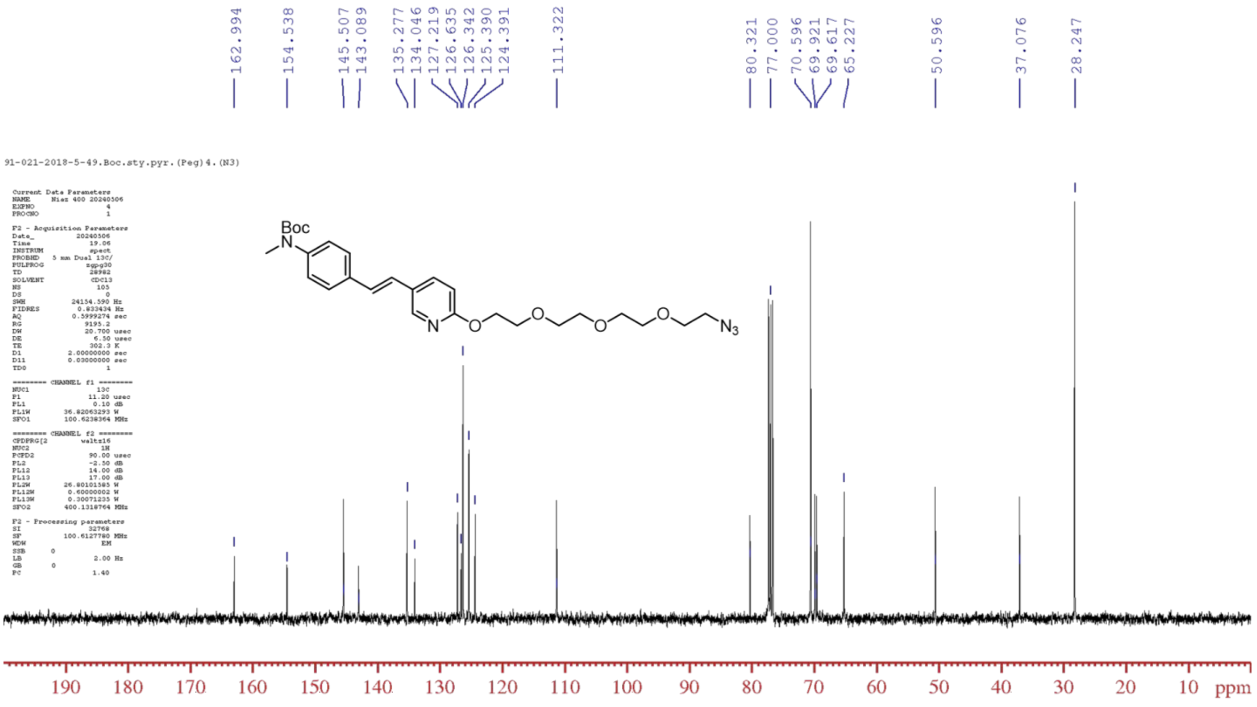

^{13}C NMR Spectrum of Compound **19** (100 MHz, $CDCl_3$)

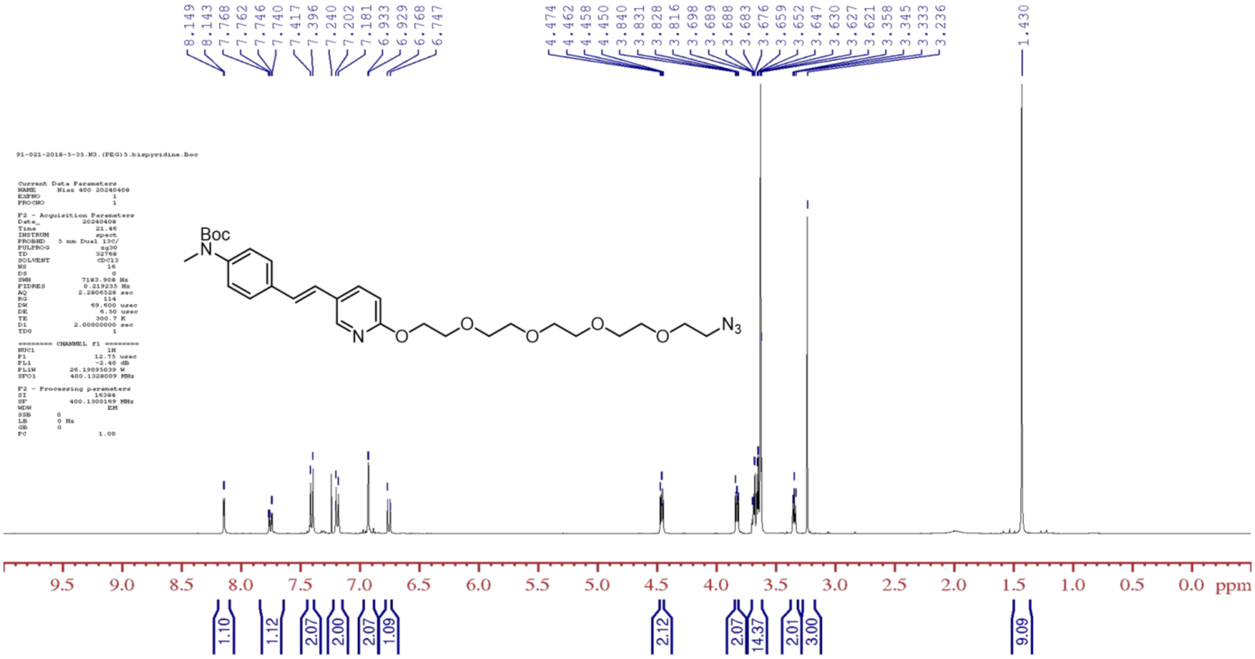

^{1}H NMR Spectrum of Compound **20** (400 MHz, $CDCl_3$)

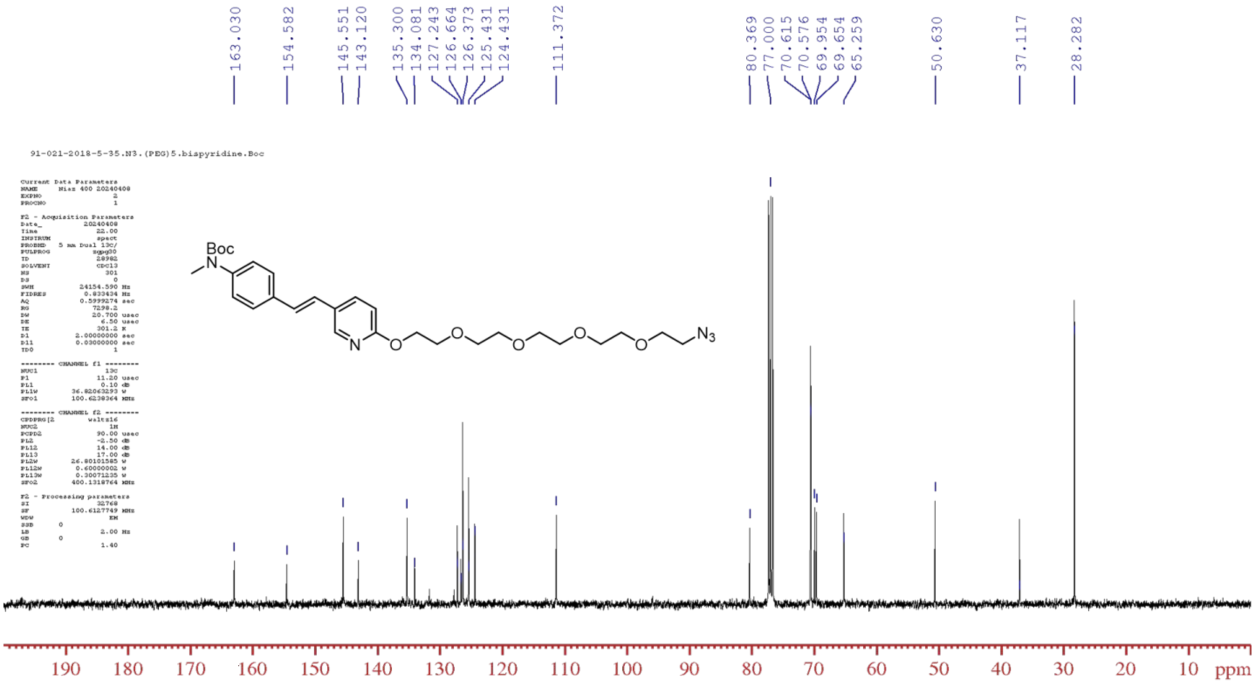

^{13}C NMR Spectrum of Compound **20** (100 MHz, $CDCl_3$)

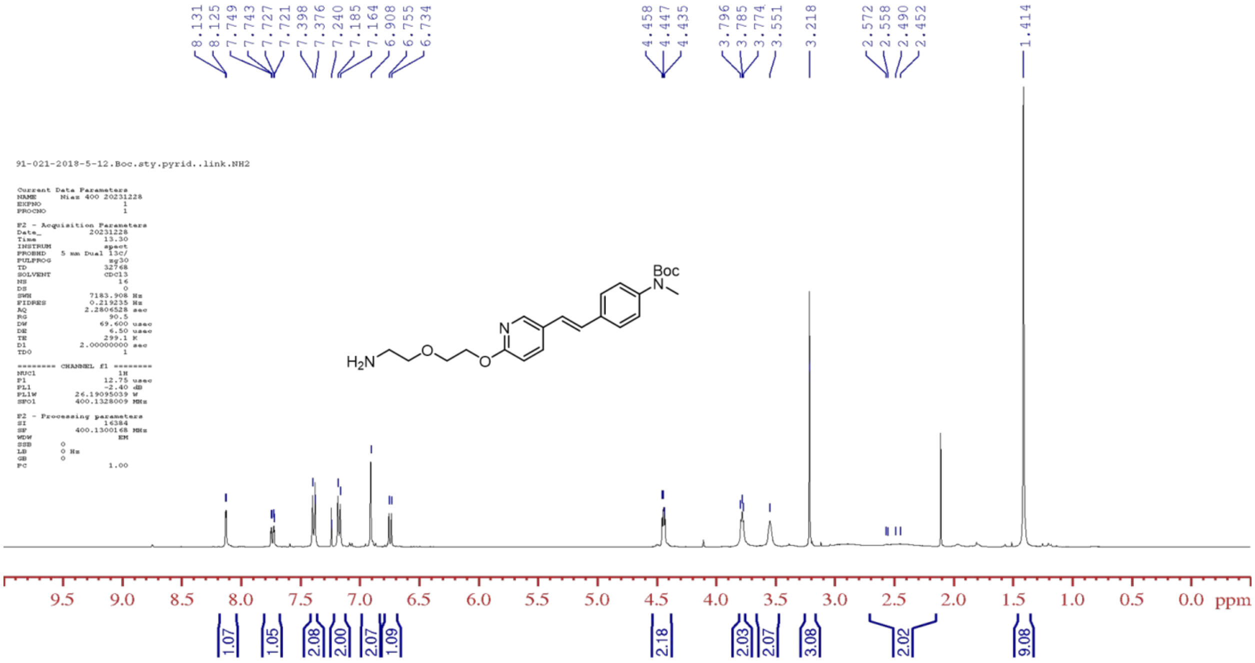

^{1}H NMR Spectrum of Compound **21** (400 MHz, $CDCl_3$)

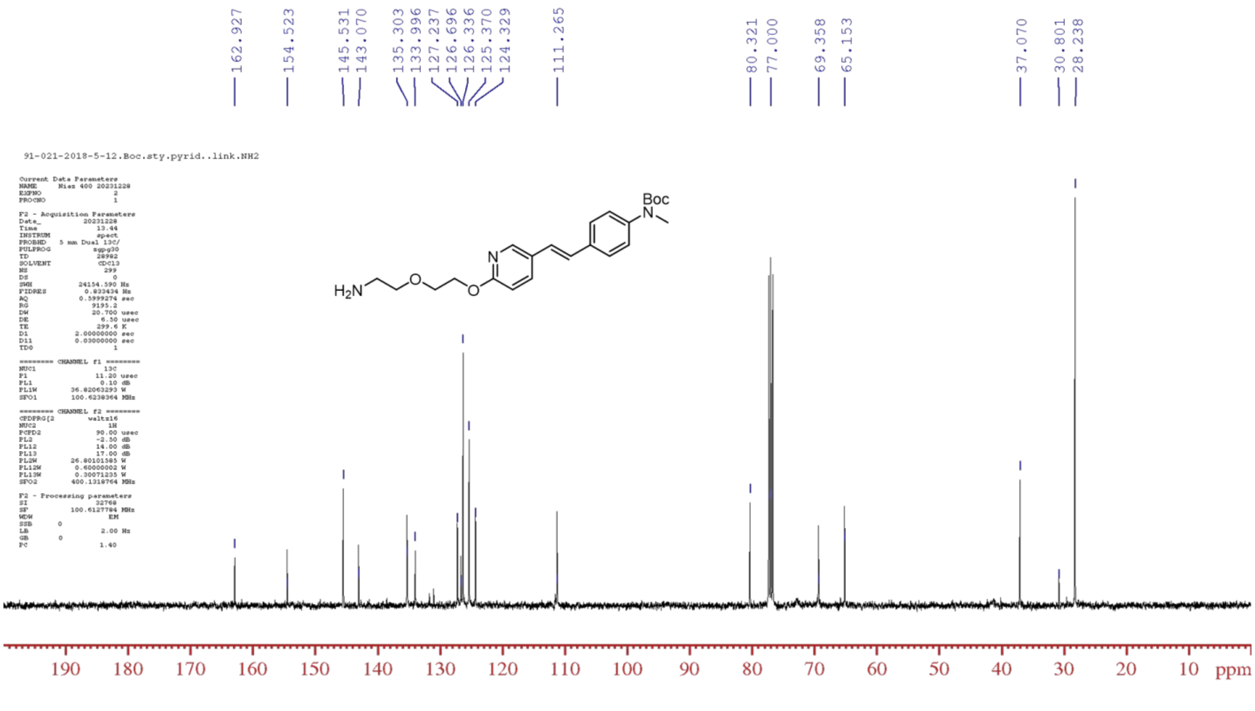

^{13}C NMR Spectrum of Compound **21** (100 MHz, $CDCl_3$)

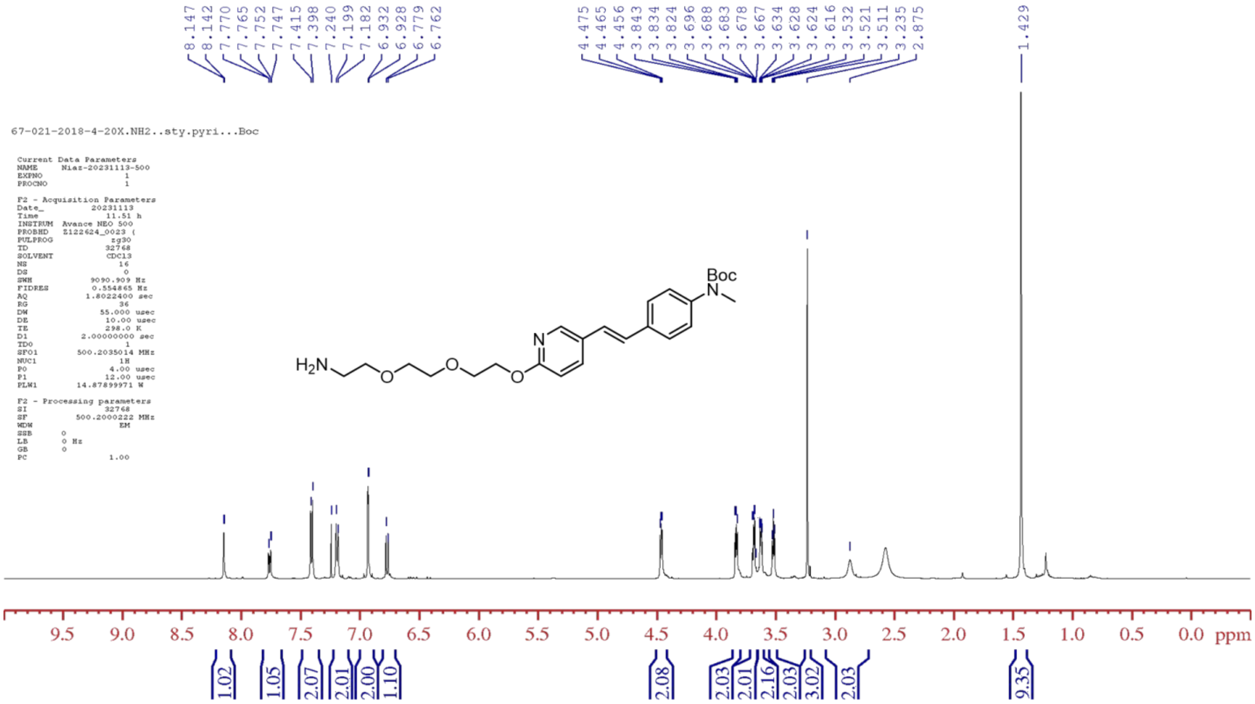

^1H NMR Spectrum of Compound **22** (500 MHz, $CDCl_3$)

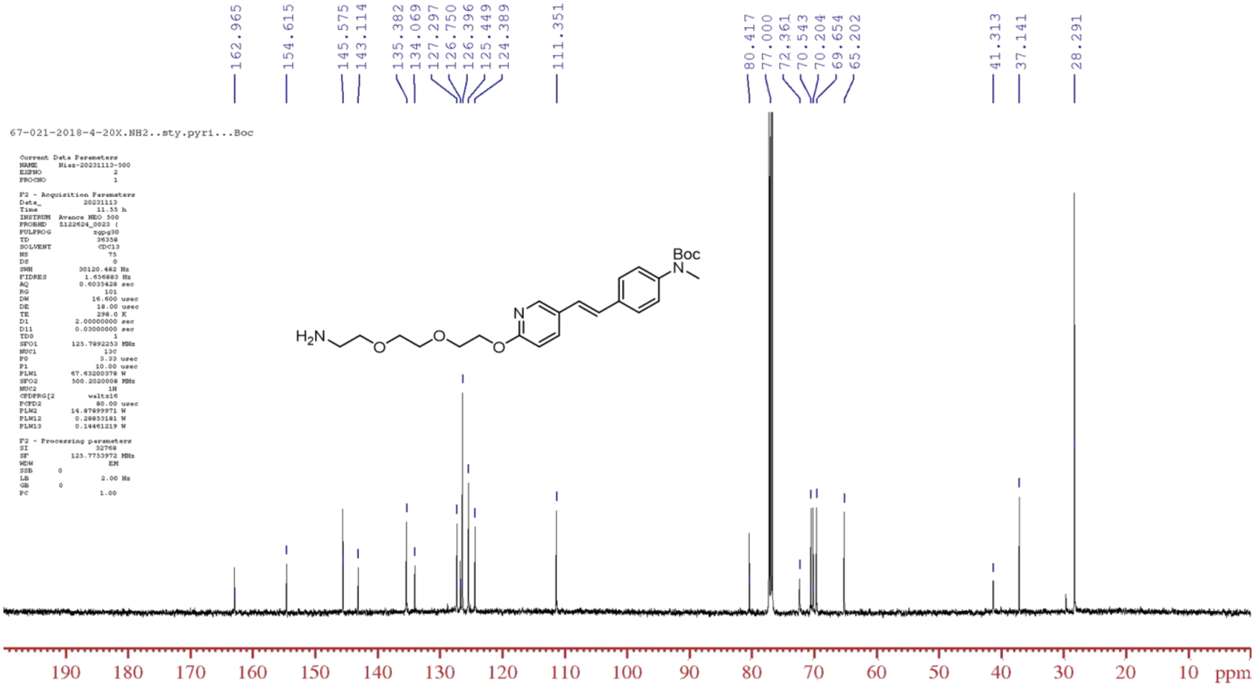

^{13}C NMR Spectrum of Compound **22** (125 MHz, $CDCl_3$)

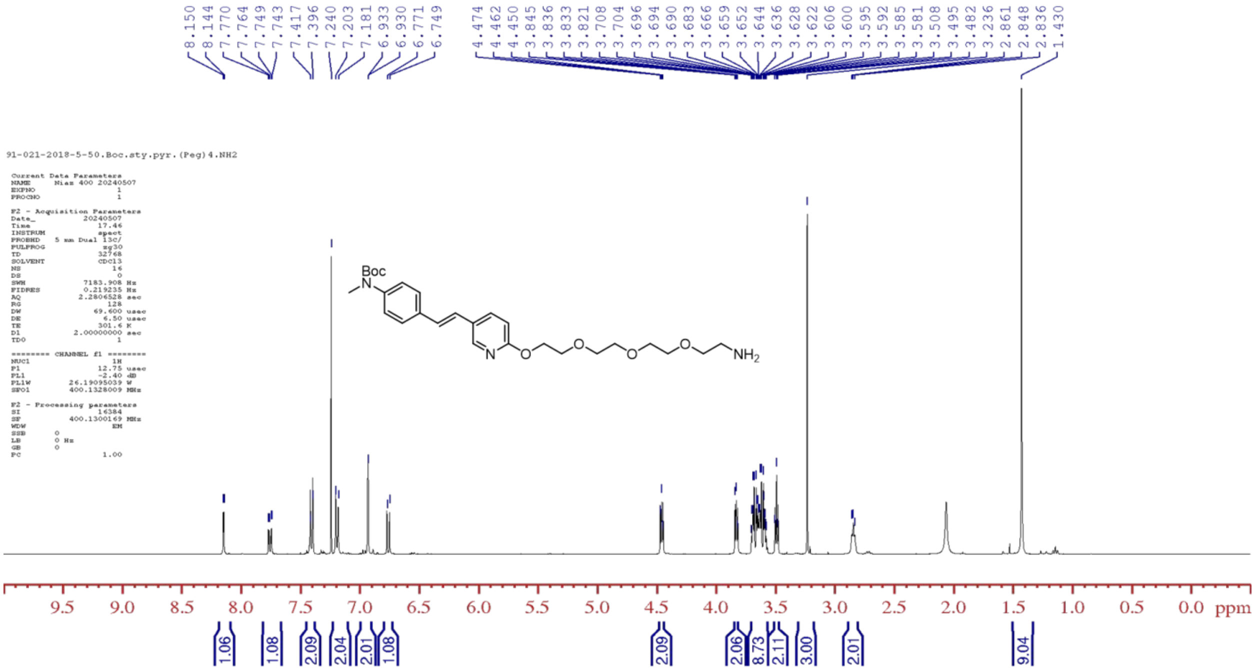

^{1}H NMR Spectrum of Compound **23** (400 MHz, $CDCl_3$)

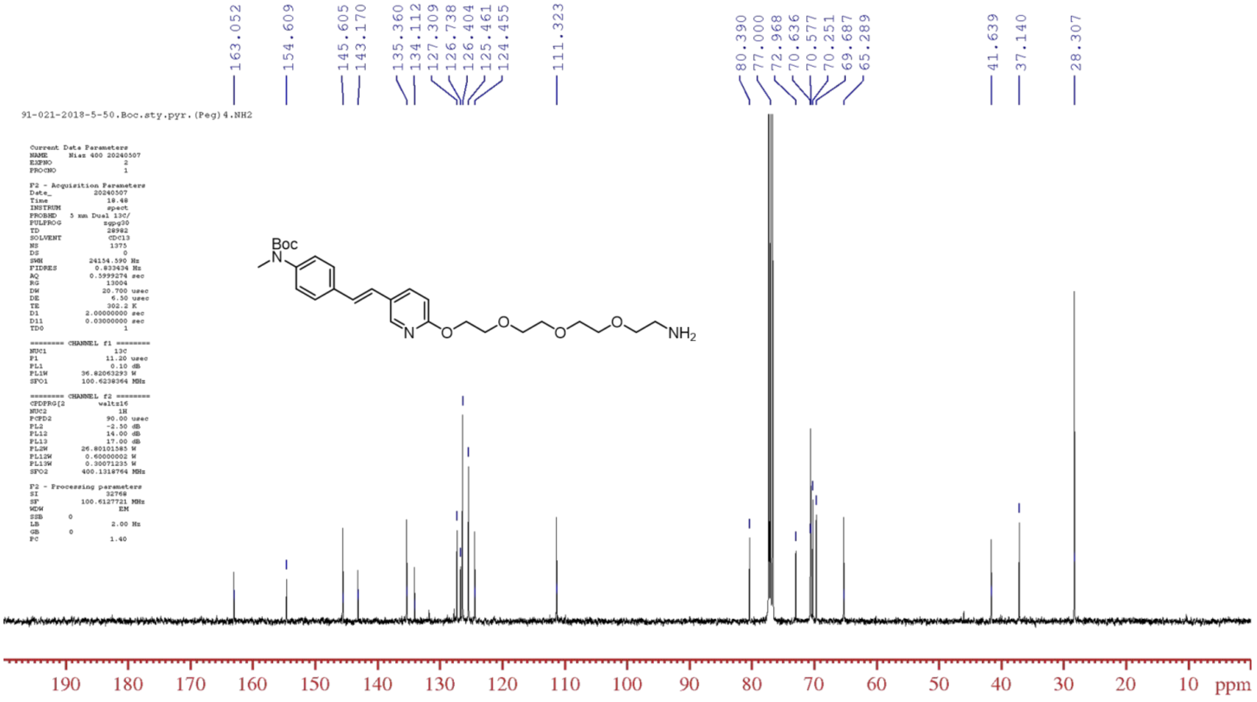

^{13}C NMR Spectrum of Compound **23** (100 MHz, $CDCl_3$)

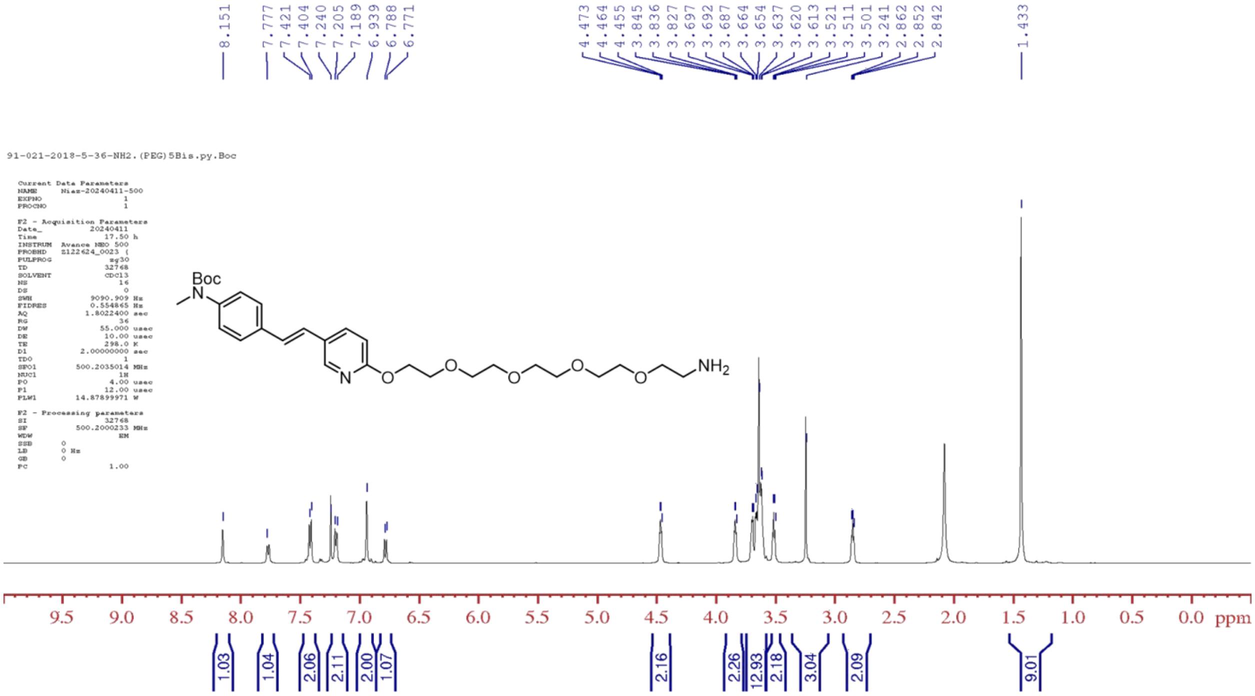

^{1}H NMR Spectrum of Compound **24** (500 MHz, $CDCl_3$)

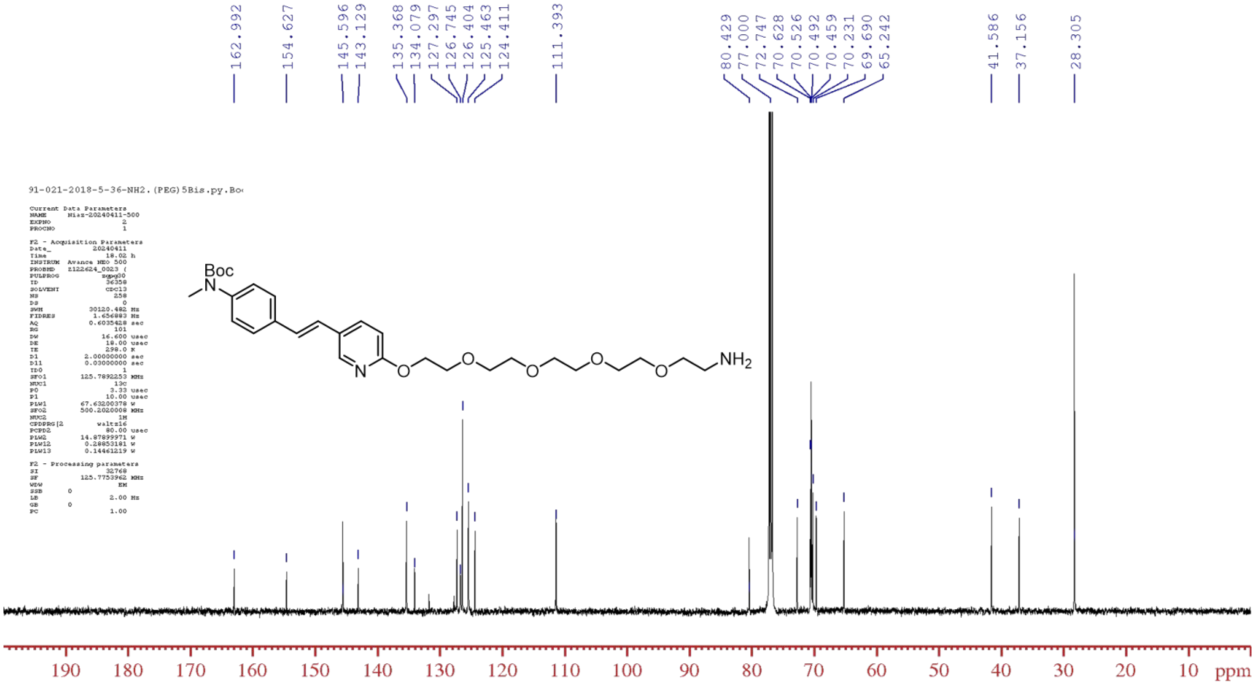

^{13}C NMR Spectrum of Compound **24** (125 MHz, $CDCl_3$)

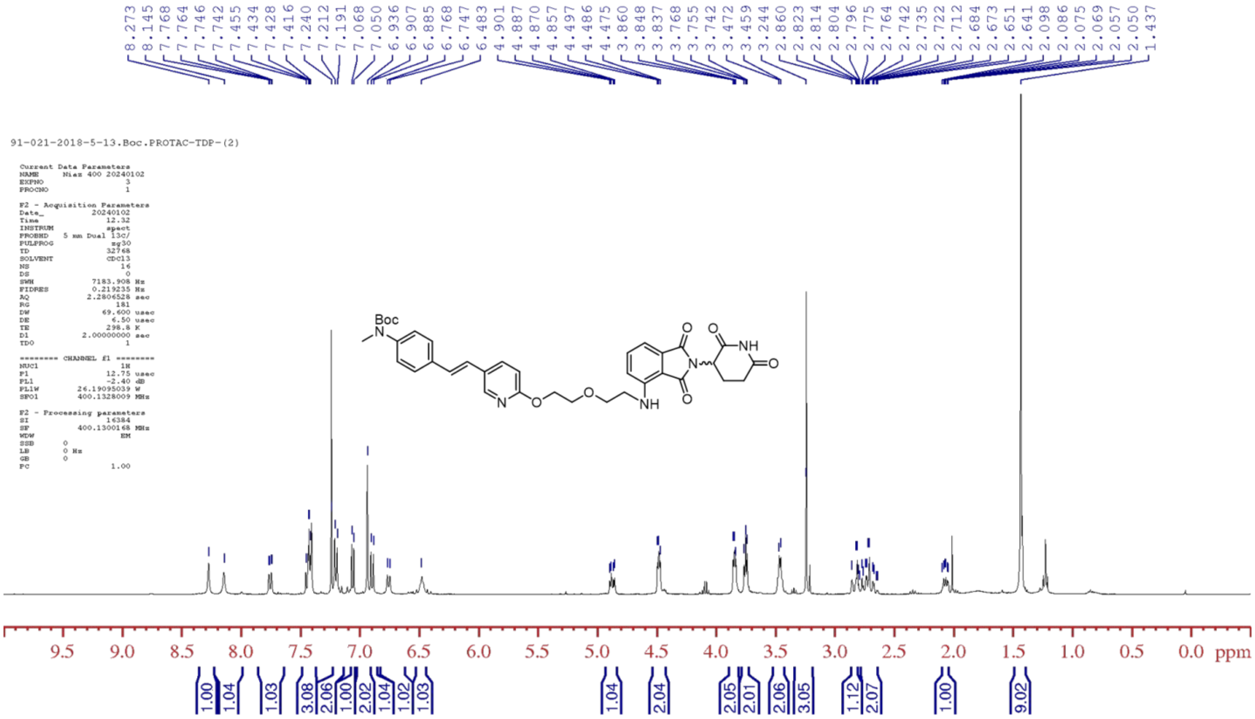

^{1}H NMR Spectrum of Compound **25** (400 MHz, $CDCl_3$)

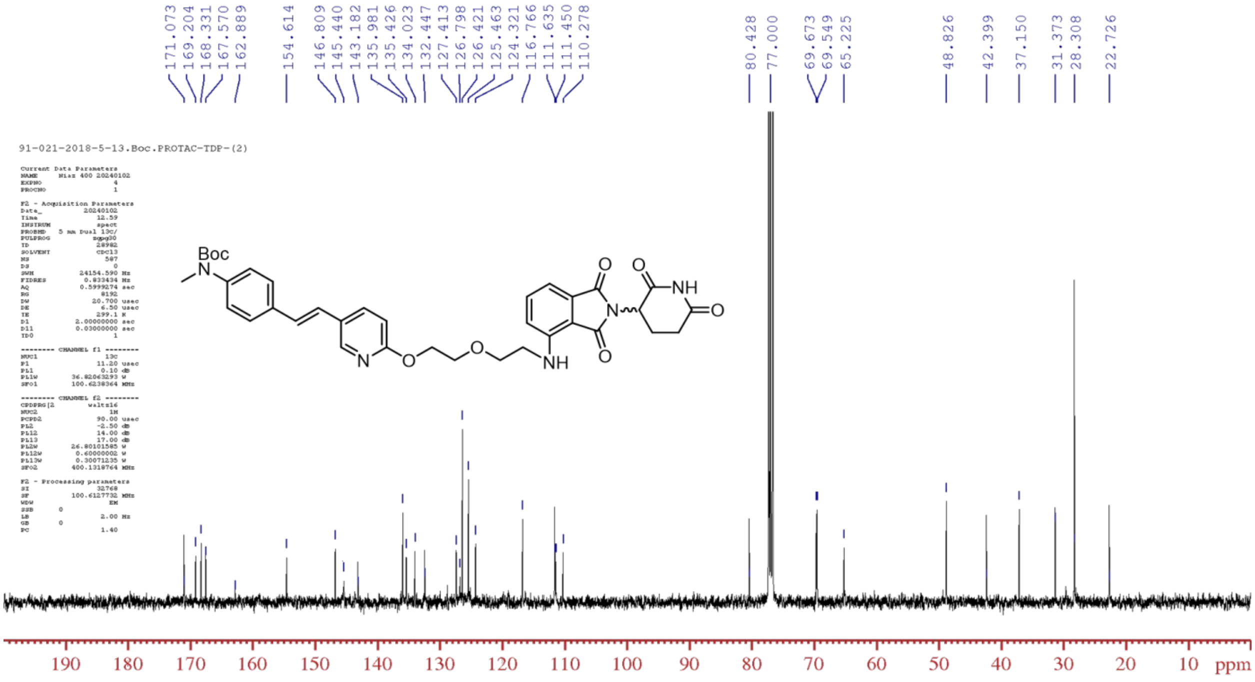

^{13}C NMR Spectrum of Compound **25** (100 MHz, $CDCl_3$)

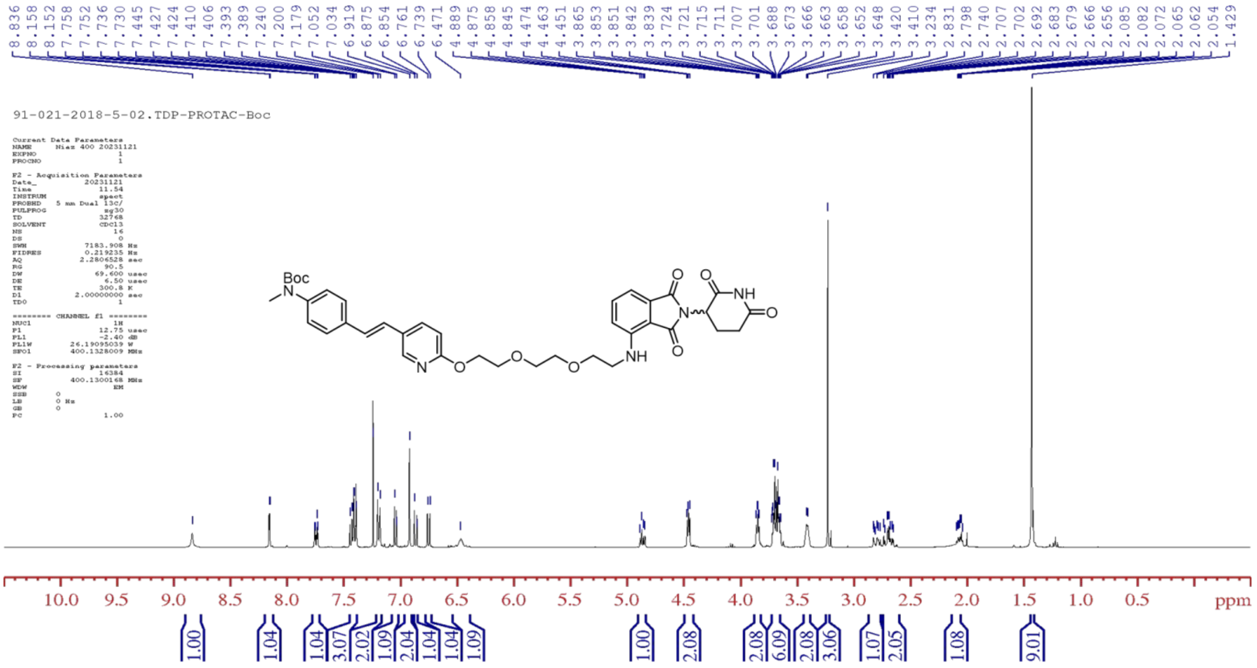

^{1}H NMR Spectrum of Compound **26** (400 MHz, $CDCl_3$)

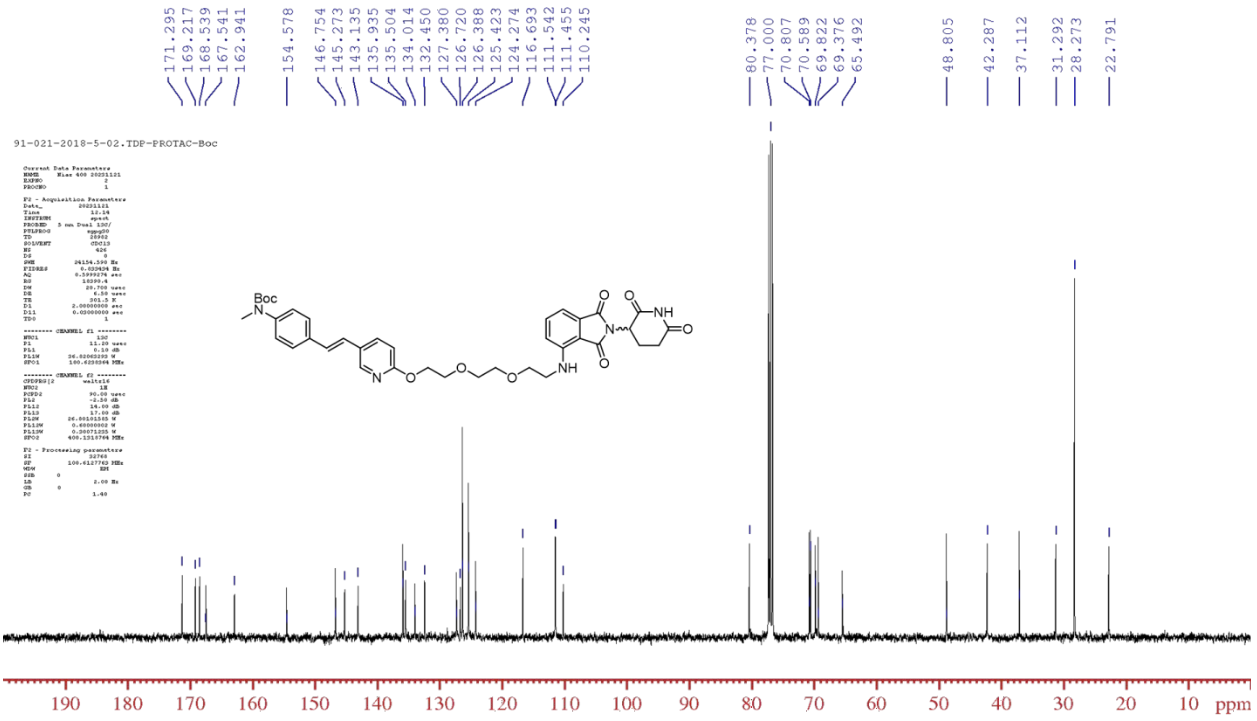

^{13}C NMR Spectrum of Compound **26** (100 MHz, $CDCl_3$)

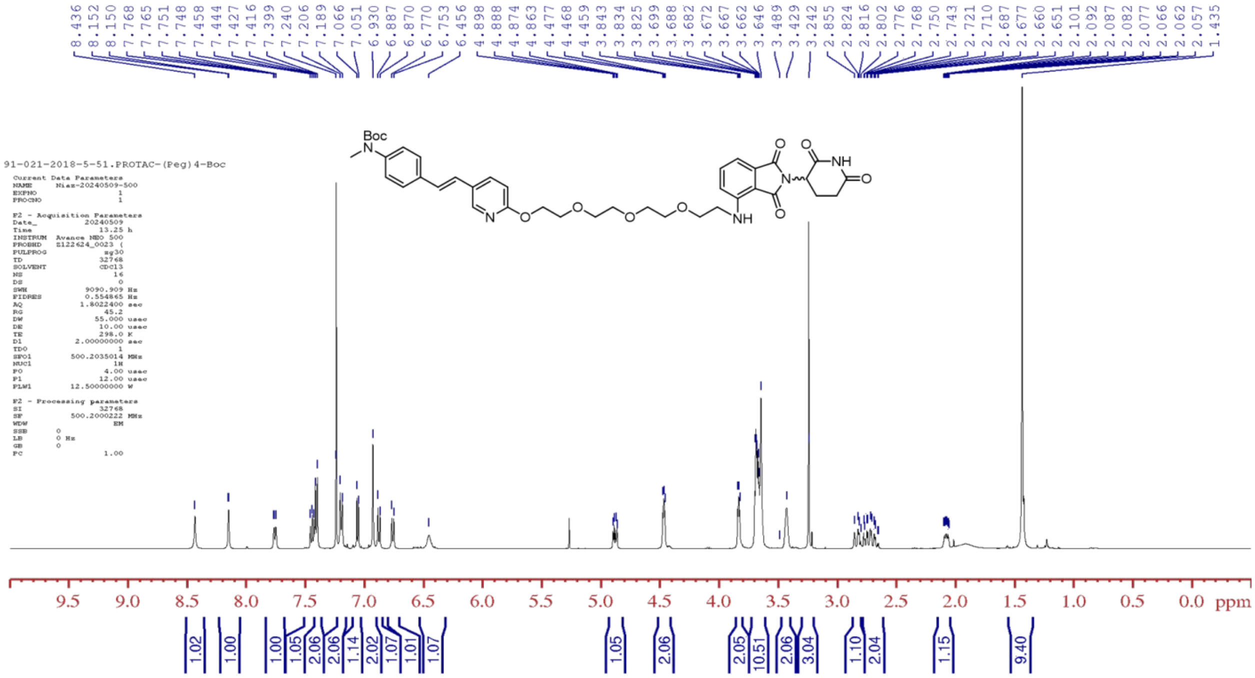

^{1}H NMR Spectrum of Compound **27** (500 MHz, $CDCl_3$)

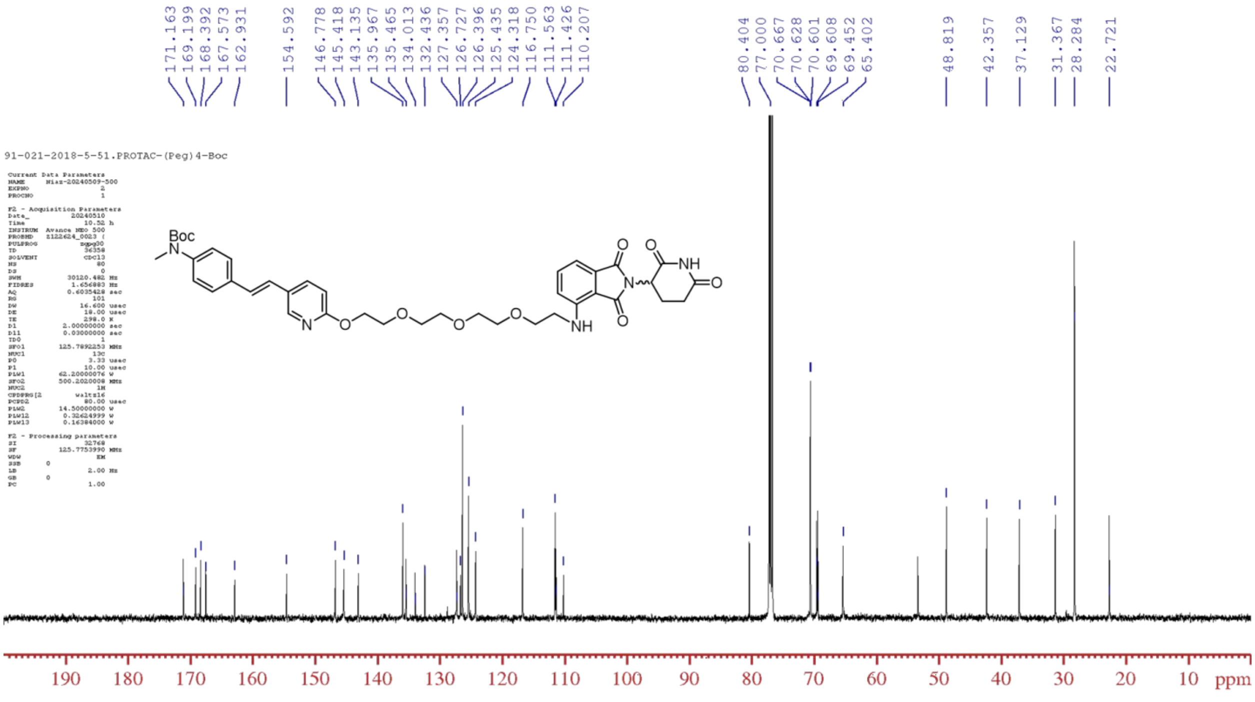

^{13}C NMR Spectrum of Compound **27** (125 MHz, $CDCl_3$)

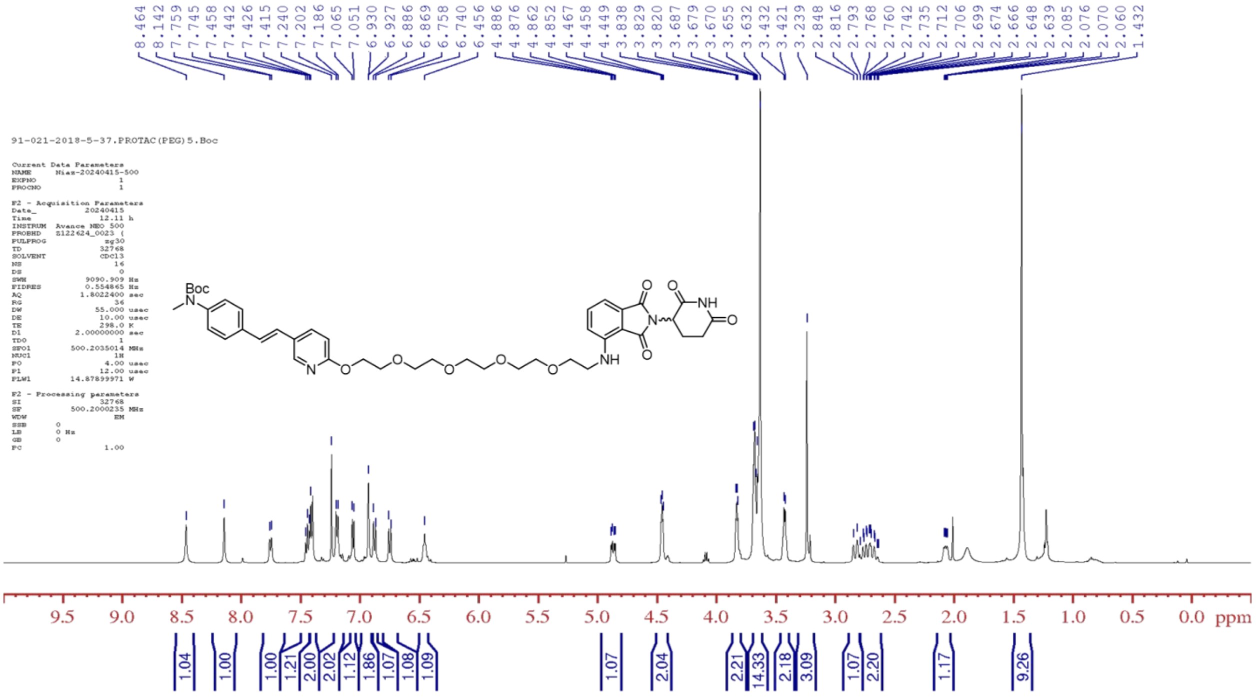

^{1}H NMR Spectrum of Compound **28** (500 MHz, $CDCl_3$)

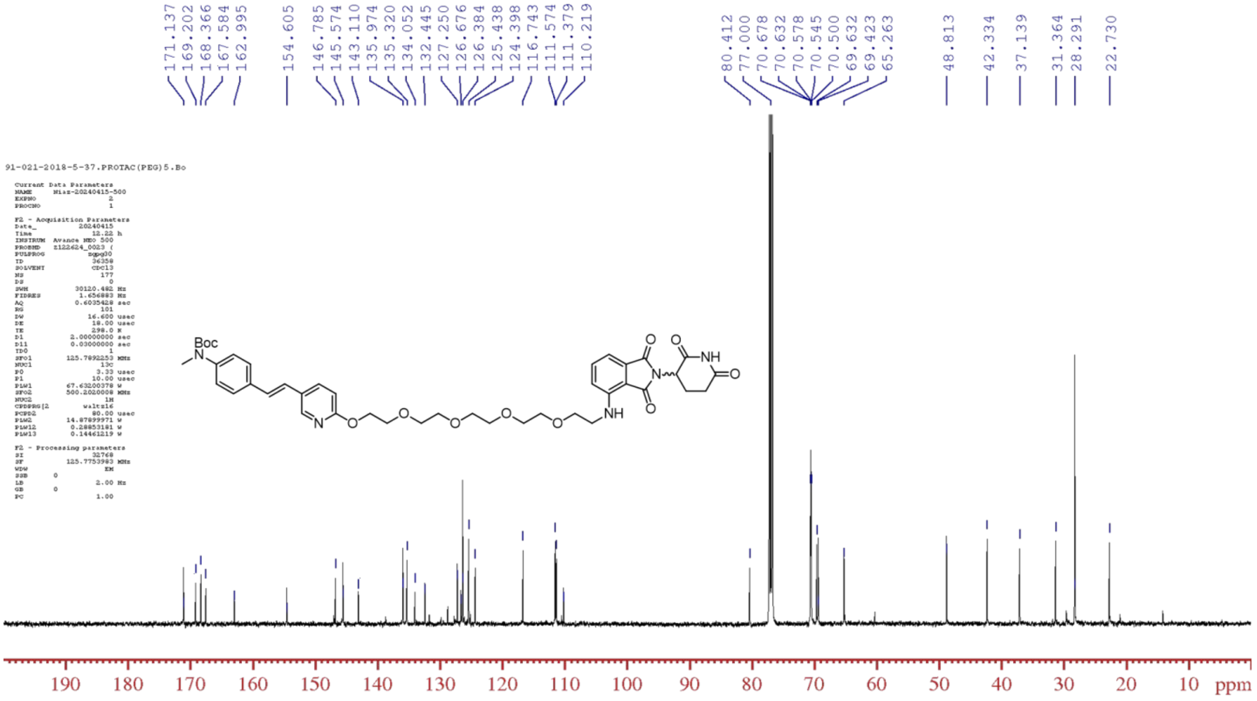

^{13}C NMR Spectrum of Compound **28** (125 MHz, $CDCl_3$)

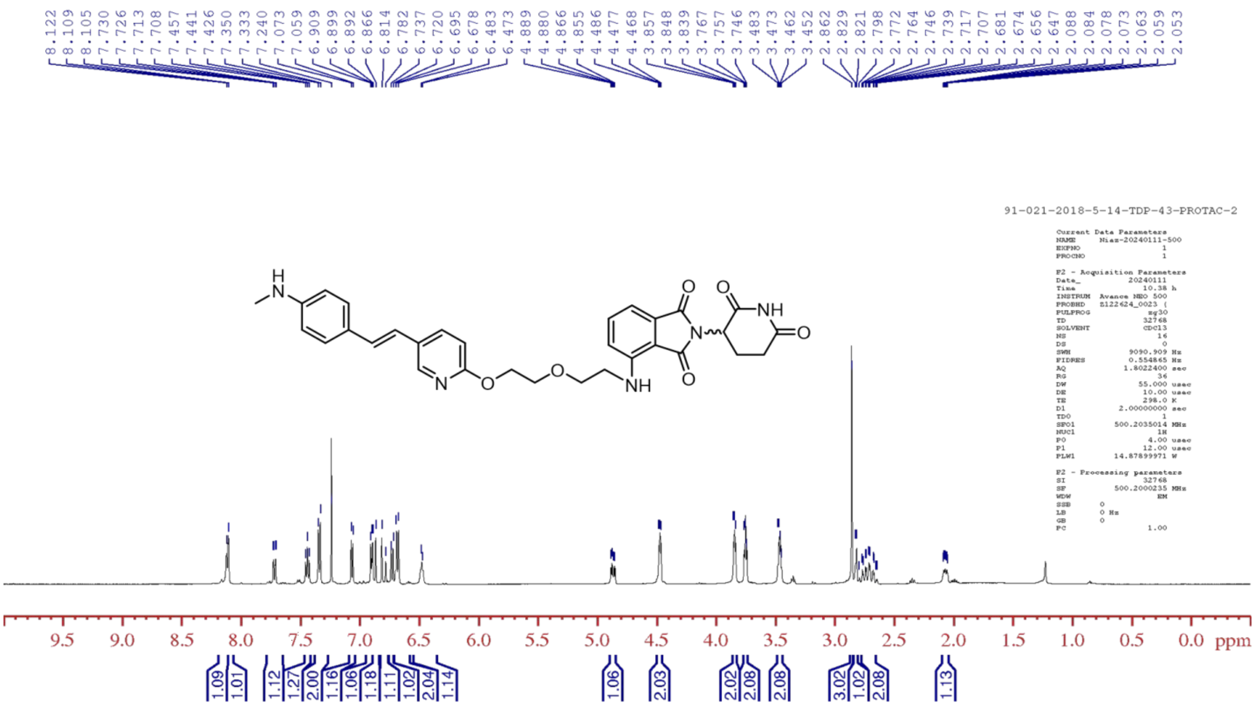

^{1}H NMR Spectrum of Compound **PROTAC 1’** (500 MHz, $CDCl_3$)

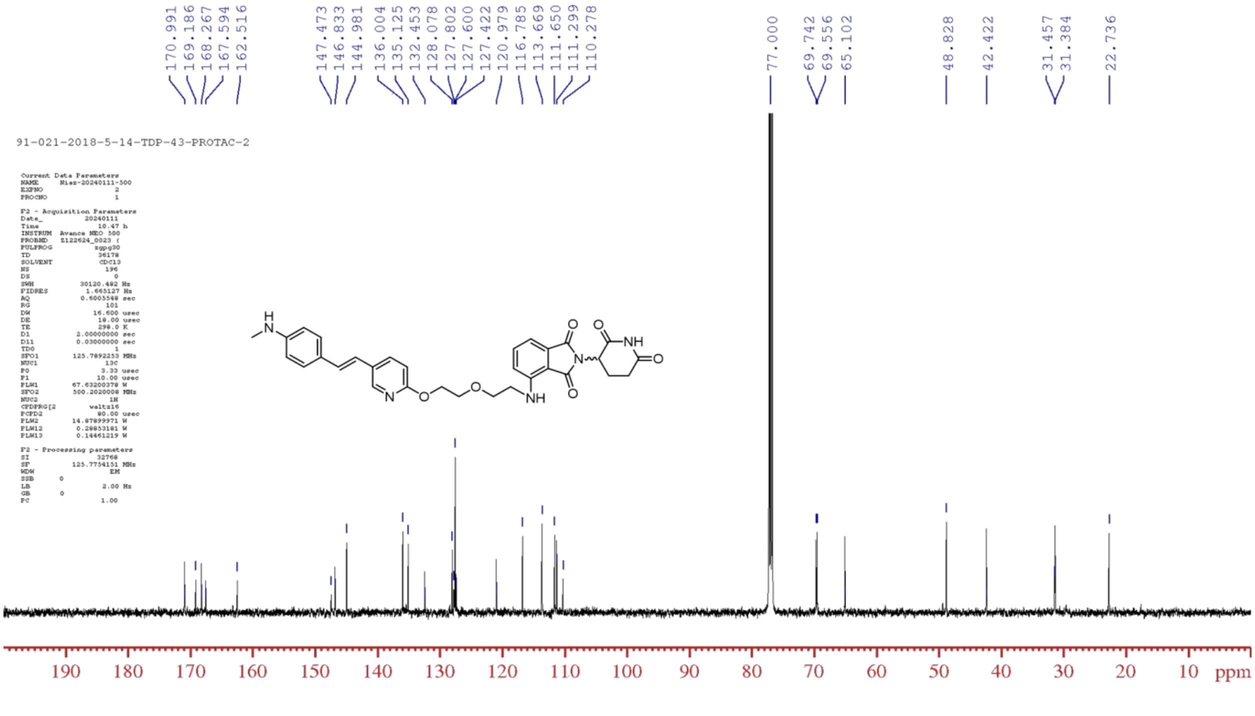

^{13}C NMR Spectrum of Compound **PROTAC 1’** (125 MHz, $CDCl_3$)

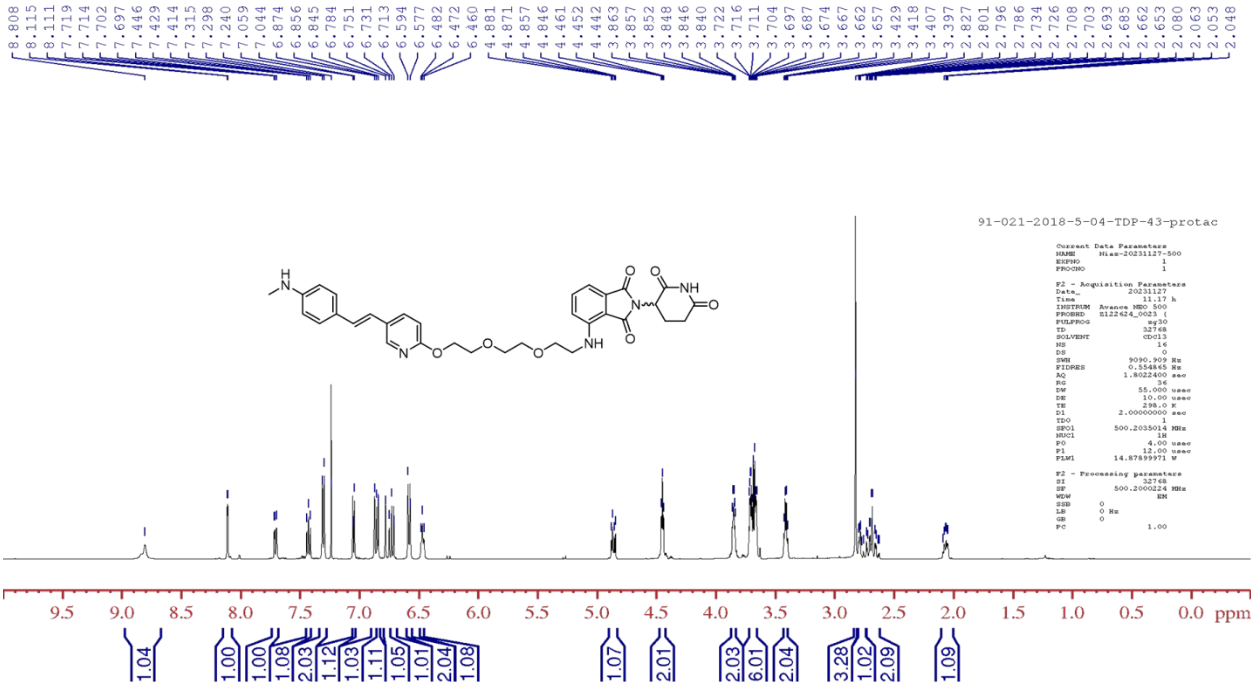

^{1}H NMR Spectrum of Compound **PROTAC 2'** (500 MHz, $CDCl_3$)

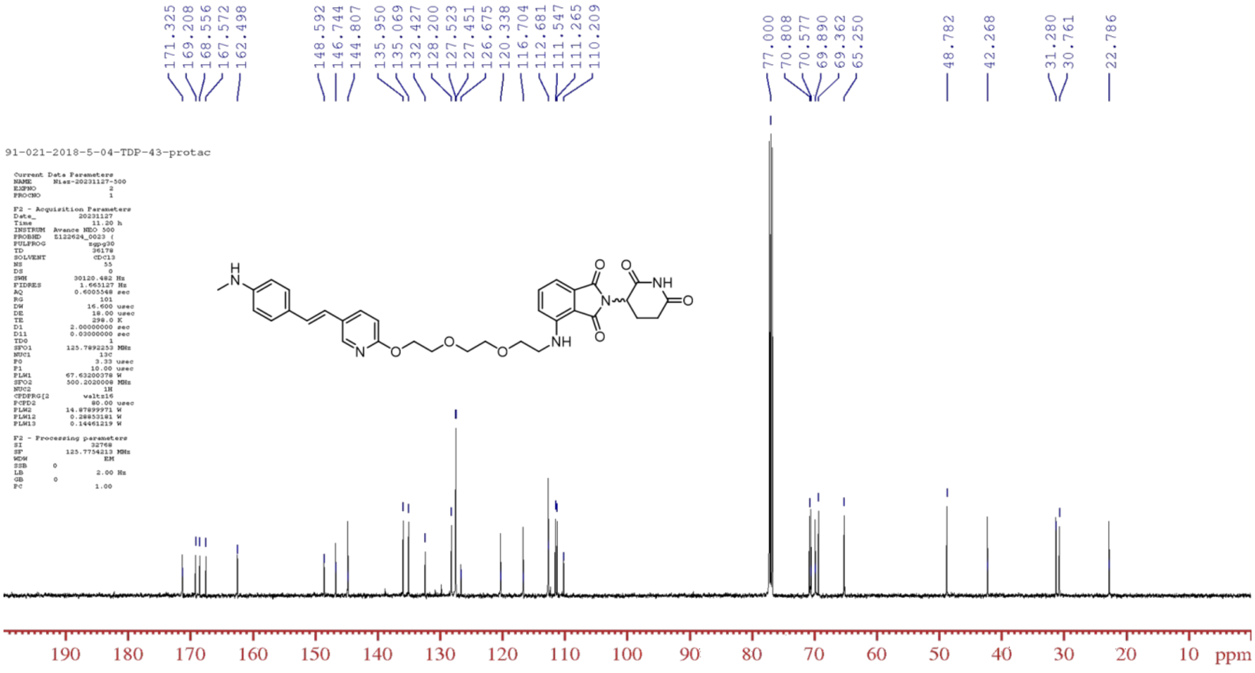

^{13}C NMR Spectrum of Compound **PROTAC 2'** (125 MHz, $CDCl_3$)

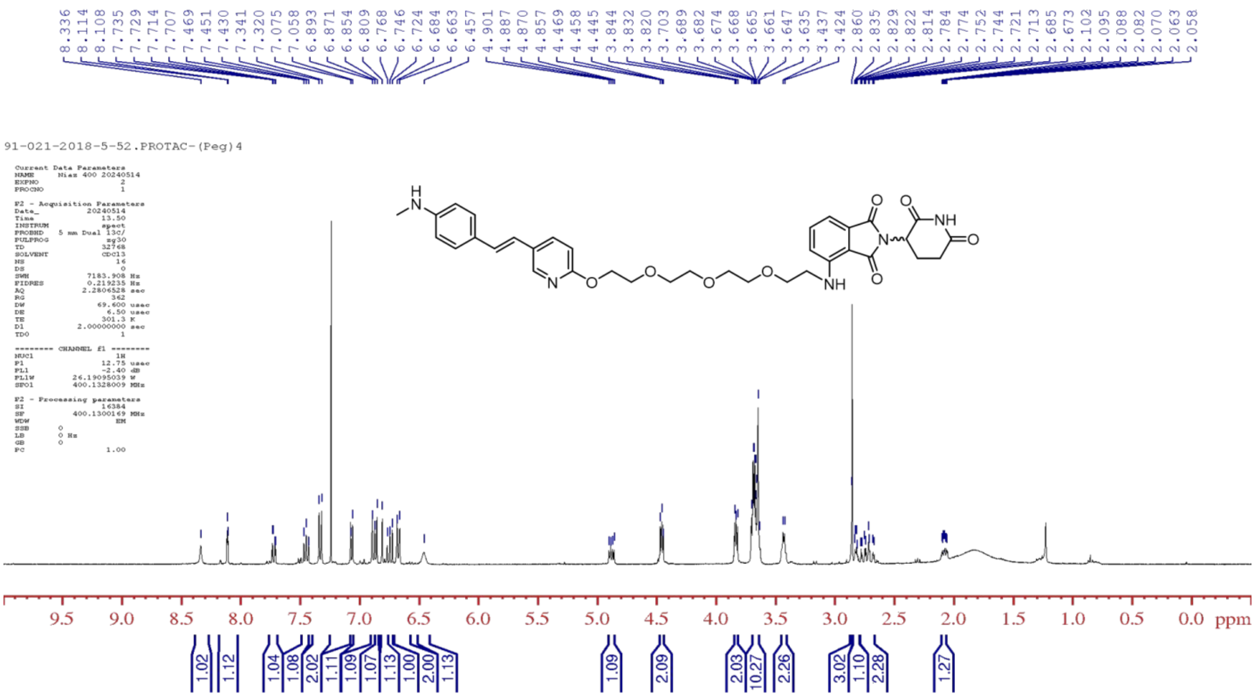

^{1}H NMR Spectrum of Compound **PROTAC 3'** (400 MHz, $CDCl_3$)

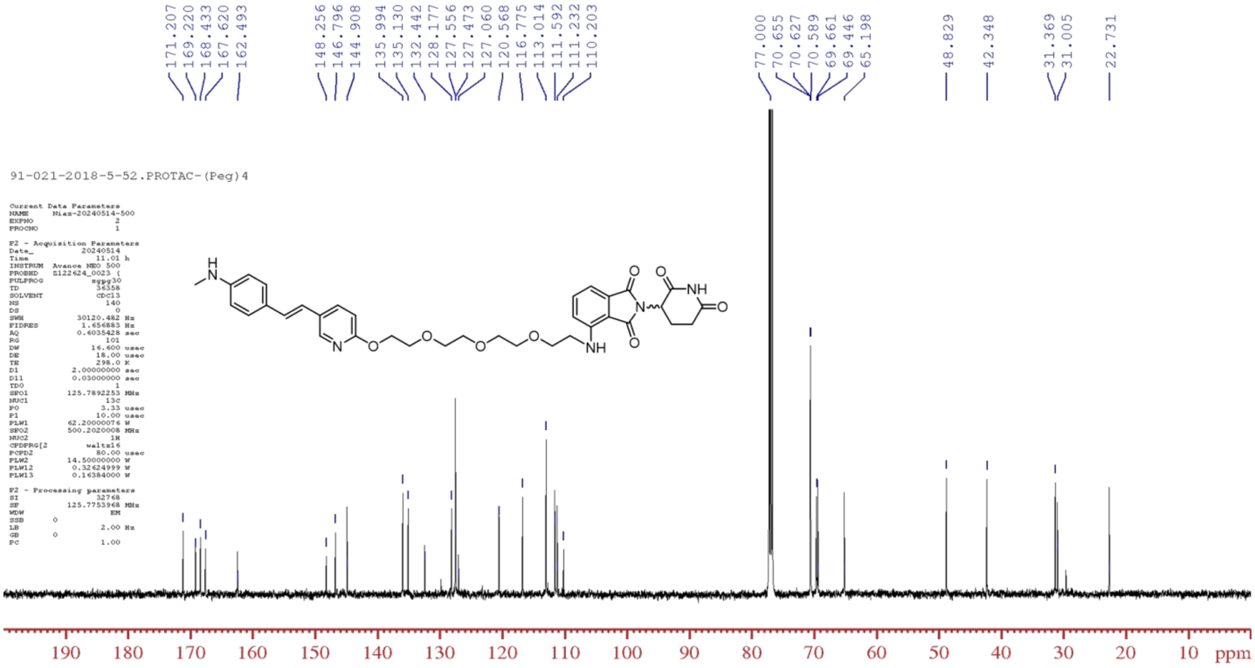

^{13}C NMR Spectrum of Compound **PROTAC 3'** (125 MHz, $CDCl_3$)

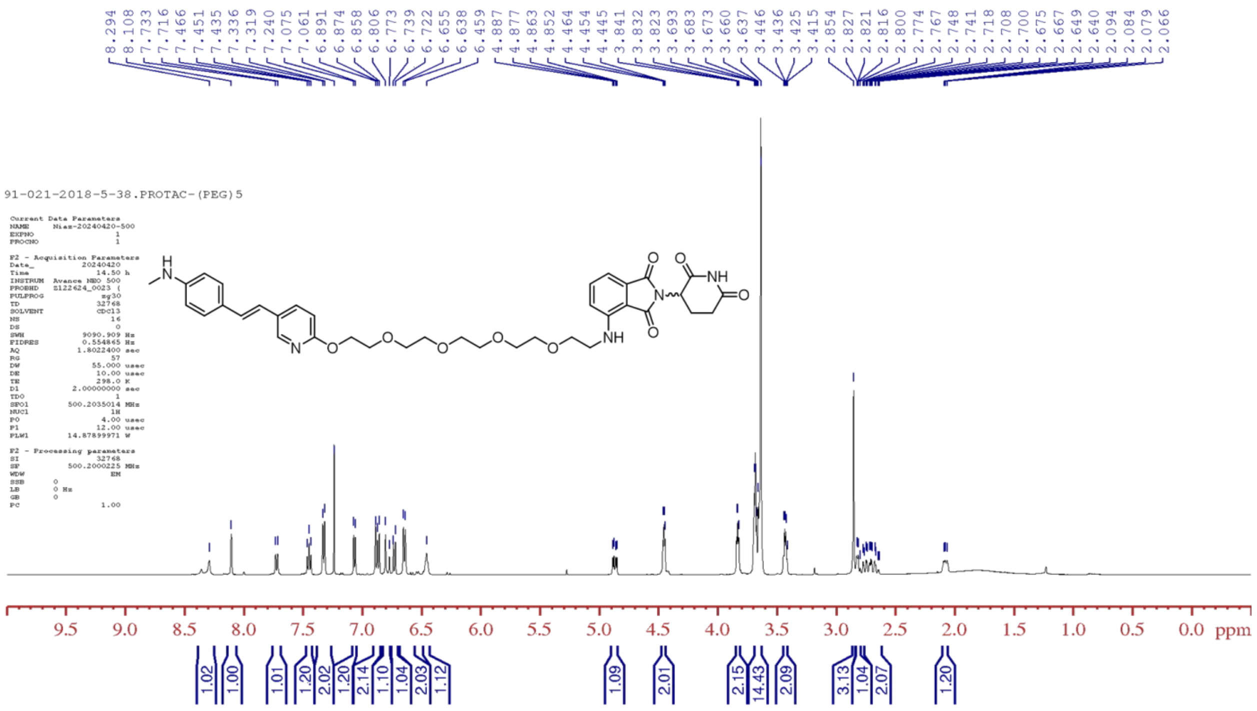

^{1}H NMR Spectrum of Compound **PROTAC 4’** (500 MHz, $CDCl_3$)

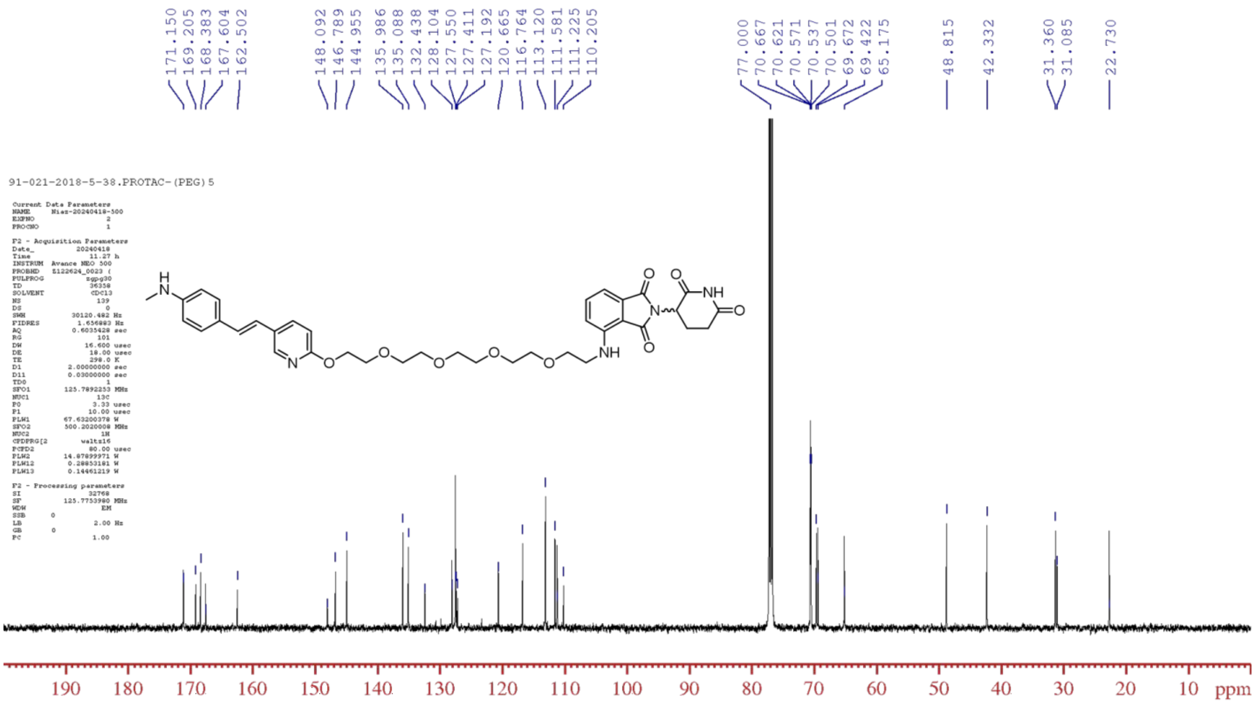

^{13}C NMR Spectrum of Compound **PROTAC 4’** (125 MHz, $CDCl_3$)

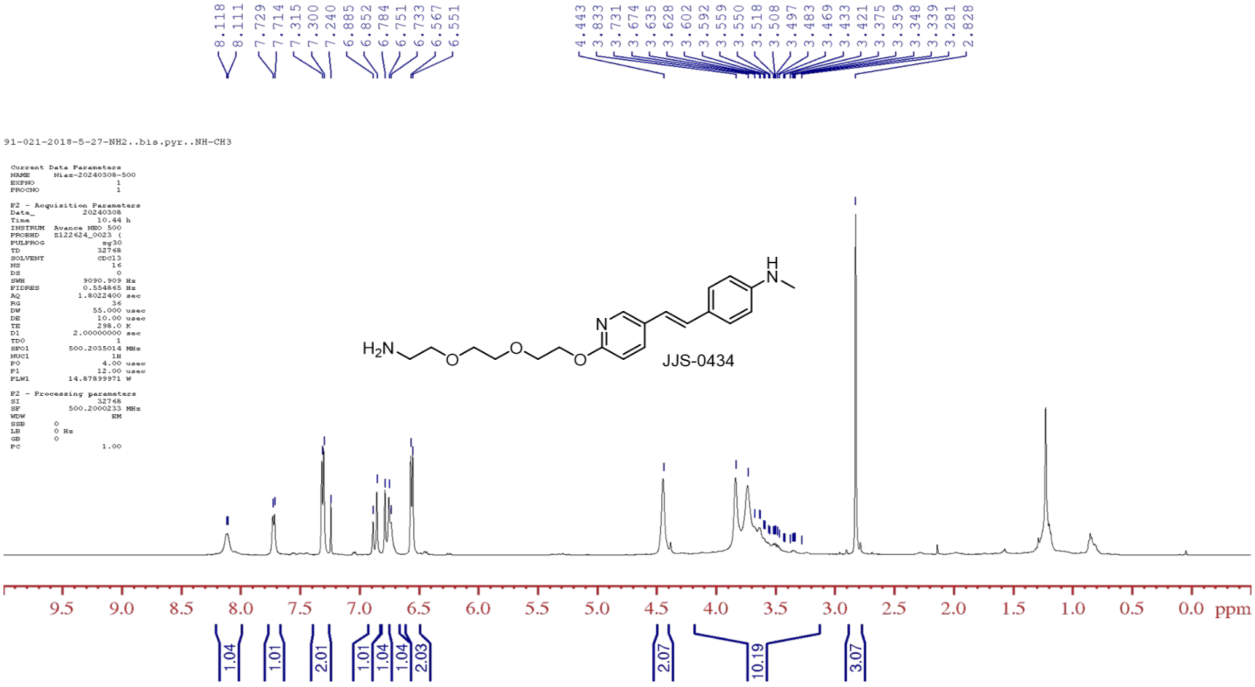

^{1}H NMR Spectrum of Compound **JJS0434** (500 MHz, $CDCl_3$)

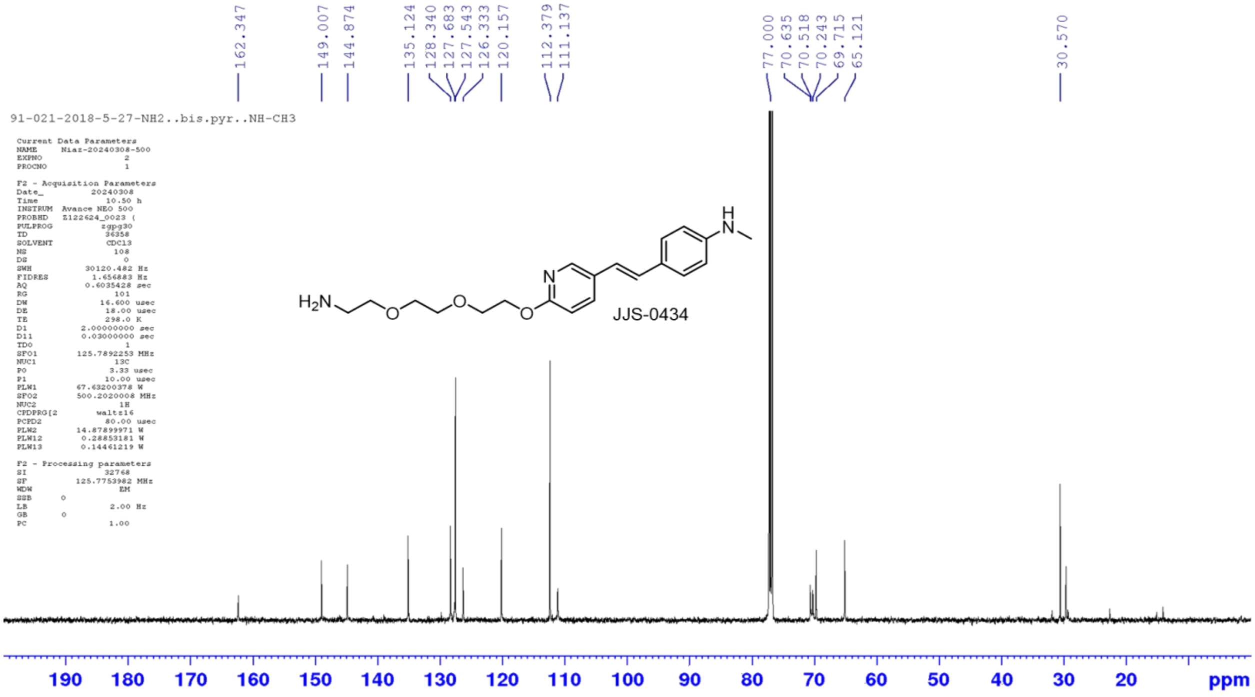

^{13}C NMR Spectrum of Compound **JJS0434** (125 MHz, $CDCl_3$)

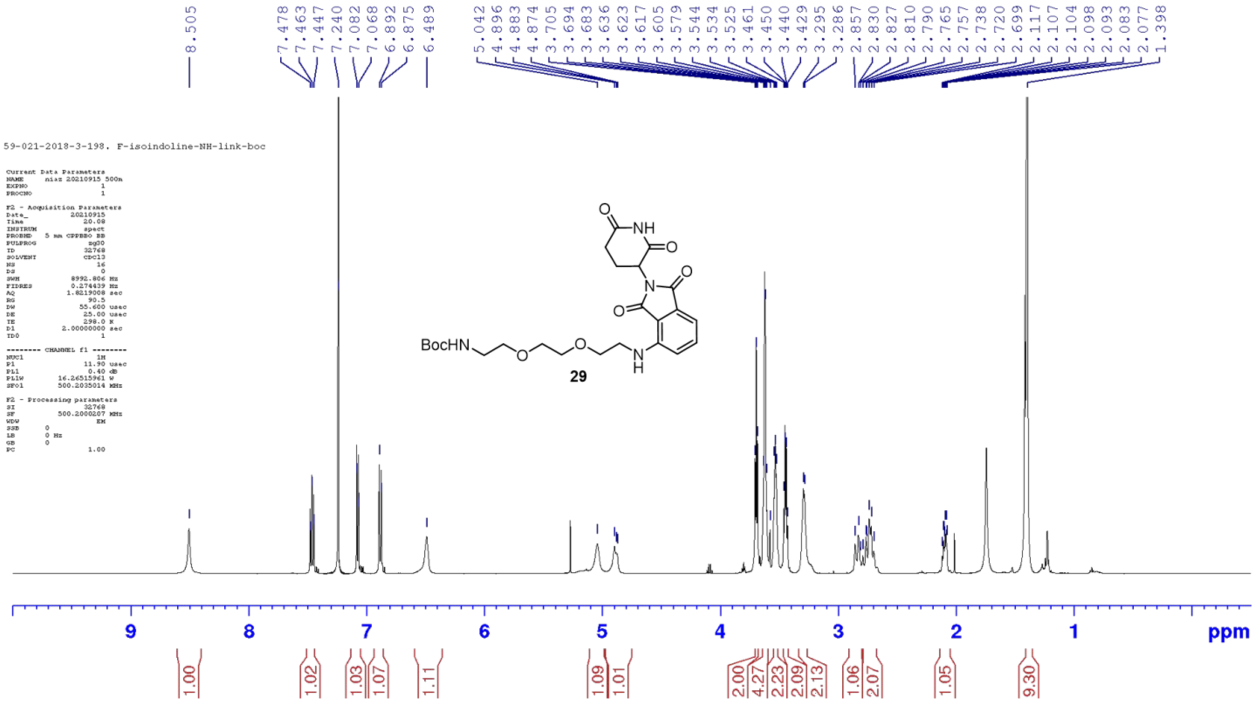

^{1}H NMR Spectrum of Compound **29** (500 MHz, $CDCl_3$)

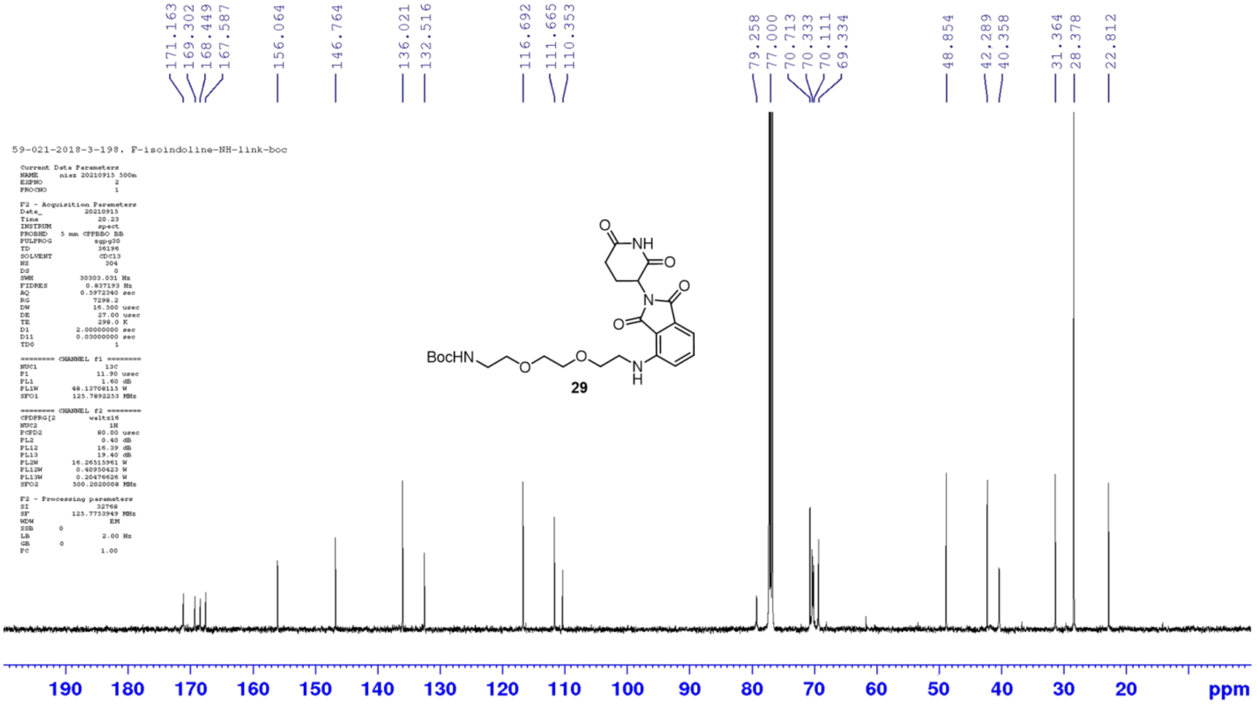

^{13}C NMR Spectrum of Compound **29** (125 MHz, $CDCl_3$)

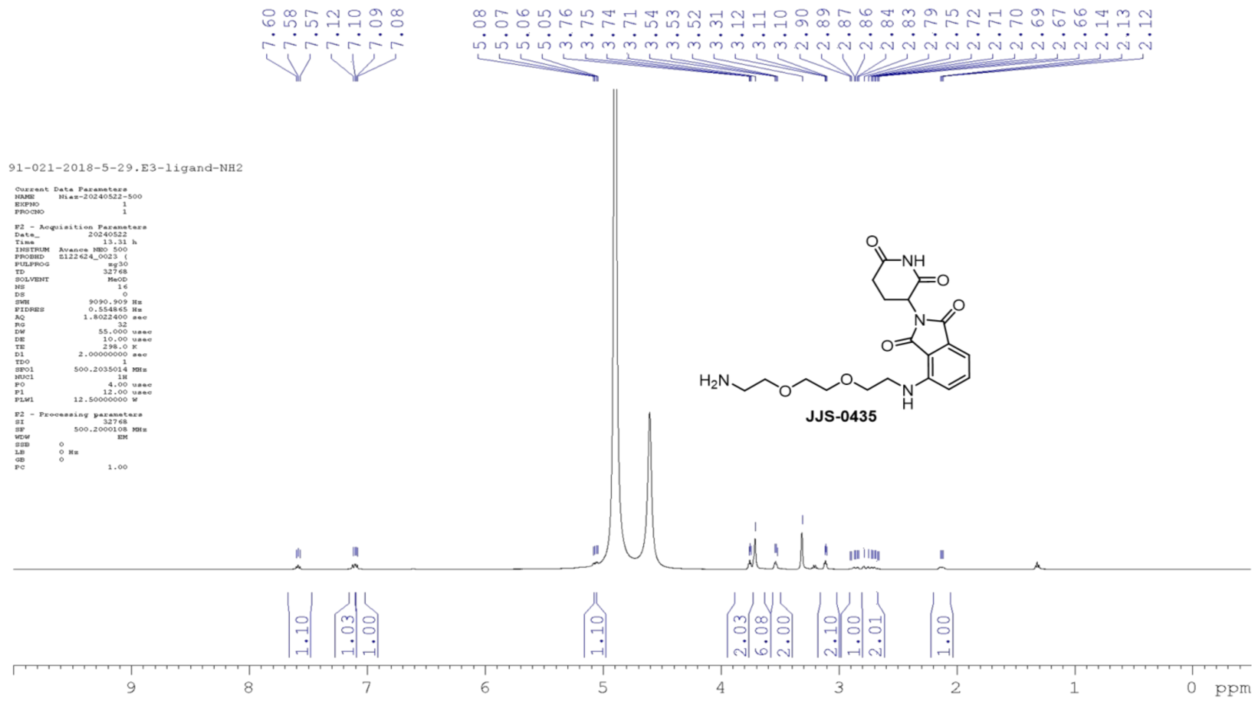

^{1}H NMR Spectrum of Compound **JJS0435** (500 MHz, CD_3OD)

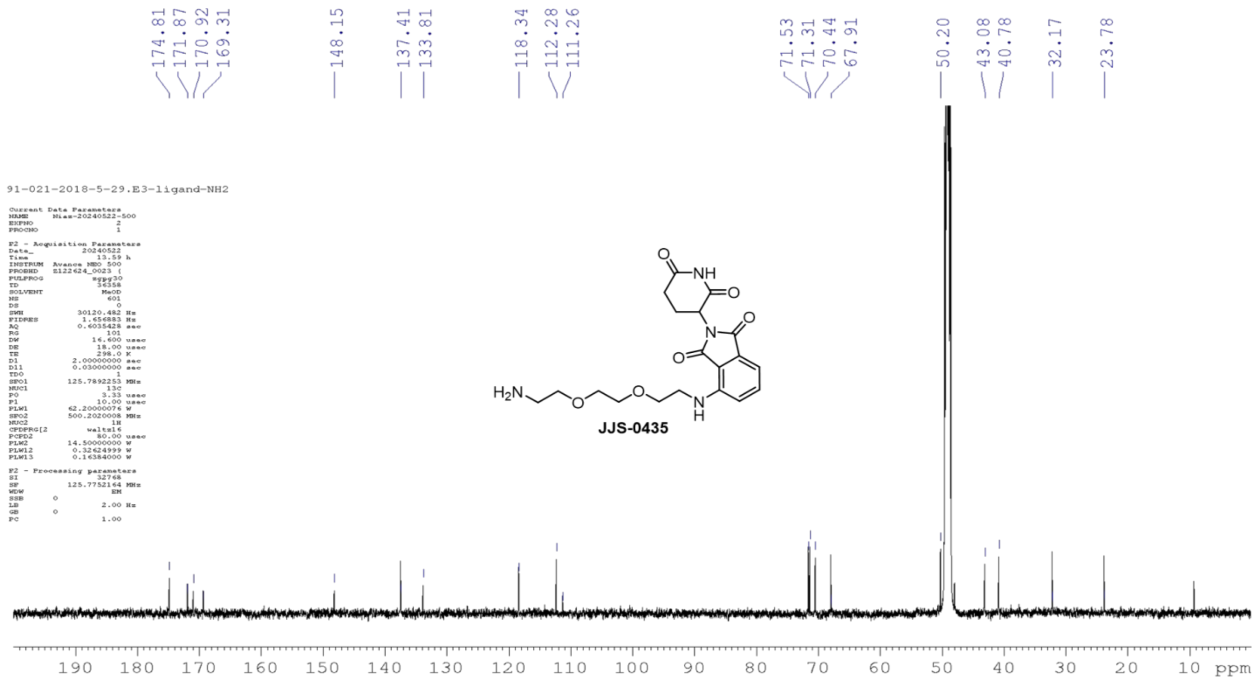

^{13}C NMR Spectrum of Compound **JJS0435** (125 MHz, CD_3OD)
